# Rational Design of Highly Potent SARS-CoV-2 nsp14 Methyltransferase Inhibitors

**DOI:** 10.1021/acsomega.3c02815

**Published:** 2023-07-21

**Authors:** Milan Štefek, Dominika Chalupská, Karel Chalupský, Michala Zgarbová, Alexandra Dvořáková, Petra Krafčíková, Alice Shi Ming Li, Michal Šála, Milan Dejmek, Tomáš Otava, Ema Chaloupecká, Jaroslav Kozák, Ján Kozic, Masoud Vedadi, Jan Weber, Helena Mertlíková-Kaiserová, Radim Nencka

**Affiliations:** † Institute of Organic Chemistry and Biochemistry of the Czech Academy of Sciences, Flemingovo Náměstí 2, Prague 6 166 10, Czech Republic; ‡ Department of Organic Chemistry, Faculty of Science, Charles University, Prague 128 00, Czech Republic; § Department of Pharmacology and Toxicology, University of Toronto, Toronto, Ontario M5S 1A8, Canada; ∥ QBI COVID-19 Research Group (QCRG), San Francisco, California 94158, United States

## Abstract

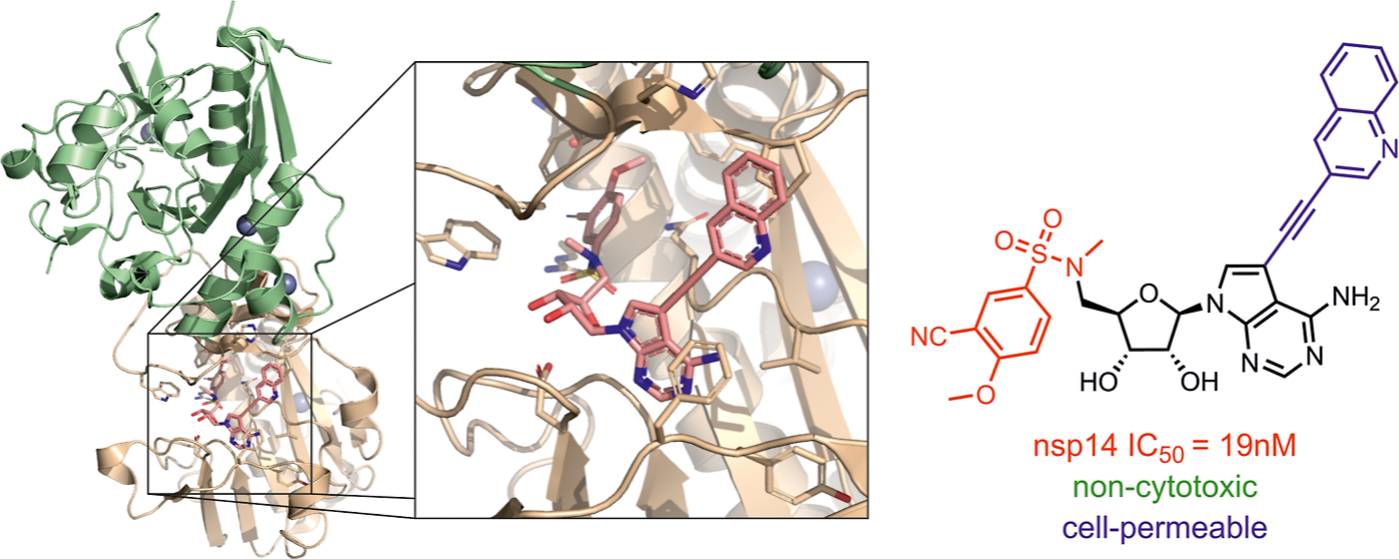

The search for new drugs against COVID-19 and its causative agent, SARS-CoV-2, is one of the major trends in the current medicinal chemistry. Targeting capping machinery could be one of the therapeutic concepts based on a unique mechanism of action. Viral RNA cap synthesis involves two methylation steps, the first of which is mediated by the nsp14 protein. Here, we rationally designed and synthesized a series of compounds capable of binding to both the *S*-adenosyl-l-methionine and the RNA-binding site of SARS-CoV-2 nsp14 *N*^7^-methyltransferase. These hybrid molecules showed excellent potency, high selectivity toward various human methyltransferases, nontoxicity, and high cell permeability. Despite the outstanding activity against the enzyme, our compounds showed poor antiviral performance in vitro. This suggests that the activity of this viral methyltransferase has no significant effect on virus transcription and replication at the cellular level. Therefore, our compounds represent unique tools to further explore the role of the SARS-CoV-2 nsp14 methyltransferase in the viral life cycle and the pathogenesis of COVID-19.

## Introduction

COVID-19 is an infectious disease caused by one of the seven human-affecting coronaviruses called severe acute respiratory syndrome coronavirus 2 (SARS-CoV-2).^1^ Coronaviruses belong to positive-sense single-stranded RNA (+ssRNA) viruses and are among the largest of them containing highly complex genomes. In particular, the SARS-CoV-2 genome has 29.9 kb with 14 open reading frames (ORFs) encoding 29 viral proteins.^2,3^ The first two ORFs (ORF1a and ORF1b) alone contain information for two large polyproteins, pp1a and pp1ab, which are subsequently cleaved into 16 nonstructural proteins (nsps) that play essential roles in SARS-CoV-2 replication.^4^

In order to deceive and exploit the human translation apparatus and immune system, viral RNA must contain a specific structure called “cap” at its 5′ end. While eukaryotic mRNAs are endowed with this cap already in the nucleus, most RNA viruses do not have access to the nucleus and therefore cannot use the cellular machinery to synthesize this defensive and functional structure. Different types of RNA viruses use different procedures to install this cap.^5,6^ Coronaviruses mimic the procedure used by cells and install the cap in several consecutive steps.^7^ First, they attach GTP to the newly formed RNA, presumably by a sophisticated cooperation of nsp12 and nsp9.^3,8^ Then, they methylate the GTP nucleobase at position 7 using the nsp14 protein,^9^ and finally, they install a methyl group on the 2′-hydroxyl group of the first following nucleoside using the nsp16 protein activated by nsp10.^10^ Both of these methyltransferases (nsp14 and nsp16-nsp10) use an *S*-adenosyl-l-methionine (SAM) as a methylation reagent from which the reaction byproduct *S*-adenosyl-l-homocysteine (SAH) is produced. The binding sites for SAM are significantly conserved between related coronaviruses in nsp14 as well as in nsp16.^11,12^ In addition to its function as a methyltransferase (MTase), nsp14 in a complex with nsp10 also has an exonuclease function and is able, to some extent, to repair incorrectly synthesized viral RNA and thus prevent excessive mutation of such a large genome.^13^

The importance of the nsp14 MTase activity for viral life cycle has been demonstrated on several coronaviruses. Case and co-workers proved that a mutation in the SAM-binding site of murine hepatitis virus (MHV) nsp14, a model for SARS-CoV, significantly affected the replication kinetics, decreased peak titer of the virus, and increased sensitivity to interferon-based immune response. They also suggested that the role of nsp14 in vivo might be more important than its effect on viral replication in vitro.^14^ Recently, Pan et al. followed up on this study with experiments performed in vivo on both MHV and SARS-CoV-2 with mutant nsp14 MTase, demonstrating that the virus thus affected is significantly attenuated and that this effect takes place very early in the life cycle. Their data also suggests that these effects are associated with a substantial alteration of the immune response.^15^ The nsp14 MTase, therefore, plays an important role in the life cycle of coronaviruses influencing their replication efficiency and significantly interferes with the immune response to infection with these viruses. This is why the SARS-CoV-2 nsp14 MTase has become an attractive target for medicinal chemistry research,^7^ complementing traditional targets for antiviral therapy such as RNA-dependent RNA polymerase or viral proteases.

The main types of nsp14 inhibitors that are currently being developed are inhibitors that bind to the SAM-binding site and compete with this methyl group source. One of the first agents shown to have activity against coronavirus nsp14 MTase is sinefungin, which, despite its low selectivity, is used as a benchmark.^7,16^ In the case of direct SAM/SAH analogues, modification of the amino acid moiety, either as an amino acid bioisostere or as a bisubstrate inhibitor targeting both SAM- and RNA-binding sites, has emerged as an important strategy.^17−21^ Our group’s research explored possible modifications on the purine nucleobase,^22^ a strategy which has recently been used by other authors.^17,23^ Intriguing result was also obtained by fragment crystallography and high-throughput screening.^24−27^

Here, we follow up on our nucleobase modification study of SAH analogues, and we combine it with the results obtained by substitution of the amino acid part of SAH analogues. Thus, we produced highly active inhibitors of SARS-CoV-2 nsp14 MTase, which do not have a zwitterionic character and can penetrate into cells.

## Results and Discussion

### Design of New Compounds

**Figure 1:**
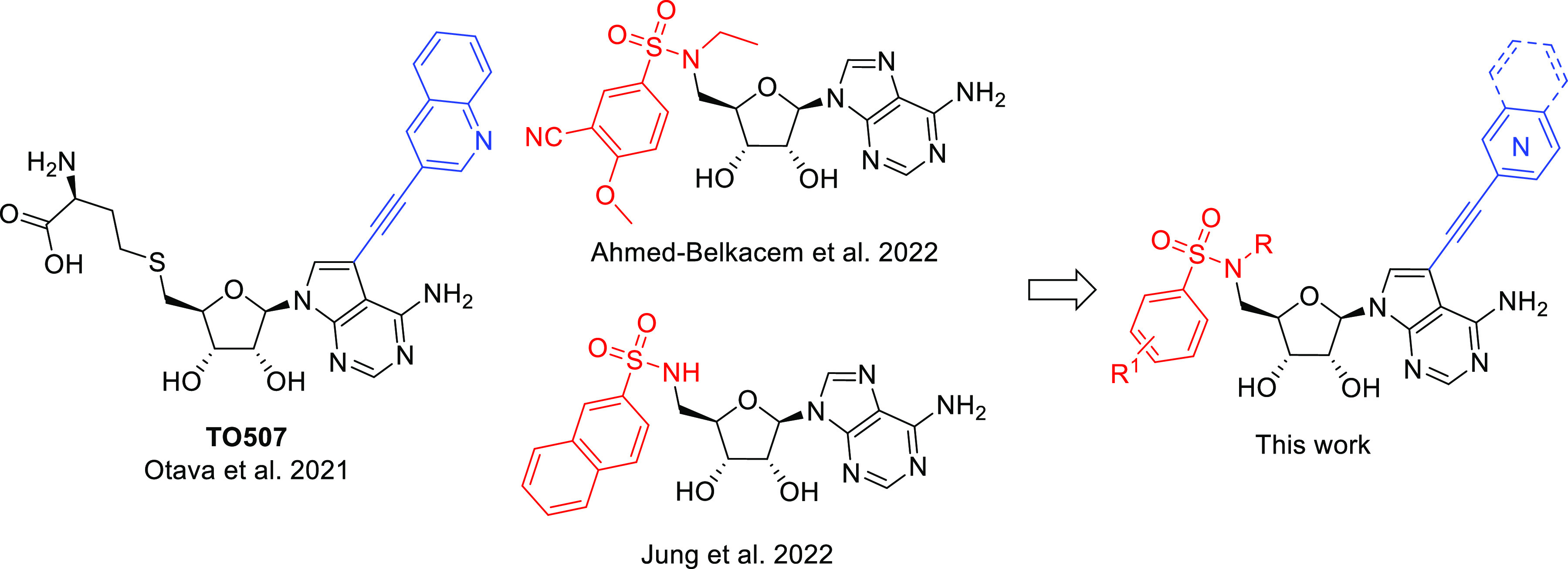
Design of nsp14 MTase inhibitors combining the strengths of both approaches to SAH analogue modification.

We based the design of our new compounds on a combination of our previous results^22^ that sought to exploit the lateral cavity in the SAM-binding site by placing a suitable substituent on the 7-deazapurine nucleobase of SAH derivatives, with the results of our colleagues,^17,18^ who focused on the preparation of bisubstrate inhibitors also derived from SAH lacking an amino acid residue and bearing variously substituted arylsulfonamides at the 5′ position of SAH (Figure 1). Our goal was to determine whether such a design could lead to a significant increase in activity while maintaining selectivity for human MTases.

**Scheme 1:**
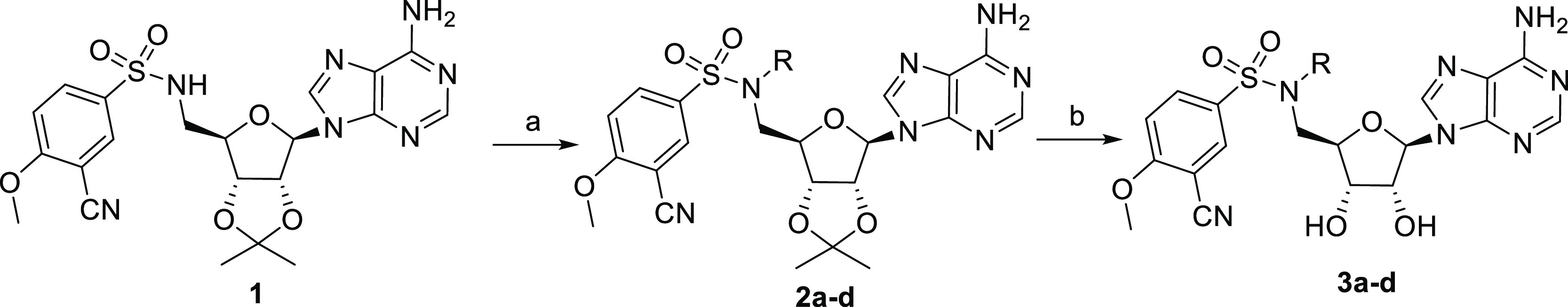
Preparation of the Purine-Based Derivatives **3a–d** Reagents and conditions: (a) RX, additive, K_2_CO_3_/Cs_2_CO_3_, DMF, 50 °C, 1–3 days; (b) 50% aqueous formic acid, RT, 1–3 days.

Before conducting the study on 7-substituted 7-deazapurine derivatives, we wanted to confirm the activity of the previously reported inhibitors (e.g., compounds **3a**) in our assay and see if they could be modified to be more effective. We prepared two small series of compounds, one modified on the phenyl substituent of the sulfonamide group (6 derivatives disubstituted at positions 3 and 4) and the other modified on the alkyl substituent. The first series of compounds did not perform better than the original, so we continued to use the 3-cyano-4-methoxyphenyl substituent in our subsequent studies (data not shown). This was not the case for the sulfonamide N-alkyl substituents (Scheme 1 and Table 1, R). In a simple series of methyl, ethyl, isopropyl, and cyclopentyl, both in our docking experiments and in the inhibitory activity assays, sulfonamide nitrogen decorated with a methyl (compound **3b**) showed higher activity than its ethyl counterpart (compound **3a**), and any larger group (compounds **3c** and **3d**) in this position led to significantly decreased activity (Table 2).

**Table 1:**
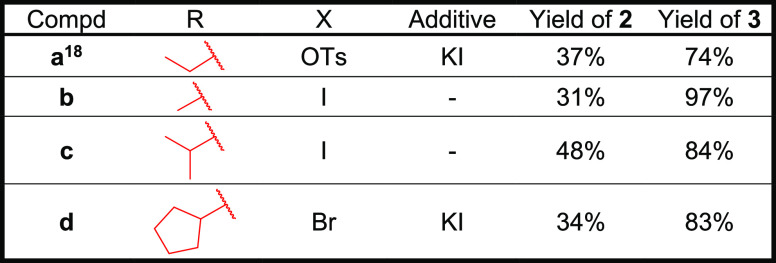
Reaction Conditions and Yields of Compounds **2** and **3** for Scheme 1

**Table 2:**
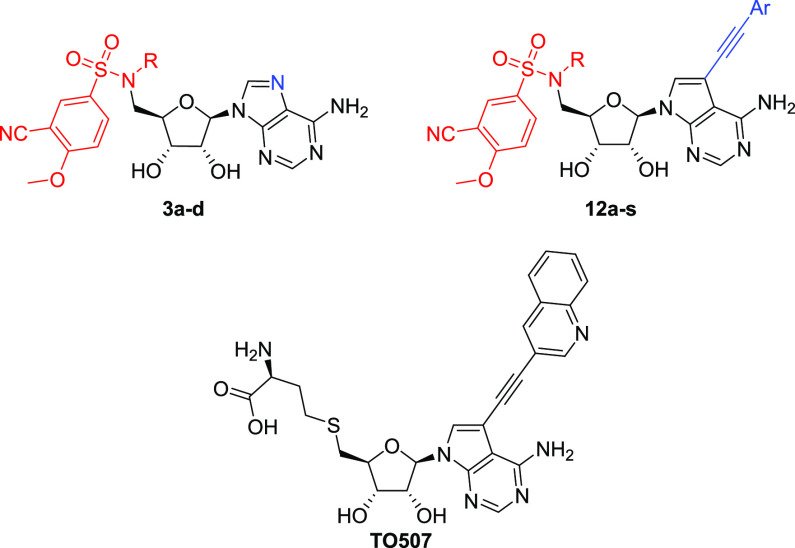

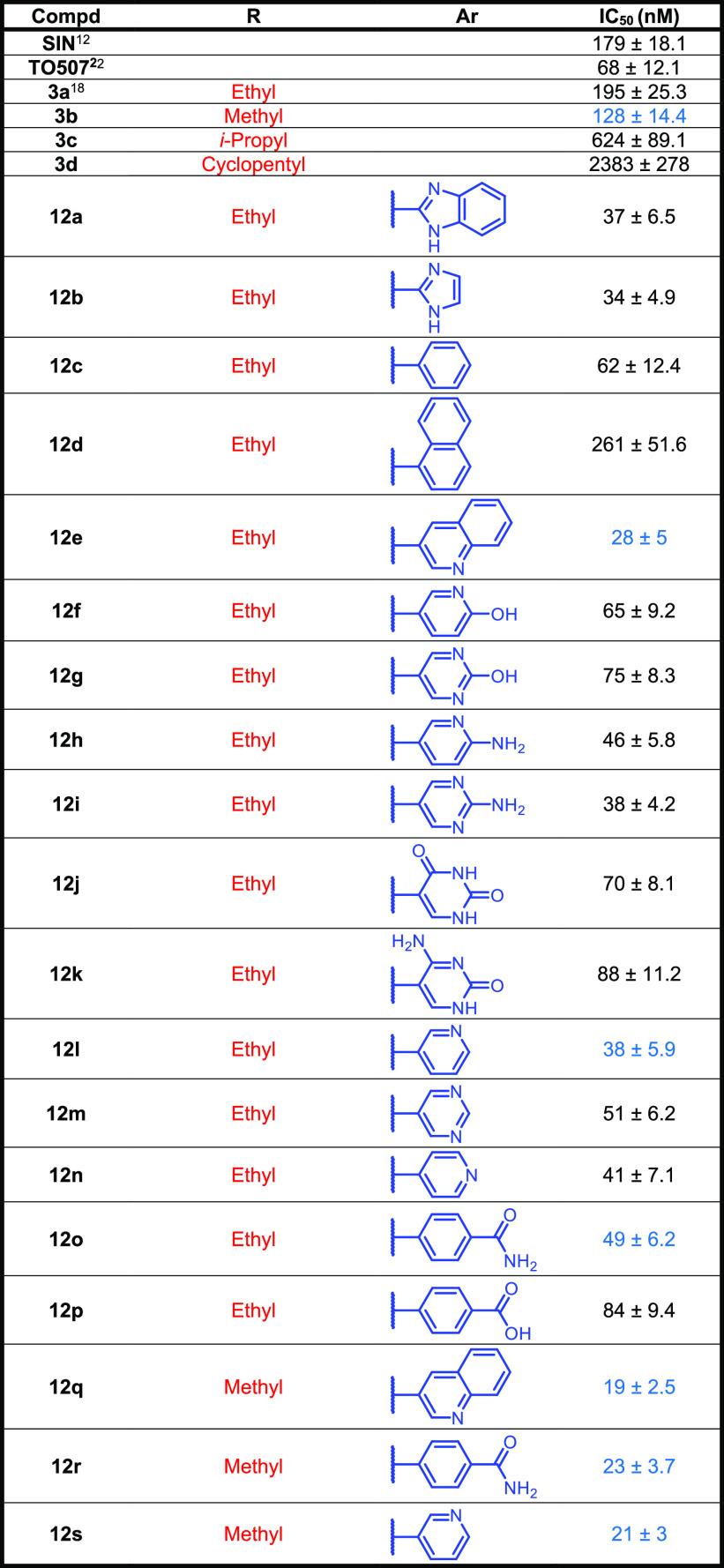
Inhibitory Activity of Novel Compounds Against SARS-CoV-2 nsp14 7-N MTase^a^

a For SIN—sinefungin—see ref (12); TO507—see ref (22), and **3a** see ref (18).

### Chemistry

We started the synthesis of compounds **3a–d** from a known protected nucleoside analogue **1**.^18^ Intermediates **2a–d** were prepared by alkylation by (pseudo)haloalkanes. In the case of less reactive leaving groups (OTs, Br), KI was used as an additive. Introduction of bulkier substituents in compounds **2c–d** also required the addition of Cs_2_CO_3_. Low to moderate yields of this step were caused mostly by nonselective alkylation. Removal of the isopropylidene protecting group was performed in 50% formic acid, providing final nucleosides **3a–d** in good to excellent yields (Table 1).^18^

**Scheme 2:**
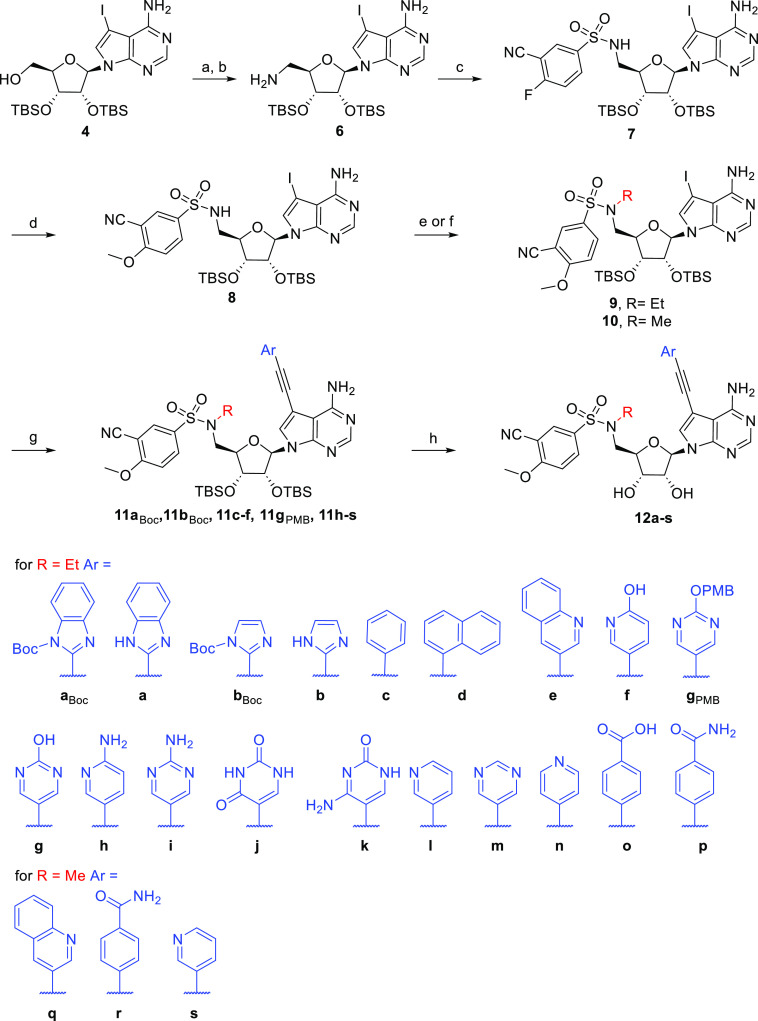
Preparation of the Main Series of Modified Nucleosides **12a–s** Reagents and conditions: (a) phthalimide, DIAD, Ph_3_P, THF, RT, ON, 92%; (b) N_2_H_2_·H_2_O, EtOH, 80 °C, 2 h, 80%; (c) 3-cyano-4-fluorobenzenesulfonyl chloride, TEA, DCM, RT, 30 min, 95%; (d) NaH, MeOH, 50 °C, 3 h, quant.; (e) EtOTs, Cs_2_CO_3_, DMF, 40 °C, 24 h, 83%, (f) (i) DMF-DMA, DMF, 60 °C, 2 h; (ii) MeI, Cs_2_CO_3_, DMF, RT, 90 min; (iii) 5 M NH_3_, MeOH, 50 °C, 3 d, 95% over three steps; (g) Pd cat., CuI, TEA, solvent, 50 °C, 1–16 h, see Supporting Information, Table S1; (h) TFA or TEA·3HF, see Supporting Information, Table S1.

Synthesis of the target molecules modified also on the nucleobase started from a 2′,3′-TBS-protected 7-deaza-7-iodoadenosine **4** (Scheme 2). Mitsunobu reaction with phthalimide and subsequent hydrazinolysis afforded 5′-amino-5′-deoxy nucleoside **6**.^22,28^ Coupling of **6** with commercial 3-cyano-4-fluorobenzenesulfonyl chloride was performed using a modified published procedure giving a 95% yield. The fluorine atom was then substituted using sodium methoxide in a nearly quantitative yield, furnishing the key intermediate **8**.^18^

Since sulfonamide alkylation proved to be troublesome in our pilot experiments, modified methodology was used for alkylation of **8**. Combination of EtOTs and Cs_2_CO_3_ in DMF afforded compound **9** in an 83% yield, which is a significant improvement compared to the preparation of **2a** (37%). Previously observed overalkylation when using MeI was prevented by introducing formamidine protection prior to alkylation. Subsequent deprotection in 5 M methanolic ammonia gave **10** in a 95% yield over the three steps.^29^

Alkylated sulfonamides **9** and **10** were then subjected to Sonogashira cross-coupling reaction with various heteroaryl acetylenes providing intermediates **11a**_**Boc**_, **11b**_**Boc**_, **11c–f**, **11g**_**PMB**_, and **11h–s** which were finally deprotected using either TFA/water mixture or TEA·3HF affording compounds **12a–s** (Supporting Information Table S1).^22,30^

### Inhibition of SARS-CoV-2 nsp14 MTase

**Figure 2:**
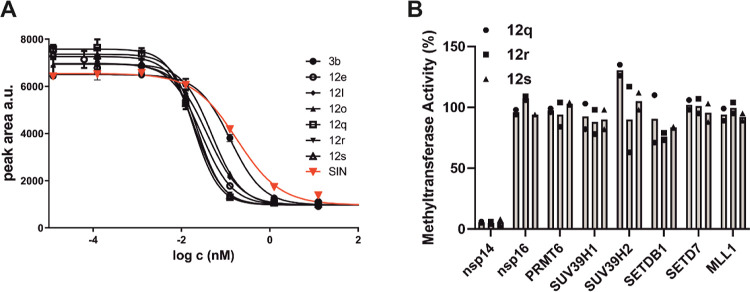
(A) Concentration-dependent nsp14 MTase inhibition by selected compounds assessed using Echo MS assay. The IC_50_ values are presented in Table 2. (B) Selectivity of nsp14 inhibitors. Selectivity of **12q**, **12r**, and **12s** against the SARS-CoV-2 nsp10-nsp16 complex, and six human methyltransferases were tested at 1 μM by SPA assay as described in the Supporting Information. Experiments were performed in duplicate.

All final compounds were tested in our assay using an Echo MS system coupled with a Sciex 6500 triple-quadrupole mass spectrometer in an arrangement similar to that previously described by Pearson et al.^27^ This system allowed rapid testing of the whole series of new derivatives and provided a very consistent set of intercomparable IC_50_ values within a single run. We also included three reference inhibitors—sinefungin (**SIN**), the best compound from our original study (**TO507**), and the best compound obtained among the sulfonamide bisubstrate inhibitors (**3a**). All tested compounds showed reliable concentration dependence (selected examples are shown in Figure 2A).

In our preliminary series, we focused on purine derivatives bearing alkyl substituents on the nitrogen of the sulfonamide group. As already suggested by our docking studies, methyl derivative **3b** (Table 2, R = Me) was more potent than the original ethyl counterpart **3a** (Table 2, R = Et) identified by Ahmed-Belkacem et al.^18^ as their best derivative. This discovery proved to be invaluable for our study, even though at that time we had already in our hands a number of derivatives with the ethyl substituent (Table 2, **12a–12p**). For the consistency and comparability of our results, we continued with the ethyl series and prepared the respective methyl analogues with only a few arylethynylene substituents (Table 2, **12q–12s**).

In our main series, we focused on derivatives bearing unsubstituted or substituted phenyl, imidazole, pyridine, and pyrimidine substituents connected to position 7 of the 7-deazapurine ring with an ethynylene linker (Table 2, **12a–12s**). Based on this table, we can draw conclusions on SAR trends. Derivatives with small unsubstituted heterocycles exhibited inhibitory activity ranging from 34 to 51 nM and outperformed the plain phenyl derivative **12c**. An additional ring’s annulation effect depended on its direction, and the 3-quinolinyl derivative **12e** showed the highest inhibitory activity. Among substituted pyridines and pyrimidines, only the derivatives with an amino group in the para position retained activity, and the carboxamide functional group proved beneficial for the phenyl derivative. Derivatives with a methyl substituent had consistently superior activity to the ethyl series, and **12q**, **12r**, and **12s** were the strongest inhibitors of the study. Compounds **12q**, **12r**, and **12s** were also selective against SARS-CoV-2 nsp10-nsp16 complex and 6 human methyltransferases (Figure 2B).

### Cellular Uptake and Stability of Selected Compounds

**Figure 3:**
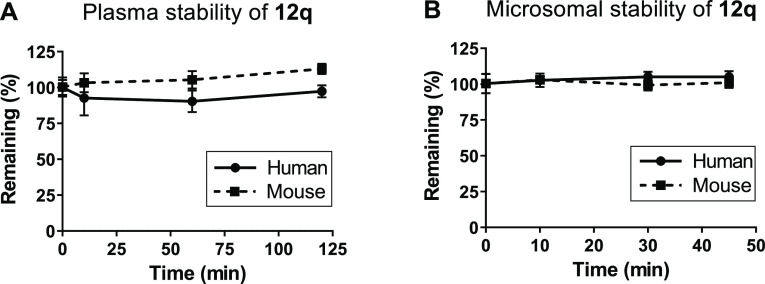
Metabolic stability of 12q in human and mouse plasma (A) and liver microsomes (B).

While an enzymatic MTase assay represents a valuable fast screening platform providing the information that can be directly used for SAR analyses, it is not fully predictive for the compounds’ activity in whole cells. In order to verify the ability of the compounds to reach the intracellular compartment, we measured the steady-state accumulation of representative compounds in intact CCRF-CEM, VERO-E6, and Calu-3 cells (Tables 3 and S2). The calculated ratios between total compound concentration in the cells and compound concentration in the medium (*K*_p_) were found to be very high for all compounds (ranging from hundreds to tens of thousands) and strongly indicate that the compounds cross cellular barriers easily and accumulate in the cells at mM concentrations—for comparison note that the reported intracellular uptake of tenofovir alafenamide in the same cell type is 0.9 μM for the prodrug and 67 μM for free tenofovir.^31^ Compound **12q** was almost completely cleared from the medium after 60 min incubation with the cells rendering it among the most permeable compounds of the series based on *K*_p_ values. It should be noted that the apparent intracellular concentration of the compounds may be biased by their potential metabolism. Therefore, we decided to subject the selected derivatives to in vitro stability studies. However, these studies clearly showed that all derivatives are stable in blood plasma and that derivatives **12q** and **12r** also show excellent stability in microsomes, both mouse and human (Figure 3 and Supporting Information Table S3).

**Table 3:** Intracellular Accumulation of Selected Compounds in CCRF-CEM, VERO-E6, and Calu-3 Cells (60 min Incubation, 50 μM Concentration at *t* = 0)^a^

compd	cell type	EC (μM) ± sd	IC (μM) ± sd	*K*_p_
**12q**	CCRF-CEM	0.6 ± 0.1	3140 ± 348	5325
**12r**		12 ± 6	10,708 ± 358	897
**12s**		15 ± 3	9249 ± 1390	604
**12q**	VERO-E6	0.1 ± 0.1	18,533 ± 7709	211 805
**12r**		1.1 ± 0.5	19,777 ± 7485	17 231
**12s**		2.6 ± 2.3	13,578 ± 3626	5208
**12q**	Calu-3	5.6 ± 5.7	89,640 ± 6514	16 015
**12r**		4.0 ± 0.2	5072 ± 974	1254
**12s**		14.7 ± 0.8	1557 ± 42	106

a EC—extracellular concentration in the media at *t* = 60 min; IC—intracellular concentration in the cells at *t* = 60 min; *K*_p_—intracellular accumulation ratio (IC/EC).

### Inhibition of SARS-CoV-2 In Vitro

The set of compounds showing potent inhibition of SARS-CoV-2 nsp14 MTase were further tested for their ability to inhibit SARS-CoV-2 replication in Vero E6 and Calu-3 cells. Here, two-fold serial dilutions of each compound starting from 100 μM concentration were added to the cells followed by the infection with SARS-CoV-2, and the inhibition of virus-induced cytopathic effect was determined 3 days post-infection by XTT assay. At the same time, the cytotoxicity of each compound was determined from identical dilutions in uninfected cells. The compounds did not exhibit any notable cytotoxicity or considerable inhibition of SARS-CoV-2 replication at concentrations up to 100 μM in both Vero E6 and Calu-3 cells (Supporting Information, Table S4). While the lack of inhibition of SARS-CoV-2 in Vero E6 cells can be explained by their infamous defective interferon response to viral infections,^32^ the lack of SARS-CoV-2 inhibition in Calu-3 cells is more confounding because the SARS-CoV-2 infection results in an activation of innate immune responses in these cells.^33^ Moreover, Calu-3 cells are easily infectible and support robust SARS-CoV-2 production. Thus, the inability of tested compounds to inhibit SARS-CoV-2 replication in Calu-3 cells, despite their high intracellular concentrations, remains an unanswered question that offers an interesting scientific space for subsequent studies in the field of virology and especially the host–virus interaction.

### Docking Study of nsp14 Inhibitors

To understand how our compounds bind in the active site of nsp14 MTase, we used the structure obtained by Czarna et al. (PDB ID: 7R2V)^33^ with SAH bound in the SAM-binding site. Throughout the project, we ran countless docking experiments using GOLD and Autodock Vina. GOLD^34^ and Autodock Vina^35^ are two widely used docking programs, and while both have been shown to perform well in various benchmark studies, Autodock Vina is generally considered to have superior scoring power, while GOLD has better sampling power.^36^

While this proved true for many of our projects, Autodock Vina clearly outperformed GOLD in this project. In general, we received the best scores for docking poses that placed the aryl ethynylene substituent in the cavity above the nucleobase, which in both softwares was relatively well aligned with the purine position of SAH, while also placing the aryl sulfonamide moiety in the RNA-binding cavity overlapping nicely with the guanine nucleobase (we used a cap-bound crystal structure reported by Imprachim et al.^24^ PDB ID: 7QIF). Although GOLD software was able to place the sulfonamide moiety into the RNA-binding site and generally worked better with the sugar part, it was often unable to correctly identify the proper conformation under standard setup. In contrast, both used versions of Autodock Vina excelled in the consistency of the position of the sulfonamide substituent for derivatives with differently modified nucleobases. Therefore, we believe that in this case, it is preferable for ordinary users to use the AutoDock Vina to score and rank individual derivatives.

**Figure 4:**
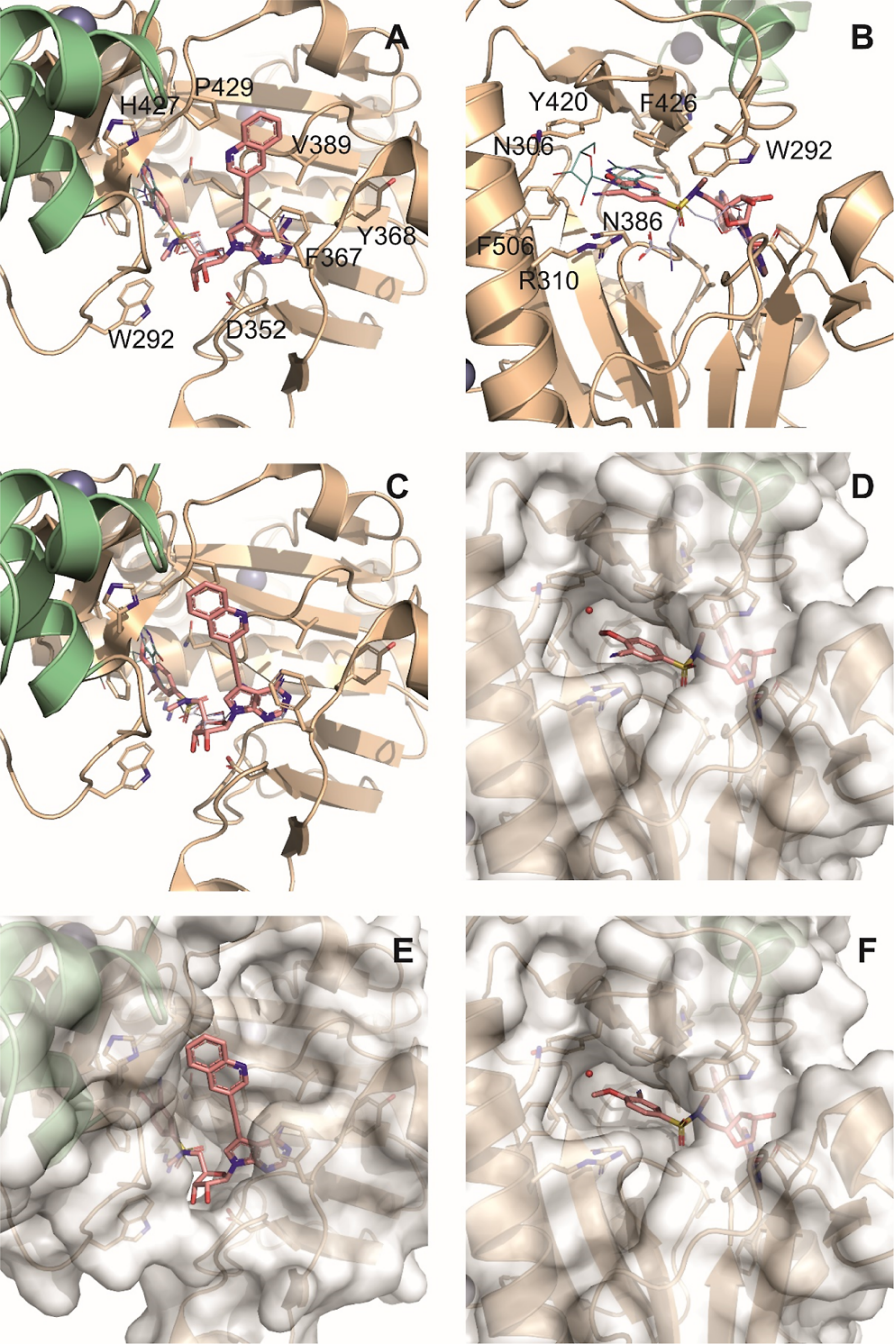
Comparison of docking experiments of compound **12q** performed by GOLD (A,B) and Autodock Vina (Poses A, C, and D; Poses B, E, and F). The ligand is shown in pink as sticks in all cases. We included SAH (blue white wires) and guanosine part of RNA cap (light teal wires) for better orientation in the SAM- and RNA-binding sites, respectively (A–C).

As an example, we have chosen our best derivative, **12q**, to demonstrate the differences in the docking poses of each substituent using these two types of software tools and show a potential future approach for improving these derivatives (Figure 4). GOLD was able to identify the pose shown in Figure 4A,B as the only one that placed the aryl sulfonamide moiety at the site where the guanine RNA cap is normally bound. This model nicely places the aryl ethynylene in the purine base region of the SAH which is shown as the blue–white wire (Figure 4A). It is clear that the nitrile group in this arrangement is directed toward the exit of the cavity, where it probably interacts with the protein *via* hydrogen bridges over water present in the cavity (Figures 4B and S1B,C). Autodock Vina also gave the highest score to a similar pose (Pose A), although the aryl ethynylene substituent is oriented differently and the aryl sulfonamide moiety is shifted toward the exit of the RNA cavity, in which the methoxy group is more likely to interact with the water molecule (Figures 4C,D and S1D–F). The position with the second highest score according to Autodock Vina (Pose B; Figures 4E,F and S1G–I) offers a very tempting alternative to these two models. The substituent on the nucleobase is in virtually the same position as in the previous Autodock Vina model (compare Figure S1D,G), but the aryl sulfonyl substituent is rotated 180° in the RNA-binding site, thus directing the nitrile group into the cavity (Figure 4F). Even though this position intuitively seems preferable, given that the match with the guanine base of the cap seems to be the most adequate and there is a possible interaction between the nitrile group and residue Thr428 or Asn 388, in almost all cases, it had a lower ranking in Autodock Vina than the pose previously mentioned (see detailed analysis of Autodock Vina results for all compounds from the main series in Supporting Information Table S5).

## Conclusions

Inhibiting viral RNA methylation is an intriguing new strategy in antiviral therapy. Nsp14 MTase is considered a suitable target for the therapy against COVID-19 and its causative agent SARS-CoV-2. Here, we have described the design and activity of novel inhibitors of this viral enzyme on a rational basis. We have shown their unprecedented activity on the isolated nsp14 enzyme with the most active inhibitor in the study, **12q** (**STM969**), exerting IC_50_ of 19 nM, making it one of the most active nsp14 inhibitors ever reported. We have also shown that these compounds are nontoxic in vitro and are not inhibiting any human MTases we have examined. They are also the first substances with such high inhibitory activity against SARS-CoV-2 nsp14 that are able to readily penetrate into cells. Poor inhibition of SARS-CoV-2 replication in vitro by these compounds suggests that the role of this enzyme is still unclear and requires further investigation as its involvement in viral replication in cell lines is not yet fully understood. These unique nsp14 inhibitors represent an invaluable tool for chemical biology and may help to explore the function of nsp14 and the entire capping machinery at the level of cells, organs, and even the whole organism.

## Supporting Information Available

Synthetic procedures, methods for MTase, antiviral and cytotoxicity assays, docking studies, and ^1^H and ^13^C NMR of final analogues (PDF)

## Abbreviations

SARS-CoV: severe acute respiratory syndrome-related coronavirus
COVID-19: coronavirus disease 2019
ExoN: 3′-to-5′ exoribonuclease
MTase: methyl-transferase
SAM: *S*-adenosyl-l-methionine
SAH: *S*-adenosyl-l-homocysteine
