# Supplementary material for: Rational Design of Highly Potent SARS-CoV-2 nsp14 Methyltransferase Inhibitors
Source: ACS Omega. 2023 Jul 21;8(30):27410–8. doi: 10.1021/acsomega.3c02815 (PMC10398685; doi:10.1021/acsomega.3c02815)
Supplement: Supplementary file 1 — ao3c02815_si_001.pdf [file ao3c02815_si_001.pdf]

Supporting Information

Rational design of highly potent SARS-CoV-2 nsp14 methyltransferase inhibitors

Milan Štefek,[1,2] Dominika Chalupská,[1] Karel Chalupský,[1] Michala Zgarbová,[1] Alexandra Dvořáková,[1] Petra Krafčíková,[1] Alice Shi Ming Li,[3] Michal Šála,[1] Milan Dejmek,[1] Tomáš Otava,[1] Ema Chaloupecká,[1] Jaroslav Kozák,[1] Ján Kozic,[1] Masoud Vedadi,[3,4] Jan Weber,[1] Helena Mertlíková-Kaiserová,[1] Radim Nencka[1,*]

[1]Institute of Organic Chemistry and Biochemistry of the Czech Academy of Sciences, Flemingovo náměstí 2, Prague 6, 166 10, Czech Republic

[2]Department of Organic Chemistry, Faculty of Science, Charles University, Prague 128 00, Czech Republic

[3] Department of Pharmacology and Toxicology, University of Toronto, Toronto, Ontario M5S 1A8, Canada

[4] QBI COVID-19 Research Group (QCRG), San Francisco, CA, USA

*To whom correspondence should be addressed:

Radim Nencka; Tel.: +420-220-183-265; E-mail: radim.nencka@uochb.cas.cz

Table of Contents

1. General information 3

2. Synthetic procedures 4

2.1 Preparation of (het)arylacetylenes 4

2.2 Preparation of the purine based derivatives 5

2.3 Preparation of double modified side analogues 7

2.4 Final deprotection 19

3. Biochemical assays 30

3.1. Nsp14 Echo MS assay 30

3.2. Scintillation proximity selectivity assays 30

3.3. Intracellular Uptake 31

3.4. Microsomal stability 31

3.5. Plasma stability 32

3.6. Caco-2 permeability assay 32

4. Antiviral and cytotoxicity assays 33
5. Docking studies 34
5.1. Docking in GOLD software 34
5.2. Docking in Autodock Vina 34
6. Supporting Tables 36
7. ^{1}H and ^{13}C NMR of final compounds 41
8. HPLC purity of final compounds 63
9. References 64

1. General information

Reagents were purchased from Sigma Aldrich and Fluorochem and used as received without any further purification or prepared according to published procedures.[1-12] CH_2Cl_2 was distilled from P_2O_5 and kept over 4Å molecular sieves. Thin layer chromatography (TLC) was performed on silica gel 60 F_{254} plates (Merck). Compounds were visualized on the TLC plates by irradiation with UV light. For normal flash column chromatography (VWR International Silica gel 60, particle size 0.040–0.063 mm) as well as for reverse-phase flash column chromatography (C18 RediSep Rf columns), a Combiflash® Rf from Teledyne ISCO was used. UPLC samples were measured on Waters UPLC H-Class Core System (column Waters Acquity UPLC BEH C18 1.7 µm, 2.1 mm × 100 mm), Waters Acquity UPLC PDA detector, mass spectrometer Waters SQ D2, and MassLynx mass spectrometry software. ^{1}H and ^{13}C NMR spectra for the reported compounds were recorded on a Bruker Avance III™ HD 400 instrument (400.0 MHz for ^{1}H and 101 MHz for ^{13}C) using inverse broadband probe with ATM module (5 mm BBO-1H Z-GRD), Bruker Avance III™ HD 400 instrument with broadband PRODIGY cryoprobe with ATM module (5 mm CPBBO BB-1H/19F/D Z-GRD) or JEOL JNM-ECZR 500 (5 mm FG/ROHFX probe). Chemical shifts (δ) and coupling constants (*J*) are expressed in ppm and Hz, respectively. The NMR experiments were performed in DMSO-d6 and referenced to the solvent signal (δ 2.50 for ^{1}H NMR and 39.70 for ^{13}C NMR). Shifts of ^{1}H and ^{13}C which were only observed in 2D spectra are marked with asterisk (*). High resolution mass spectrometry (HRMS) analyses were carried out on an LTQ XL Orbitrap XL (Thermo Fisher Scientific) using electrospray ionization (ESI). Purity of all final SAH analogues was determined by analytical HPLC using LCMS-2020 system from Shimadzu equipped by CORTECS column (C18 2.7 µm, 50 × 4.6 mm).

2. Synthetic procedures

2.1 Preparation of (het)arylacetylenes

2-Hydroxy-5-ethynylpyridine (A1)

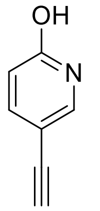

2-Hydroxy-5-iodopyridine (884 mg, 4.0 mmol), $Pd(PPh_3)_2Cl_2$ (113 mg, 0.16 mmol, 0.04 eq.) and CuI (60 mg, 0.32 mmol, 0.08 eq.) were suspended in anhydrous THF (40 mL). TEA (1.4 mL, 10.0 mmol, 2.5 eq.) was added and the resulting mixture was degassed by repeated evacuation-backfilling with argon. (Triisopropylsilyl)acetylene (2.2 mL, 10.0 mmol, 2.5 eq.) was finally added and the mixture was stirred at 55 °C overnight. The volatiles were evaporated and the residue was subjected to flash column chromatography (10–30% of EtOAc/EtOH (4:1) mixture in DCM). Intermediate **A1-I** (795 mg, 72%) was obtained as light brown solid. **^{1}H NMR** (400 MHz, DMSO-*d$_6$*) δ 11.88 (s, 1H), 7.62 (s, 1H), 7.40 (dd, *J* = 9.4, 2.1 Hz, 1H), 6.32 (d, *J* = 9.3 Hz, 1H), 1.07 (s, 21H). **^{13}C NMR** (101 MHz, DMSO-*d$_6$*) δ 161.2, 142.9, 140.4, 120.3, 103.7, 100.5, 89.5, 18.6, 10.9. **LRMS** (ESI) $[M + H]^+$ m/z: 276.1.

Compound **A1-I** was dissolved in THF (35 mL) and 1M TBAF in THF (3.2 mL, 3.16 mmol, 1.1 eq.) was added. After stirring at RT for 6 hours, the volatiles were evaporated. Flash column chromatography (2–10% of MeOH in DCM) afforded pure **A1** (330 mg, 97%). **^{1}H NMR** (400 MHz, DMSO-*d$_6$*) δ 11.87 (s, 1H), 7.66 (d, *J* = 2.5 Hz, 1H), 7.43 (dd, *J* = 9.5, 2.6 Hz, 1H), 6.36–6.28 (m, 1H), 4.04 (s, 1H). **^{13}C NMR** (101 MHz, DMSO-*d$_6$*) δ 161.3, 142.9, 140.4, 120.2, 99.8, 80.5, 80.2. **LRMS** (ESI) $[M + H]^+$ m/z: 120.0.

5-Ethynyl-2-((4-methoxybenzyl)oxy)pyrimidine (A2)

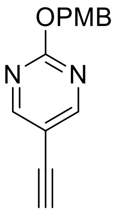

5-Bromo-2-hydroxypyrimidine (525 mg, 3.0 mmol) was suspended in anhydrous THF (30 mL) together with PPh_3 (1.42 g, 5.4 mmol, 1.8 eq.) and 4-methoxybenzyl alcohol (0.56 mL, 4.5 mmol, 1.5 eq.). DIAD (0.883 mL, 4.5 mmol, 1.5 eq.) was added to the stirred mixture and stirring was continued at RT overnight. Solvent was evaporated and the residue subjected to flash column chromatography (0–20% of EtOAc in cyclohexane) providing product **A2-I** (290 mg, 33%). ^{1}H and ^{13}C NMR spectroscopy was consistent with the published data.[13]

Above prepared intermediate **A2-I** (285 mg, 0.97 mmol), $Pd(PPh_3)_2Cl_2$ (27 mg, 0.039 mmol, 0.04 eq.) and CuI (15 mg, 0.08 mmol, 0.08 eq.) were suspended in anhydrous THF (10 mL). TEA (0.336 mL, 2.41 mmol, 2.5 eq.) was added and the resulting mixture was degassed by repeated evacuation-backfilling with argon. (Triisopropylsilyl)acetylene (0.542 mL, 2.41 mmol, 2.5 eq.) was finally added and the mixture was stirred at 55 °C for 90 minutes. The volatiles were evaporated and the residue was subjected to flash column chromatography (0–20% of EtOAc in cyclohexane). Coupling product **A2-II** (202 mg, 53%) was isolated as yellow oil. Unreacted starting material **A2-I** (131 mg, 46%) was isolated back. **^{1}H NMR** (400 MHz, DMSO-*d$_6$*) δ 8.74 (s, 2H), 7.44–7.35 (m, 2H), 6.98–6.90 (m, 2H), 5.34 (s, 2H), 3.75 (s, 3H), 1.10 (m, 21H). **^{13}C NMR** (101 MHz, DMSO-*d$_6$*) δ 163.2, 162.1, 159.4, 130.2, 128.2, 114.0, 112.4, 100.6, 95.6, 68.9, 55.3, 18.6, 10.8. **LRMS** (ESI) $[M + H]^+$ m/z: 397.1.

Title compound was prepared by reaction of **A2-II** (298 mg, 0.75 mmol) with TBAF (1M solution in THF, 0.90 mL, 0.90 mmol, 1.2 eq.) in anhydrous DCM (7.5 mL). After 1 hour at RT the starting material was consumed. Reaction mixture was diluted with DCM and washed

twice with water and once with brine. Organic fraction was dried over Na_2SO_4. Crude product was purified on flash column chromatography (5–20% of EtOAc in cyclohexane) providing title compound **A2** (163 mg, 90%). **^{1}H NMR** (400 MHz, DMSO-*d*$_6$) δ 8.76 (s, 2H), 7.44–7.35 (m, 2H), 6.98–6.90 (m, 2H), 5.33 (s, 2H), 4.51 (s, 1H), 3.75 (s, 3H). **^{13}C NMR** (101 MHz, DMSO-*d*$_6$) δ 163.6, 162.4, 159.6, 130.5, 128.4, 114.2, 111.9, 86.1, 77.7, 69.1, 55.5. **LRMS** (ESI) $[M + H]^+$ m/z: 241.1.

***tert*-Butyl 2-ethynyl-1*H*-imidazole-1-carboxylate (A3)**

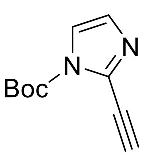

To a suspension of *tert*-butyl 2-bromo-1*H*-imidazole-1-carboxylate (2.52 g, 10.2 mmol), (triisopropylsilyl)acetylene (6.87 mL, 30.6 mmol, 3 eq.), CuI (437 mg, 2.30 mmol, 0.23 eq.) and $Pd(PPh_3)_2Cl_2$ (537 mg, 0.765 mmol, 0.075 eq.) in anhydrous THF (40 mL) was added triethylamine (4.30 mL, 30.9 mmol, 3 eq.) at RT and the mixture was stirred under argon atmosphere at 60 °C for 1 hour. The solution was diluted with EtOAc, washed with water and dried over Na_2SO_4. Solvent was evaporated and the residue subjected to flash column chromatography (0–10% of EtOAc in cyclohexane). The product was concentrated under vacuum. The residue (3.37 g, 9.67 mmol) was dissolved in THF (40 mL) and tetrabutylammonium fluoride (1M in THF, 14.5 mL, 1.5 eq.) was added. After stirring for 1 hour at 0 °C, the solvent was removed in vacuo. Flash column chromatography (0–45% of EtOAc in cyclohexane) afforded product **A3** (828 mg, 45% over two steps) as off-white solid. **^{1}H NMR** (400 MHz, DMSO-*d*$_6$) δ 7.62 (1H, s), 7.06 (1H, s), 4.62 (s, 1H), 1.58 (s, 9H). **^{13}C NMR** (101 MHz, DMSO-*d*$_6$) δ 146.3, 129.8, 120.1, 86.5, 84.8, 74.2, 27.5. **LRMS** (EI) $[M]^+$ m/z: 192.1.

2.2 Preparation of the purine based derivatives

***N*-(((3a*R*,4*R*,6*R*,6a*R*)-6-(6-Amino-9*H*-purin-9-yl)-2,2-dimethyltetrahydrofuro[3,4-*d*][1,3]dioxol-4-yl)methyl)-3-cyano-4-methoxybenzenesulfonamide (1) (See ref. [14])**

***N*-(((3a*R*,4*R*,6*R*,6a*R*)-6-(6-Amino-9*H*-purin-9-yl)-2,2-dimethyltetrahydrofuro[3,4-*d*][1,3]dioxol-4-yl)methyl)-3-cyano-*N*-ethyl-4-methoxybenzenesulfonamide (2a) (See ref. [14])**

***N*-(((3a*R*,4*R*,6*R*,6a*R*)-6-(6-Amino-9*H*-purin-9-yl)-2,2-dimethyltetrahydrofuro[3,4-*d*][1,3]dioxol-4-yl)methyl)-3-cyano-4-methoxy-*N*-methylbenzenesulfonamide (2b)**

To the solution of sulfonamide **1** (80 mg, 0.16 mmol) in anhydrous DMF (1.6 mL) was added K_2CO_3 (66 mg, 0.48 mmol, 3 eq.) followed by iodomethane (12 µl, 0.19 mmol, 1.2 eq.). Resulting mixture was stirred at RT for 14 hours. Since reaction proceeded very slowly, stirring was continued at 50 °C for 12 hours. After this time, further K_2CO_3 (22 mg, 0.06 mmol, 1 eq) and iodomethane (12 µl, 0.19 mmol, 1.2 eq.) were added and stirring was continued overnight. Reaction mixture was diluted with EtOAc and washed with water. Water layer was extracted once with EtOAc and combined organic fractions were washed with brine and dried over Na_2SO_4. Crude product was purified by flash column chromatography (2–10% of MeOH in DCM) followed by reverse phase chromatography (30–50% of ACN in water). This way product **2b** (26 mg, 32%) was obtained. **^{1}H NMR** (500 MHz, DMSO-*d*$_6$) δ 8.32 (s, 1H), 8.17 (s, 1H), 8.08 (d, *J* = 2.4 Hz, 1H), 7.90 (dd, *J* = 9.0, 2.4 Hz, 1H), 7.39–7.29 (m, 3H), 6.19 (d, *J* = 2.4 Hz, 1H), 5.47 (dd, *J* = 6.3, 2.4 Hz, 1H), 5.06 (dd, *J* = 6.3, 3.3 Hz, 1H), 4.29 (ddd, *J* = 7.5, 5.7, 3.3 Hz, 1H), 3.41 (dd, *J* = 14.2, 5.7 Hz, 1H), 3.15 (dd, *J* = 14.2, 7.5 Hz, 1H), 2.61 (s, 3H), 1.54 (s, 3H), 1.33 (s, 3H). **^{13}C NMR** (126 MHz, DMSO-*d*$_6$) δ 164.2, 156.7, 153.3, 149.2, 140.7,

134.6, 133.5, 129.9, 119.7, 115.5, 114.0, 113.6, 101.9, 89.5, 84.8, 83.6, 82.4, 57.7, 51.8, 36.5, 27.5, 25.7. **LRMS** (ESI) $[M + H]^+$ m/z: 516.1.

***N*-(((3a*R*,4*R*,6R,6a*R*)-6-(6-Amino-9*H*-purin-9-yl)-2,2-dimethyltetrahydrofuro[3,4-*d*][1,3]dioxol-4-yl)methyl)-3-cyano-*N*-isopropyl-4-methoxybenzenesulfonamide (2c)**

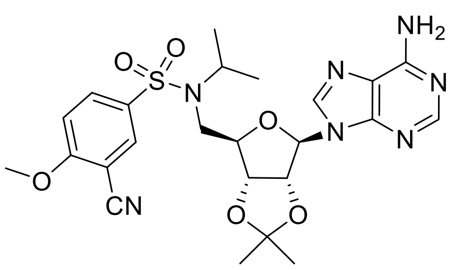

To the solution of starting material **1** (80 mg, 0.16 mmol) in anhydrous DMF (1.6 mL) was added K_2CO_3 (66 mg, 0.48 mmol, 3 eq.) followed by 2-iodopropane (19 µl, 0.19 mmol, 1.2 eq.). Resulting mixture was stirred at RT for 14 hours. Since reaction proceeded slowly, stirring was continued at 50 °C for 12 hours. After this time Cs_2CO_3 (50 mg, 0.19 mmol, 1.2 eq.) and 2-iodopropane (19 µl, 0.19 mmol, 1.2 eq.) were further added and reaction was stirred at the same temperature overnight. Reaction mixture was diluted with EtOAc and washed with water. Water layer was extracted once with EtOAc and combined organic fractions were washed with brine and dried over Na_2SO_4. Crude product was purified by reverse phase chromatography (30–50% of ACN in water, 0.1% of FA as modifier) furnishing compound **2c** (42 mg, 48%). **^{1}H NMR** (400 MHz, DMSO-*d₆*) δ 8.3 (s, 1H), 8.2 (d, *J* = 2.4 Hz, 1H), 8.2 (s, 1H), 8.0 (dd, *J* = 9.0, 2.4 Hz, 1H), 7.4 (m, 3H), 6.2 (d, *J* = 2.1 Hz, 1H), 5.5 (dd, *J* = 6.3, 2.1 Hz, 1H), 5.1 (dd, *J* = 6.3, 3.0 Hz, 1H), 4.5 (ddd, *J* = 8.0, 5.2, 3.1 Hz, 1H), 4.0 (s, 3H), 3.8 (hept, *J* = 6.6 Hz, 1H), 3.6 (dd, *J* = 15.4, 5.2 Hz, 1H), 3.2 (dd, *J* = 15.4, 7.9 Hz, 1H), 1.5 (s, 3H), 1.3 (s, 3H), 0.7 (d, *J* = 6.7 Hz, 3H), 0.7 (d, *J* = 6.7 Hz, 3H). **^{13}C NMR** (101 MHz, DMSO-*d₆*) δ 164.0, 156.7, 153.1, 149.0, 141.0, 134.4, 133.3, 132.7, 119.8, 115.5, 113.7, 113.5, 101.7, 89.9, 87.0, 83.8, 82.8, 57.6, 49.9, 45.6, 27.4, 25.6, 21.4, 19.9. **LRMS** (ESI) $[M + H]^+$ m/z: 544.1.

***N*-(((3a*R*,4*R*,6*R*,6a*R*)-6-(6-Amino-9*H*-purin-9-yl)-2,2-dimethyltetrahydrofuro[3,4-*d*][1,3]dioxol-4-yl)methyl)-3-cyano-*N*-cyclopentyl-4-methoxybenzenesulfonamide (2d)**

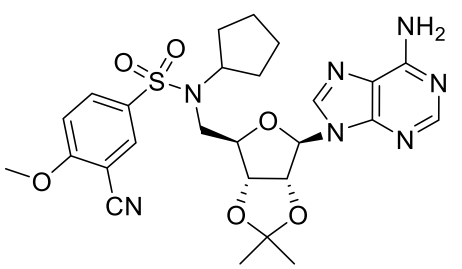

Sulfonamide **1** (80 mg, 0.16 mmol), K_2CO_3 (66 mg, 0.48 mmol, 3 eq.) and KI (3 mg, 0.02 mmol, 0.1 eq.) were suspended in anhydrous DMF (1.6 mL). To the resulting mixture bromocyclopentane (21 µl, 0.19 mmol, 1.2 eq.) was added and the reaction mixture was stirred at 50 °C for 24 hours. K_2CO_3 (26 mg, 0.19 mmol, 1.2 eq) and bromocyclopentane (21 µl, 0.19 mmol, 1.2 eq.) were added and stirring was continued for 24 hours at the same temperature. After this time Cs_2CO_3 (125 mg, 0.38 mmol, 2.4 eq) and bromocyclopentane (26 µl, 0.24 mmol, 1.5 eq.) were further added and stirring was continued overnight. Reaction mixture was diluted with EtOAc and washed with water. Water layer was extracted once with EtOAc and combined organic fractions were washed with brine and dried over Na_2SO_4. Crude product was purified by reverse phase chromatography (30–70% of ACN in water, 0.1% of FA as modifier) giving product **2d** (42 mg, 48%). **^{1}H NMR** (500 MHz, DMSO-*d₆*) δ 8.33 (s, 1H), 8.22 (d, *J* = 2.4 Hz, 1H), 8.14 (s, 1H), 8.06 (dd, *J* = 9.0, 2.4 Hz, 1H), 7.38 (d, *J* = 9.1 Hz, 1H), 7.36 (s, 2H), 6.26 (d, *J* = 1.9 Hz, 1H), 5.53 (dd, *J* = 6.3, 1.9 Hz, 1H), 5.15 (dd, *J* = 6.2, 2.8 Hz, 1H), 4.52 (dt, *J* = 7.0, 3.7 Hz, 1H), 4.00 (s, 2H), 3.91 (p, *J* = 8.5 Hz, 1H), 3.54 (dd, *J* = 15.5, 4.1 Hz, 1H), 3.09 (dd, *J* = 15.5, 8.8 Hz, 1H), 1.55 (s, 3H), 1.35 (s, 3H), 1.36–1.25 (m, 1H), 1.22–1.12 (m, 2H), 1.11–0.93 (m, 2H), 0.94–0.85 (m, 1H), 0.86–0.78 (m, 1H), 0.73 (dq, *J* =

12.5, 9.3 Hz, 1H). **^{13}C NMR** (126 MHz, DMSO-d_6) δ 164.2, 156.8, 153.2, 149.0, 141.2, 134.7, 133.6, 132.2, 119.9, 115.6, 113.6, 113.6, 101.8, 90.2, 87.8, 83.8, 83.0, 59.2, 57.7, 46.9, 29.6, 27.5, 27.4, 25.7, 23.5, 22.9. **LRMS** (ESI) $[M + H]^+$ m/z: 570.2.

2.3 Preparation of double modified side analogues

2-(((2*R*,3*R*,4*R*,5*R*)-5-(4-Amino-5-iodo-7*H*-pyrrolo[2,3-*d*]pyrimidin-7-yl)-3,4-bis((*tert*-butyldimethylsilyl)oxy)tetrahydrofuran-2-yl)methyl)isoindoline-1,3-dione (5)

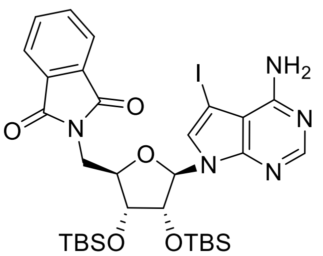

2′,3′-Silyl protected nucleoside[8] **4** (3.178 g, 5.120 mmol) (co-evaporated with toluene (50 mL)), phthalimide (0.829 g, 5.63 mmol, 1.1 eq.) and PPh_3 (1.477 g, 5.63 mmol, 1.1 eq.) were dissolved in anhydrous THF (50 mL). To the stirred mixture DIAD (1.11 mL, 5.63 mmol, 1.1 eq.) was slowly added at RT. After stirring under Ar at RT overnight, the volatiles were evaporated and the residue was subjected to flash column chromatography (5–20% of EtOAc/EtOH (4:1) mixture in DCM) giving product **5** (3.52 g, 92%). **^{1}H NMR** (400 MHz, DMSO-d_6) δ 8.03 (s, 1H), 7.95–7.89 (m, 2H), 7.89–7.83 (m, 2H), 7.79 (s, 1H), 6.66 (s, 2H), 6.07 (d, J = 7.7 Hz, 1H), 4.85 (dd, J = 7.6, 4.5 Hz, 1H), 4.23 (d, J = 4.5 Hz, 1H), 4.14–4.03 (m, 2H), 3.96–3.84 (m, 1H), 0.83 (s, 9H), 0.65 (s, 9H), 0.03 (s, 3H), -0.01 (s, 3H), -0.09 (s, 3H), -0.40 (s, 3H). **^{13}C NMR** (101 MHz, DMSO-d_6) δ 168.1, 161.7, 157.3, 152.1, 150.8, 134.9, 131.5, 127.7, 123.4, 103.5, 86.0, 82.6, 74.0, 73.5, 52.4, 25.7, 25.6, 17.9, 17.6, -4.6, -4.6, -5.0, -5.6. **LRMS** (ESI) $[M + H]^+$ m/z: 750.2.

7-((2*R*,3*R*,4*R*,5*R*)-5-(Aminomethyl)-3,4-bis((*tert*-butyldimethylsilyl)oxy)tetrahydrofuran-2-yl)-5-iodo-7*H*-pyrrolo[2,3-*d*]pyrimidin-4-amine (6)

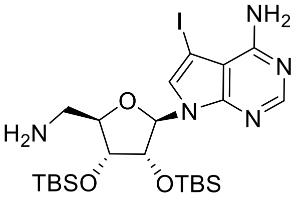

Compound **5** (3.51 g, 4.68 mmol) was suspended in EtOH (47 mL) and hydrazine hydrate (1.17 g, 23.4 mmol, 5 eq.) was added thereto. Resulting mixture was stirred at 80 °C for 2 hours, during which time the substrate was consumed. Solvent was evaporated and the residue was subjected to flash column chromatography (silica gel pre-treated with 15% of MeOH/NH_4OH (conc.) (9:1) mixture in DCM, 5–15% of MeOH/NH_4OH (conc.) (9:1) mixture in DCM) giving 5′-amino-5′-deoxyadenine analogue **6** (2.76 g, 95%) as white solid. **^{1}H NMR** (400 MHz, DMSO-d_6) δ 8.10 (s, 1H), 7.72 (s, 1H), 6.66 (s, 2H), 6.05 (d, J = 7.2 Hz, 1H), 4.65 (dd, J = 7.1, 4.7 Hz, 1H), 4.23 (dd, J = 4.6, 1.4 Hz, 1H), 3.87–3.79 (m, 1H), 2.83 (dd, J = 13.4, 5.5 Hz, 1H), 2.76 (dd, J = 13.5, 5.1 Hz, 1H), 0.92 (s, 9H), 0.68 (s, 9H), 0.13 (s, 3H), 0.11 (s, 3H), -0.11 (s, 3H), -0.41 (s, 3H). **^{13}C NMR** (101 MHz, DMSO-d_6) δ 157.3, 152.1, 150.7, 127.6, 103.5, 87.3, 86.2, 74.8, 73.1, 52.2, 43.8, 25.9, 25.6, 17.9, 17.6, -4.4, -4.5, -4.6, -5.4. **LRMS** (ESI) $[M + H]^+$ m/z: 620.2.

***N*-(((2*R*,3*R*,4*R*,5*R*)-5-(4-Amino-5-iodo-7*H*-pyrrolo[2,3-*d*]pyrimidin-7-yl)-3,4-bis((*tert*-butyldimethylsilyl)oxy)tetrahydrofuran-2-yl)methyl)-3-cyano-4-fluorobenzenesulfonamide (7)**

Amine **6** (1.115 g, 1.80 mmol) and 3-cyano-4-fluorobenzenesulfonyl chloride (435 mg, 1.98 mmol, 1.1 eq.) were placed into flask and dissolved in anhydrous DCM (45 mL). TEA (500 µl,

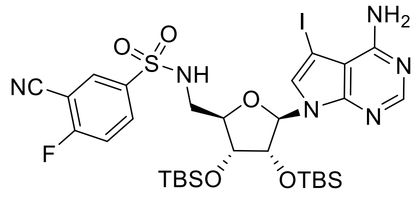

3.6 mmol, 2 eq.) was added to the stirred mixture and stirring was continued at RT for 30 minutes under argon. Volatiles were evaporated and the residue was subjected to flash column chromatography (10–20% of EtOAc/EtOH (4:1) mixture in cyclohexane) yielding product **7** (1.38 g, 95%) as white solid. **^{1}H NMR** (400 MHz, DMSO-*d$_6$*) δ 8.51 (t, *J* = 5.8 Hz, 1H), 8.35 (dd, *J* = 5.9, 2.4 Hz, 1H), 8.18 (ddd, *J* = 8.9, 5.0, 2.4 Hz, 1H), 8.09 (s, 1H), 7.73 (t, *J* = 9.0 Hz, 1H), 7.67 (s, 1H), 6.70 (s, 2H), 5.99 (d, *J* = 7.4 Hz, 1H), 4.66 (dd, *J* = 7.4, 4.7 Hz, 1H), 4.20–4.14 (m, 1H), 3.93–3.85 (m, 1H), 3.28–3.15 (m, 2H), 0.90 (s, 9H), 0.65 (s, 9H), 0.11 (s, 3H), 0.09 (s, 3H), -0.14 (s, 3H), -0.44 (s, 3H). **^{13}C NMR** (101 MHz, DMSO-*d$_6$*) δ 164.4 (d, *J* = 261.9 Hz), 157.4, 152.1, 150.3, 138.5 (d, *J* = 3.7 Hz), 134.4 (d, *J* = 10.3 Hz), 132.7, 128.0, 118.1 (d, *J* = 21.3 Hz), 113.0, 103.7, 101.6 (d, *J* = 16.5 Hz), 86.8, 84.3, 73.8, 73.1, 52.3, 44.7, 26.5, 25.9, 25.6, 17.9, 17.6, -4.51, -4.53, -4.6, -5.5. **^{19}F NMR** (377 MHz, DMSO-*d$_6$*) δ -102.35–102.47 **LRMS** (ESI) [M + H]$^+$ m/z: 803.1.

N-(((2*R*,3*R*,4*R*,5*R*)-5-(4-Amino-5-iodo-7*H*-pyrrolo[2,3-*d*]pyrimidin-7-yl)-3,4-bis((*tert*-butyldimethylsilyl)oxy)tetrahydrofuran-2-yl)methyl)-3-cyano-4-methoxybenzenesulfonamide (8)

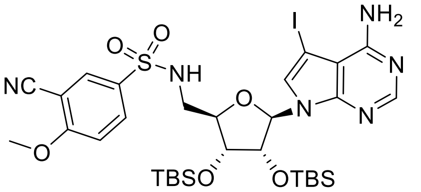

Sulfonamide **10** (1.09 g, 1.35 mmol) was dissolved in anhydrous methanol (30 mL) and NaH (60% in mineral oil, 270 mg, 6.76 mmol, 5 eq.) was carefully added to the stirred solution in small portions at RT. After the gas evolution ceased, the flask was closed with a septum equipped with a balloon filled with argon. Reaction was stirred at 50 °C for 3 hours. After cooling to ambient temperature methanol was evaporated and the residue dissolved in EtOAc, transferred into separatory funnel and washed with saturated NH_4Cl solution. Water layer was separated and extracted with another portion of EtOAc. Combined organic fractions were washed with brine, dried over Na_2SO_4, filtered and evaporated. The residue was subjected to flash column chromatography (2–10% of MeOH in DCM) affording **8** (1.10 g, quant.) as white solid. **^{1}H NMR** (400 MHz, DMSO-*d$_6$*) δ 8.33 (t, *J* = 5.6 Hz, 1H), 8.12 (d, *J* = 2.3 Hz, 1H), 8.09 (s, 1H), 8.06 (dd, *J* = 9.0, 2.4 Hz, 1H), 7.69 (s, 1H), 7.44 (d, *J* = 9.0 Hz, 1H), 6.71 (s, 2H), 5.99 (d, *J* = 7.5 Hz, 1H), 4.66 (dd, *J* = 7.5, 4.7 Hz, 1H), 4.17–4.13 (m, 1H), 4.00 (s, 3H), 3.92–3.85 (m, 1H), 3.21 (dt, *J* = 13.4, 5.1 Hz, 1H), 3.11 (dt, *J* = 13.6, 6.0 Hz, 1H), 0.90 (s, 9H), 0.65 (s, 9H), 0.10 (s, 3H), 0.09 (s, 3H), -0.15 (s, 3H), -0.45 (s, 3H). **^{13}C NMR** (101 MHz, DMSO-*d$_6$*) δ 163.5, 157.4, 152.0, 150.3, 133.6, 133.5, 132.3, 128.1, 115.3, 113.3, 103.7, 101.2, 86.8, 84.4, 73.8, 73.1, 57.3, 52.2, 44.7, 25.9, 25.6, 17.9, 17.6, -4.5, -4.5, -4.6, -5.6. **LRMS** (ESI) [M + H]$^+$ m/z: 815.1.

N-(((2*R*,3*R*,4*R*,5*R*)-5-(4-Amino-5-iodo-7*H*-pyrrolo[2,3-*d*]pyrimidin-7-yl)-3,4-bis((*tert*-butyldimethylsilyl)oxy)tetrahydrofuran-2-yl)methyl)-3-cyano-*N*-ethyl-4-methoxybenzenesulfonamide (9)

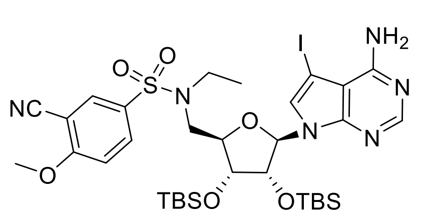

Compound **8** (1.15 g, 1.41 mmol), Cs_2CO_3 (552 mg, 1.69 mmol, 1.2 eq.) and ethyl tosylate (367 mg, 1. 84 mmol, 1.3 eq.) were dissolved in anhydrous DMF (18 mL). After stirring for 10 hours at 40 °C further Cs_2CO_3 (92 mg, 0.2 eq.) and ethyl tosylate (56 mg, 0.2 eq.) were added and the

stirring was continued at the same temperature for 12 hours. Solvent volume was reduced *in vacuo* and the residue was transferred into separatory funnel by dissolving in EtOAc and water. The layers were separated and water fraction was extracted with EtOAc twice. Combined organic fractions were washed with brine and dried over Na_2SO_4, filtered and evaporated. Flash column chromatography of the residue (5-20% of EtOAc/EtOH (4:1) mixture in DCM) provided **9** (1.24 g, 83%) as clear oil. **^{1}H NMR** (400 MHz, DMSO-*d$_6$*) δ 8.18 (d, *J* = 2.4 Hz, 1H), 8.10 (s, 1H), 8.08 (dd, *J* = 9.0, 2.4 Hz, 1H), 7.76 (s, 1H), 7.42 (d, *J* = 9.1 Hz, 1H), 6.68 (s, 2H), 6.05 (d, *J* = 7.5 Hz, 1H), 4.81 (dd, *J* = 7.4, 4.4 Hz, 1H), 4.30 (d, *J* = 4.3 Hz, 1H), 4.06–3.98 (m, 4H), 3.73 (dd, *J* = 15.1, 5.8 Hz, 1H), 3.36 (dd, *J* = 15.0, 7.8 Hz, 1H), 3.21 (q, *J* = 7.0 Hz, 2H), 0.97 (t, J = 7.1, 3H), 0.92 (s, 9H), 0.67 (s, 9H), 0.14 (s, 3H), 0.12 (s, 3H), -0.11 (s, 3H), -0.41 (s, 3H). **^{13}C NMR** (101 MHz, DMSO-*d$_6$*) δ 164.1, 157.7, 152.4, 151.0, 134.4, 133.1, 132.6, 128.3, 115.5, 113.6, 103.9, 101.7, 87.1, 84.6, 73.8, 73.7, 57.7, 52.7, 49.8, 44.3, 26.2, 25.9, 18.2, 17.9, 14.0, -4.2, -4.3 (2C)*, -5.1. **LRMS** (ESI) $[M + H]^+$ m/z: 843.2.

***N*-(((2*R*,3*R*,4*R*,5*R*)-5-(4-Amino-5-iodo-7*H*-pyrrolo[2,3-*d*]pyrimidin-7-yl)-3,4-bis((*tert*-butyldimethylsilyl)oxy)tetrahydrofuran-2-yl)methyl)-3-cyano-4-methoxy-*N*-methylbenzenesulfonamide (10)**

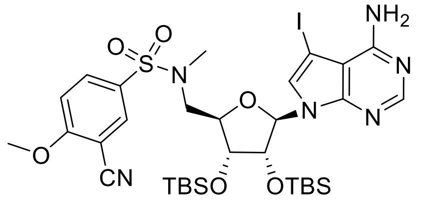

To the stirred solution of sulfonamide **8** (1.11 g, 1.37 mmol) in anhydrous DMF (14 mL) was added DMF-DMA (309 µl, 2.32 mmol, 1.7 eq.) at room temperature. Resulting mixture was stirred under argon at 60 °C for 90 minutes before adding further DMF-DMA (91 µl, 0.68 mmol, 0.5 eq.) and stirring for 40 minutes at the same temperature. Volatiles were evaporated leaving oily residue, which was dissolved in anhydrous DMF (14 mL). Cs_2CO_3 (0.534 g, 1.64 mmol, 1.2 eq.) was added to the solution at RT followed by iodomethane (111 µl, 1.78 mmol, 1.3 eq.) and the reaction was stirred at RT for 90 minutes. Volatiles were evaporated, the residue was dissolved in DCM and filtered through the pad of celite. The solvent was evaporated and the residue was dissolved in 5M ammonia in MeOH (30 mL) and transferred into pressure vessel. After stirring at 50 °C for 3 days the solvent was evaporated and the residue was subjected to flash column chromatography (2–10% of MeOH in DCM) furnishing **10** (1.08 g, 95%). **^{1}H NMR** (400 MHz, DMSO-*d$_6$*) δ 8.20 (d, *J* = 2.4 Hz, 1H), 8.14–8.06 (m + s, 2H), 7.77 (s, 1H), 7.49 (d, *J* = 9.1 Hz, 1H), 6.68 (s, 2H), 6.08 (d, *J* = 7.5 Hz, 1H), 4.76 (dd, *J* = 7.6, 4.5 Hz, 1H), 4.29 (d, *J* = 5.0 Hz, 1H), 4.04 (m + s, 4H), 3.69 (dd, *J* = 14.4, 6.6 Hz, 1H), 3.13 (dd, *J* = 14.3, 7.2 Hz, 1H), 2.74 (s, 3H), 0.94 (s, 9H), 0.68 (s, 9H), 0.16 (s, 3H), 0.13 (s, 3H), -0.09 (s, 3H), -0.40 (s, 3H). **^{13}C NMR** (101 MHz, DMSO-*d$_6$*) δ 163.9, 157.4, 152.2, 150.7, 134.4, 133.0, 129.9, 127.8, 115.3, 113.4, 103.6, 101.6, 86.5, 83.6, 73.6, 73.4, 57.4, 52.5, 51.9, 36.4, 26.0, 25.6, 17.9, 17.6, -4.5, -4.62, -4.64, -5.5. **LRMS** (ESI) $[M + H]^+$ m/z: 829.1.

General procedure A: Sonogashira coupling reaction

Iodinated nucleoside analogue (1 eq.), $Pd(PPh_3)_4$ (0.1 eq.), CuI (0.3 eq) and (Het)aryl acetylene (2 eq.) were placed in a round bottom flask, which was then filled with argon. Anhydrous THF (0.06 M) was added and the resulting suspension was degassed by repeated evacuation/backfilling with argon. TEA (3 eq.) was added, the flask was once more evacuated and filled with argon. The reaction was stirred at 50–60 °C until the starting material was consumed. The reaction was diluted with EtOAc and washed with water and brine and dried

oved anhydrous Na_2SO_4, filtered and the volatiles were removed on rotary evaporator. The crude product was subjected to flash column chromatography.

***tert*-Butyl 2-((4-amino-7-((2*R*,3*R*,4*R*,5*R*)-3,4-bis((*tert*-butyldimethylsilyl)oxy)-5-(((3-cyano-*N*-ethyl-4-methoxyphenyl)sulfonamido)methyl)tetrahydrofuran-2-yl)-7*H*-pyrrolo[2,3-*d*]pyrimidin-5-yl)ethynyl)-1*H*-benzo[*d*]imidazole-1-carboxylate (11a$_{Boc}$)**

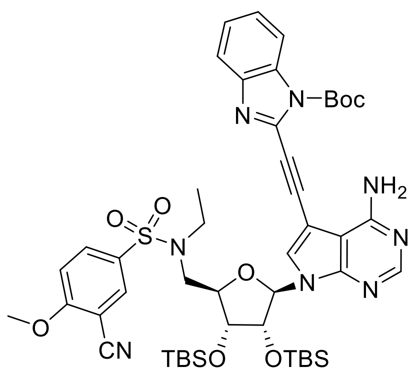

Compound **11a$_{Boc}$** was prepared following General procedure A starting from intermediate **9** (146 mg, 0.173 mmol) and 2-*tert*-butyl 2-ethynyl-1*H*-benzo[d]imidazole-1-carboxylate[8]. Product was purified by flash column chromatography (0-45% of acetone in DCM) followed by reversed phase flash column chromatography (50–100% of ACN in water) providing compound **11a$_{Boc}$** (103 mg, 62%) as an yellowish oil. **^{1}H NMR** (400 MHz, DMSO-*d$_6$*) δ 8.27 (s, 1H), 8.20 (s, 1H), 8.19 (d, *J* = 2.4 Hz, 1H), 8.11 (dd, *J* = 9.0, 2.4 Hz, 1H), 7.92–7.85 (m, 1H), 7.75–7.68 (m, 1H), 7.51–7.37 (m, 3H), 6.10 (d, *J* = 7.3 Hz, 1H), 4.94 (dd, *J* = 7.4, 4.4 Hz, 1H), 4.37 (d, *J* = 4.9 Hz, 1H), 4.08 (t, *J* = 7.0 Hz, 1H), 4.02 (s, 3H), 3.82 (dd, *J* = 15.1, 6.1 Hz, 1H), 3.41 (dd, *J* = 15.3, 7.8 Hz, 1H), 3.27–3.19 (m, 2H), 1.69 (s, 9H), 0.98 (t, *J* = 7.1 Hz, 3H), 0.94 (s, 9H), 0.70 (s, 9H), 0.16 (s, 3H), 0.14 (s, 3H), -0.09 (s, 3H), -0.39 (s, 3H). Signal of amino group was not observed. *N*-Boc group proved to be unstable in DMSO making ^{13}C spectrum ambiguous. **LRMS** (ESI) $[M + H]^+$ m/z: 957.4.

***tert*-Butyl 2-((4-amino-7-((2*R*,3*R*,4*R*,5*R*)-3,4-bis((*tert*-butyldimethylsilyl)oxy)-5-(((3-cyano-*N*-ethyl-4-methoxyphenyl)sulfonamido)methyl)tetrahydrofuran-2-yl)-7*H*-pyrrolo[2,3-*d*]pyrimidin-5-yl)ethynyl)-1*H*-imidazole-1-carboxylate (11b$_{Boc}$)**

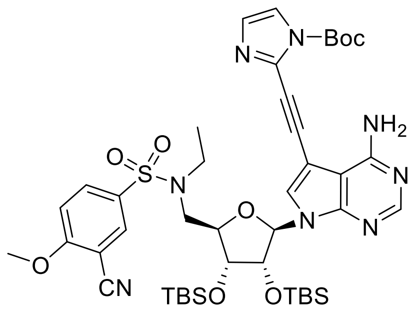

Compound **11b$_{Boc}$** was prepared following General procedure A starting from intermediate **9** (146 mg, 0.173 mmol) and **A3**. Product was purified by flash column chromatography (0-45% of acetone in DCM) followed by reversed phase flash column chromatography (50–100% of ACN in water) giving compound **11b$_{Boc}$** (134 mg, 85%) as a yellowish oil. **^{1}H NMR** (400 MHz, DMSO-*d$_6$*) δ 8.18 (d, *J* = 2.4 Hz, 1H), 8.17 (s, 1H), 8.16 (s, 1H), 8.10 (dd, *J* = 9.0, 2.4 Hz, 1H), 7.62 (d, *J* = 1.6 Hz, 1H), 7.44 (d, *J* = 9.1 Hz, 1H), 7.11 (d, *J* = 1.6 Hz, 1H), 6.08 (d, *J* = 7.4 Hz, 1H), 4.89 (dd, *J* = 7.4, 4.4 Hz, 1H), 4.39–4.33 (m, 1H), 4.09–4.04 (m, 1H), 4.02 (s, 3H), 3.80 (dd, *J* = 15.2, 6.2 Hz, 1H), 3.43–3.36 (m, 1H), 3.26–3.16 (m, 2H), 1.58 (s, 9H), 0.97 (t, *J* = 7.1 Hz, 3H), 0.94 (s, 9H), 0.69 (s, 9H), 0.15 (s, 3H), 0.13 (s, 3H), -0.10 (s, 3H), -0.39 (s, 3H). Signal of amino group was not observed. *N*-Boc group proved to be unstable in DMSO making ^{13}C spectrum ambiguous. **LRMS** (ESI) $[M + H]^+$ m/z: 907.4.

N-(((2R,3R,4R,5R)-5-(4-Amino-5-(phenylethynyl)-7H-pyrrolo[2,3-d]pyrimidin-7-yl)-3,4-bis((tert-butyldimethylsilyl)oxy)tetrahydrofuran-2-yl)methyl)-3-cyano-N-ethyl-4-methoxybenzenesulfonamide (11c)

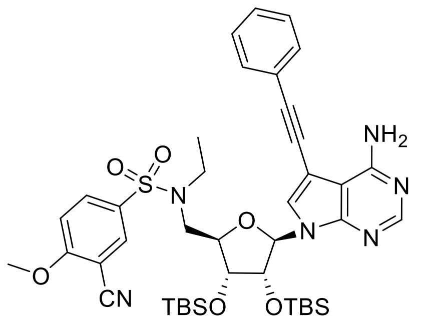

Compound **11c** was prepared following General procedure A starting from intermediate **9** (120 mg, 0.142 mmol) and commercial phenylacetylene. Product was purified by flash column chromatography (0–50% of EtOAc in DCM) yielding **11c** (111 mg, 95%). **^{1}H NMR** (400 MHz, DMSO-d_6) δ 8.19 (d, J = 2.4 Hz, 1H), 8.18 (s, 1H), 8.10 (dd, J = 9.0, 2.4 Hz, 1H), 8.00 (s, 1H), 7.63–7.53 (m, 5H), 7.49–7.37 (m, 4H), 6.74 (s, 2H), 6.09 (d, J = 7.2 Hz, 1H), 4.90 (dd, J = 7.2, 4.4 Hz, 1H), 4.35 (dd, J = 4.1, 1.3 Hz, 1H), 4.10–3.99 (m, 4H), 3.79 (dd, J = 15.1, 6.0 Hz, 1H), 3.38 (dd, J = 14.8, 7.7 Hz, 1H), 3.23 (q, J = 7.3 Hz, 2H), 0.99 (t, J = 7.1 Hz, 3H), 0.94 (s, 9H), 0.71 (s, 9H), 0.16 (s, 3H), 0.14 (s, 3H), -0.08 (s, 3H), -0.37 (s, 3H). **^{13}C NMR** (101 MHz, DMSO-d_6) δ 163.8, 157.8, 153.0, 150.4, 134.1, 132.8, 132.3, 131.3, 128.9, 128.7, 128.0, 122.6, 115.2, 113.3, 101.4, 95.2, 91.4, 87.3, 84.4, 83.1, 73.6, 73.4, 57.4, 49.6, 44.1, 25.9, 25.6, 17.9, 17.6, 13.7, -4.5, -4.6, -4.6, -5.3. **LRMS** (ESI) $[M + H]^+$ m/z: 817.4.

N-(((2R,3R,4R,5R)-5-(4-Amino-5-(naphthalen-1-ylethynyl)-7H-pyrrolo[2,3-d]pyrimidin-7-yl)-3,4-bis((tert-butyldimethylsilyl)oxy)tetrahydrofuran-2-yl)methyl)-3-cyano-N-ethyl-4-methoxybenzenesulfonamide (11d)

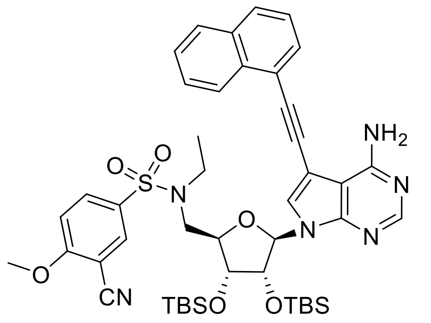

Compound **11d** was prepared following General procedure A starting from intermediate **9** (120 mg, 0.142 mmol) and 1-ethynylnaphthalene[8]. Product was purified by flash column chromatography (0–40% of EtOAc in DCM) affording **11d** (103 mg, 83%). **^{1}H NMR** (400 MHz, DMSO-d_6) δ 8.31 (d, J = 7.8 Hz, 1H), 8.19 (d, J = 2.3 Hz, 2H), 8.14 (s, 1H), 8.10 (dd, J = 9.0, 2.4 Hz, 1H), 8.01 (t, J = 7.8 Hz, 1H), 7.88 (d, J = 7.2 Hz, 1H), 7.68–7.53 (m, 3H), 7.43 (d, J = 9.1 Hz, 1H), 6.78 (s, 2H), 6.11 (d, J = 7.2 Hz, 1H), 4.94 (dd, J = 7.4, 4.4 Hz, 1H), 4.35 (d, J = 4.2 Hz, 1H), 4.11–3.98 (m, 4H), 3.78 (dd, J = 15.1, 5.6 Hz, 1H), 3.45 (dd, J = 15.0, 7.9 Hz, 1H), 3.25 (q, J = 7.1 Hz, 2H), 1.00 (t, J = 7.1 Hz, 3H), 0.94 (s, 9H), 0.71 (s, 9H), 0.16 (s, 3H), 0.14 (s, 3H), -0.07 (s, 3H), -0.35 (s, 3H). **^{13}C NMR** (101 MHz, DMSO-d_6) δ 163.8, 157.9, 153.1, 150.5, 134.1, 133.1, 132.8, 132.5, 132.3, 130.4, 129.0, 128.8, 128.5, 127.4, 126.9, 125.8, 125.4, 120.1, 115.2, 113.3, 101.6, 95.3, 89.3, 88.0, 87.4, 84.4, 73.7, 73.5, 59.9, 57.4, 49.6, 44.0, 25.9, 25.7, 17.9, 17.6, 13.7, -4.5, -4.56 -4.59, -5.3. **LRMS** (ESI) $[M + H]^+$ m/z: 867.2.

***N*-(((2*R*,3*R*,4*R*,5*R*)-5-(4-Amino-5-(quinolin-3-ylethynyl)-7*H*-pyrrolo[2,3-*d*]pyrimidin-7-yl)-3,4-bis((*tert*-butyldimethylsilyl)oxy)tetrahydrofuran-2-yl)methyl)-3-cyano-*N*-ethyl-4-methoxybenzenesulfonamide (11e)**

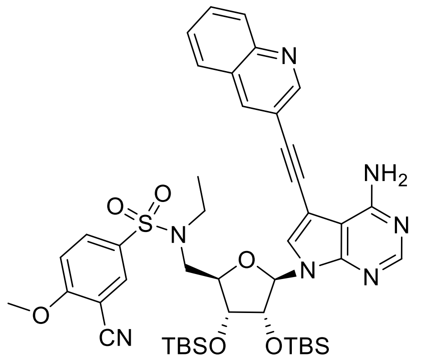

Compound **11e** was prepared following General procedure A starting from intermediate **9** (120 mg, 0.142 mmol) and 3-ethynylquinoline[8]. Product was purified by flash column chromatography (0–55% of acetone in DCM) providing **11e** (106 mg, 86%). **^{1}H NMR** (400 MHz, DMSO-d_6) δ 9.04 (d, *J* = 2.1 Hz, 1H), 8.64 (d, *J* = 2.0 Hz, 1H), 8.20 (d, *J* = 2.4 Hz, 1H), 8.19 (s, 1H), 8.14–8.08 (m, 2H), 8.07–8.04 (m, 1H), 8.02 (dd, *J* = 8.7, 1.4 Hz, 1H), 7.81 (ddd, *J* = 8.4, 6.9, 1.5 Hz, 1H), 7.68 (ddd, *J* = 8.1, 6.8, 1.1 Hz, 1H), 7.44 (d, *J* = 9.1 Hz, 1H), 6.87 (s, 2H), 6.11 (d, *J* = 7.2 Hz, 1H), 4.93 (dd, *J* = 7.3, 4.4 Hz, 1H), 4.37 (d, *J* = 5.3 Hz, 1H), 4.08 (t, *J* = 7.0 Hz, 1H), 3.80 (dd, *J* = 15.1, 6.1 Hz, 1H), 3.40 (dd, *J* = 15.2, 7.6 Hz, 1H), 3.24 (q, *J* = 7.2 Hz, 2H), 1.00 (t, *J* = 7.1 Hz, 3H), 0.95 (s, 9H), 0.72 (s, 9H), 0.17 (s, 3H), 0.15 (s, 3H), -0.07 (s, 3H), -0.36 (s, 3H). **^{13}C NMR** (101 MHz, DMSO-d_6) δ 163.8, 157.8, 153.1, 151.8, 150.5, 146.3, 138.2, 134.1, 132.8, 132.2, 130.5, 129.0, 128.8, 128.2, 127.7, 127.0, 117.0, 115.2, 113.3, 102.4, 101.5, 94.9, 88.9, 87.4, 86.2, 84.4, 73.6, 73.4, 57.4, 49.6, 44.1, 25.9, 25.7, 17.9, 17.6, 13.7, -4.5, -4.6, -4.6, -5.3. **LRMS** (ESI) [M + H]$^{+}$ m/z: 868.3.

***N*-(((2*R*,3*R*,4*R*,5*R*)-5-(4-Amino-5-((6-hydroxypyridin-3-yl)ethynyl)-7*H*-pyrrolo[2,3-*d*]pyrimidin-7-yl)-3,4-bis((*tert*-butyldimethylsilyl)oxy)tetrahydrofuran-2-yl)methyl)-3-cyano-*N*-ethyl-4-methoxybenzenesulfonamide (11f)**

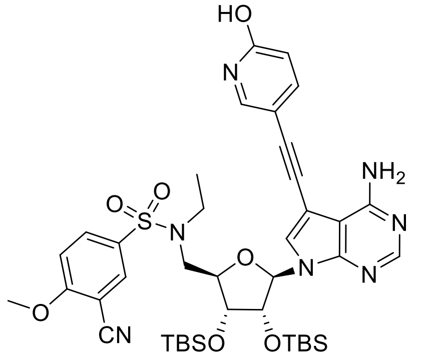

Compound **11f** was prepared following modified General procedure starting from intermediate **9** (96 mg, 0.11 mmol) and **A1**. Reaction mixture was directly adsorbed onto silica gel and subjected to flash column chromatography (2–15% of MeOH in DCM) furnishing **11f** (52 mg, 60%). **^{1}H NMR** (400 MHz, DMSO-d_6) δ 11.96 (s, 1H), 8.18 (d, *J* = 2.4 Hz, 1H), 8.16 (s, 1H), 8.09 (dd, *J* = 9.0, 2.4 Hz, 1H), 7.92 (s, 1H), 7.82 (d, *J* = 2.4 Hz, 1H), 7.54 (dd, *J* = 9.5, 2.6 Hz, 1H), 7.43 (d, *J* = 9.1 Hz, 1H), 6.80 (bs, 2H) 6.36 (d, *J* = 9.8 Hz, 1H), 6.06 (d, *J* = 7.3 Hz, 1H), 4.87 (dd, *J* = 7.2, 4.4 Hz, 1H), 4.33 (d, *J* = 5.3 Hz, 1H), 4.08–3.98 (m + s, 4H), 3.76 (dd, *J* = 15.0, 6.0 Hz, 1H), 3.35 (dd, *J* = 15.0, 7.5 Hz, 1H), 3.21 (q, *J* = 7.2 Hz, 2H), 0.97 (t, *J* = 7.1 Hz, 3H), 0.93 (s, 9H), 0.69 (s, 9H), 0.15 (s, 3H), 0.13 (s, 3H), -0.10 (s, 3H), -0.39 (s, 3H). **^{13}C NMR** (101 MHz, DMSO-d_6) δ 163.8, 161.3, 157.3*,152.0*, 150.1, 142.8, 139.9*, 134.1, 132.8, 132.2, 127.8, 115.2, 113.4, 102.4*, 101.4, 100.7*, 95.7, 87.8, 87.3, 84.4, 82.2, 73.6, 73.4, 57.4, 49.6, 44.1, 25.9, 25.7, 17.9, 17.6, 13.7, -4.5, -4.58, -4.61, -5.3. **LRMS** (ESI) [M + H]$^{+}$ m/z: 834.4.

_N_-(((2_R_,3_R_,4_R_,5_R_)-5-(4-Amino-5-((2-((4-methoxybenzyl)oxy)pyrimidin-5-yl)ethynyl)-7_H_-pyrrolo[2,3-_d_]pyrimidin-7-yl)-3,4-bis((_tert_-butyldimethylsilyl)oxy)tetrahydrofuran-2-yl)methyl)-3-cyano-_N_-ethyl-4-methoxybenzenesulfonamide (11gPMB)

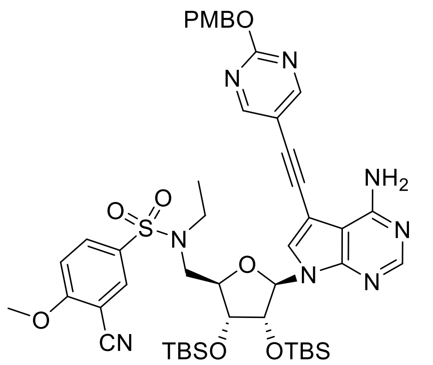

Compound **11gPMB** was prepared following General procedure A starting from intermediate **9** (119 mg, 0.141 mmol) and **A2**. Crude product was subjected to flash column chromatography (5–20% of EtOAc/EtOH (4:1) mixture in DCM) followed by reverse phase column chromatography (50–100% of ACN in water) giving compound **11gPMB** (129 mg, 96%). **^{1}H NMR** (400 MHz, DMSO-d_6) δ 8.85 (s, 2H), 8.19 (d, *J* = 2.3 Hz, 1H), 8.16 (s, 1H), 8.09 (dd, *J* = 9.0, 2.3 Hz, 1H), 8.02 (s, 1H), 7.42 (m, 3H), 6.95 (d, *J* = 8.7 Hz, 2H), 6.81 (s, 2H), 6.08 (d, *J* = 7.2 Hz, 1H), 5.36 (s, 2H), 4.90 (dd, *J* = 7.2, 4.4 Hz, 1H), 4.35 (d, *J* = 4.4 Hz, 1H), 4.05 (t, *J* = 6.8 Hz, 1H), 4.01 (s, 3H), 3.76 (m + s, 4H), 3.40–3.34 (m, 1H), 3.22 (q, *J* = 7.1 Hz, 2H), 0.98 (t, *J* = 7.1 Hz, 3H), 0.94 (s, 9H), 0.70 (s, 9H), 0.16 (s, 3H), 0.13 (s, 3H), -0.09 (s, 3H), -0.39 (s, 3H). **^{13}C NMR** (101 MHz, DMSO-d_6) δ 164.1, 163.4, 161.9, 159.7, 158.0, 153.3, 150.7, 134.4, 133.1, 132.5, 130.5, 128.8, 128.6, 115.5, 114.3, 113.6, 113.0, 102.7, 101.7, 95.1, 87.7, 87.7, 85.2, 84.7, 73.9, 73.7, 69.2, 57.7, 55.6, 49.9, 44.4, 26.2, 25.9, 18.2, 17.9, 14.0, -4.2, -4.29, -4.33, -5.0. **LRMS** (ESI) $[M + H]^+$ m/z: 955.2.

_N_-(((2_R_,3_R_,4_R_,5_R_)-5-(4-Amino-5-((6-aminopyridin-3-yl)ethynyl)-7_H_-pyrrolo[2,3-_d_]pyrimidin-7-yl)-3,4-bis((tert-butyldimethylsilyl)oxy)tetrahydrofuran-2-yl)methyl)-3-cyano-_N_-ethyl-4-methoxybenzenesulfonamide (11h)

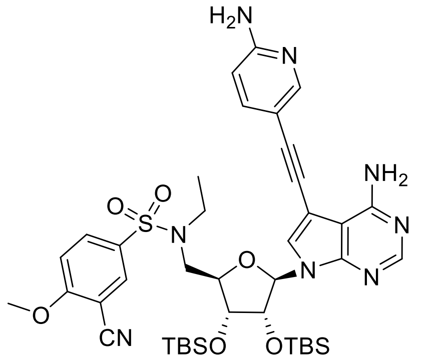

Compound **11h** was prepared following General procedure A starting from intermediate **9** (88 mg, 0.10 mmol) and 2-amino-5-ethynylpyridine[1]. Crude product was subjected to reverse phase chromatography (40–100% of ACN in water) yielding **11h** (76 mg, 87%). **^{1}H NMR** (400 MHz, DMSO-d_6) δ 8.19 (d, *J* = 2.2 Hz, 1H), 8.14 (m, 2H), 8.09 (dd, *J* = 9.0, 2.2 Hz, 1H), 7.89 (s, 1H), 7.53 (dd, *J* = 8.6, 2.2 Hz, 1H), 7.43 (d, *J* = 9.1 Hz, 1H), 6.66 (bs, 2H), 6.45 (d, *J* = 8.6 Hz, 1H), 6.39 (s, 2H), 6.06 (d, *J* = 7.2 Hz, 1H), 4.87 (dd, *J* = 7.1, 4.4 Hz, 1H), 4.33 (d, *J* = 4.1 Hz, 1H), 4.09–3.95 (m + s, 4H), 3.77 (dd, *J* = 15.0, 6.0 Hz, 1H), 3.40–3.29 (m, 1H), 3.22 (q, *J* = 7.0 Hz, 2H), 0.97 (t, *J* = 7.1 Hz, 3H), 0.93 (s, 9H), 0.70 (s, 9H), 0.15 (s, 3H), 0.13 (s, 3H), -0.10 (s, 3H), -0.39 (s, 3H). **^{13}C NMR** (101 MHz, DMSO-d_6) δ 163.8, 159.3, 157.8, 152.9, 151.2, 150.3, 139.5, 134.1, 132.8, 132.3, 127.0, 115.2, 113.4, 107.7, 106.4, 102.5, 101.4, 95.9, 90.0, 87.2, 84.3, 82.3, 73.6, 73.4, 57.4, 49.6, 44.1, 25.9, 25.7, 17.9, 17.6, 13.7, -4.5, -4.58, -4.61, -5.3. **LRMS** (ESI) $[M + H]^+$ m/z: 833.4.

_N_-(((2_R_,3_R_,4_R_,5_R_)-5-(4-Amino-5-((2-aminopyrimidin-5-yl)ethynyl)-7_H_-pyrrolo[2,3-_d_]pyrimidin-7-yl)-3,4-bis((_tert_-butyldimethylsilyl)oxy)tetrahydrofuran-2-yl)methyl)-3-cyano-_N_-ethyl-4-methoxybenzenesulfonamide (11i)

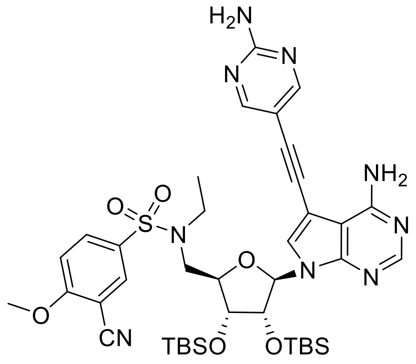

Compound **11i** was prepared following General procedure A starting from intermediate **9** (96 mg, 0.11 mmol) and 2-amino-5-ethynylpyrimidine[2]. Crude product was subjected to flash column chromatography (2–10% of MeOH in DCM) followed by reverse phase chromatography (50–100% of ACN in water) providing **11i** (76 mg, 87%). **^{1}H NMR** (400 MHz, DMSO-d_6) δ 8.45 (s, 2H), 8.18 (d, J = 2.1 Hz, 1H), 8.14 (s, 1H), 8.09 (dd, J = 9.0, 2.1 Hz, 1H), 7.92 (s, 1H), 7.43 (d, J = 9.1 Hz, 1H), 7.10 (s, 2H), 6.72 (s, 2H), 6.07 (d, J = 7.2 Hz, 1H), 4.88 (dd, J = 6.9, 4.5 Hz, 1H), 4.34 (d, J = 3.9 Hz, 1H), 4.09–3.96 (m + s, 4H), 3.77 (dd, J = 15.0, 6.0 Hz, 1H), 3.40–3.31* (m, 1H) 3.22 (q, J = 6.9 Hz, 2H), 0.97 (t, J = 7.1 Hz, 3H), 0.93 (s, 9H), 0.70 (s, 9H), 0.15 (s, 3H), 0.13 (s, 3H), -0.10 (s, 3H), -0.39 (s, 3H). **^{13}C NMR** (101 MHz, DMSO-d_6) δ 163.8, 162.0, 160.4, 157.8, 153.0, 150.3, 134.1, 132.8, 132.2, 127.5, 115.2, 113.4, 106.3, 102.4, 101.5, 95.5, 87.3, 86.7, 85.1, 84.3, 73.6, 73.4, 57.4, 49.6, 44.1, 25.9, 25.7, 17.9, 17.6, 13.7, -4.4, -4.58, -4.62, -5.3. **LRMS** (ESI) [M + H]$^+$ m/z: 834.3.

_N_-(((2_R_,3_R_,4_R_,5_R_)-5-(4-Amino-5-((2,4-dioxo-1,2,3,4-tetrahydropyrimidin-5-yl)ethynyl)-7_H_-pyrrolo[2,3-_d_]pyrimidin-7-yl)-3,4-bis((_tert_-butyldimethylsilyl)oxy)tetrahydrofuran-2-yl)methyl)-3-cyano-_N_-ethyl-4-methoxybenzenesulfonamide (11j)

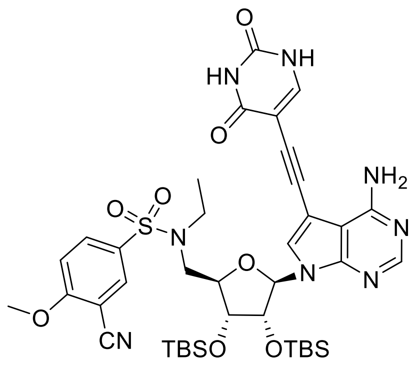

Compound **11j** was prepared using modified General procedure A starting from intermediate **9** (88 mg, 0.104 mmol) and 5-ethynyluracil[4]. DMF was used as solvent and amount of TEA increased to 10 equivalents. Product was purified on reverse phase flash column chromatography (40-100% of ACN in water) affording **11j** (50 mg, 56%). **^{1}H NMR** (400 MHz, DMSO-d_6) δ 11.57 (s, 1H), 11.39 (s, 1H), 8.18 (d, J = 2.3 Hz, 1H), 8.11 (s, 1H), 8.08 (dd, J = 9.0, 2.3 Hz, 2H), 7.85 (s, 1H), 7.79 (s, 1H), 7.43 (d, J = 9.1 Hz, 1H), 7.20 (bs, 2H), 6.03 (d, J = 7.3 Hz, 1H), 4.92 (dd, J = 7.2, 4.4 Hz, 1H), 4.34 (d, J = 4.2 Hz, 1H), 4.07–3.98 (m, 4H), 3.78 (dd, J = 15.0, 6.0 Hz, 1H), 3.40–3.32 (m, 1H), 3.27–3.16 (m, 2H), 0.97 (t, J = 7.1 Hz, 3H), 0.93 (s, 9H), 0.69 (s, 9H), 0.15 (s, 3H), 0.13 (s, 3H), -0.11 (s, 3H), -0.41 (s, 3H). **^{13}C NMR** (101 MHz, DMSO-d_6) δ 164.0, 163.8, 157.8, 152.9, 150.6, 150.3, 143.7, 134.1, 132.8, 132.3, 125.7, 115.2, 113.4, 102.8, 101.4, 97.2, 95.5, 87.6, 87.1, 84.9, 84.5, 73.5, 73.4, 57.4, 49.6, 44.1, 25.9, 25.7, 18.0, 17.6, 13.7, -4.4, -4.6, -4.6, -5.3. **LRMS** (ESI) [M + H]$^+$ m/z: 851.3.

_N_-(((2_R_,3_R_,4_R_,5_R_)-5-(4-Amino-5-((4-amino-2-oxo-1,2-dihydropyrimidin-5-yl)ethynyl)-7_H_-pyrrolo[2,3-_d_]pyrimidin-7-yl)-3,4-bis((_tert_-butyldimethylsilyl)oxy)tetrahydrofuran-2-yl)methyl)-3-cyano-_N_-ethyl-4-methoxybenzenesulfonamide (11k)

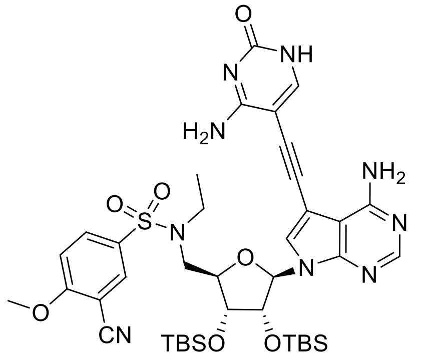

Starting material **9** (84 mg, 0.10 mmol), 4-ethynylcytosine[5] (28 mg, 0.21 mmol, 2eq.), $Pd(PPh_3)_2Cl_2$ (4 mg, 0.06 mmol, 5.5 mol%) and CuI (2 mg, 0.011 mmol, 10 mol%) were placed in a round bottom flask, which was then filled with argon. Anhydrous DMF (1.0 mL) and TEA (0.145 mL, 10 eq.) were added, and the resulting suspension was degassed by repeated evacuation/backfilling with argon. The reaction was stirred at 80 °C overnight. DMF was evaporated and the residue was diluted with EtOAc and washed with water and brine and dried oved anhydrous Na_2SO_4. The crude product was subjected to reverse phase flash column chromatography (40-100% of ACN in water, 0.1% of FA as modifier). Impure fractions were further purified on silica (0–30% of EtOAc/acetone/EtOH/water mixture (17/3/3/2) in EtOAc/acetone/EtOH/water (21/3/0.6/0.4). This way compound **11k** (33 mg, 37%) was obtained. **^{1}H NMR** (400 MHz, DMSO-d_6) δ 10.99 (s, 1H), 8.17 (d, *J* = 2.2 Hz, 1H), 8.14 (s, 1H), 8.08 (dd, *J* = 9.0, 2.2 Hz, 2H), 7.96–7.87 (m, 1H), 7.63 (bs, 1H), 7.43 (d, *J* = 9.1 Hz, 1H), 6.93–6.57 (2xbs, 3H), 6.00 (d, *J* = 7.1 Hz, 1H), 4.91 (dd, *J* = 7.0, 4.3, 1H), 4.35 (d, *J* = 4.0 Hz, 1H), 4.08–3.96 (m + s, 4H), 3.78 (dd, *J* = 15.0, 5.8 Hz, 1H), 3.37–3.29* (m, 1H) 3.22 (q, *J* = 7.2 Hz, 2H), 1.01–0.90 (m + s, 12H), 0.70 (s, 9H), 0.15 (s, 3H), 0.13 (s, 3H), -0.10 (s, 3H), -0.39 (s, 3H). **^{13}C NMR** (101 MHz, DMSO-d_6) δ 163.8, 157.9, 152.8, 150.1, 147.1, 134.1, 132.8, 132.2, 128.4, 115.2, 113.4, 101.4, 95.8, 87.8, 87.2, 84.3, 73.4 (2C)*, 57.4, 49.5, 44.1, 25.9, 25.7, 17.9, 17.6, 13.7, -4.4, -4.6, -4.6, -5.2. Some ^{13}C signals were not observed. **LRMS** (ESI) $[M + H]^+$ m/z: 850.3.

_N_-(((2_R_,3_R_,4_R_,5_R_)-5-(4-Amino-5-(pyridin-3-ylethynyl)-7_H_-pyrrolo[2,3-_d_]pyrimidin-7-yl)-3,4-bis((_tert_-butyldimethylsilyl)oxy)tetrahydrofuran-2-yl)methyl)-3-cyano-_N_-ethyl-4-methoxybenzenesulfonamide (11l)

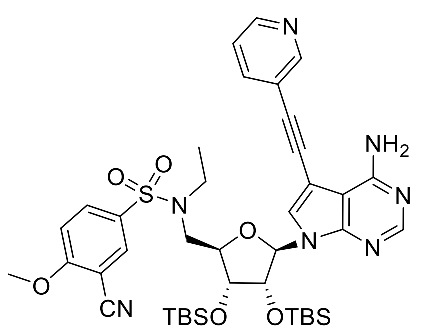

Compound **11l** was prepared following General procedure A starting from intermediate **9** (104 mg, 0.124 mmol) and 3-ethynylpyridine[12]. Product was purified on silica (2–15% of MeOH in DCM) followed by reverse phase chromatography (50–100% of ACN in water) providing **11l** (73 mg, 72%). **^{1}H NMR** (400 MHz, DMSO-d_6) δ 8.81 (s, 1H), 8.58 (s, 1H), 8.19 (d, *J* = 2.4 Hz, 1H), 8.17 (s, 1H), 8.09 (dd, *J* = 9.0, 2.4 Hz, 1H), 8.05 (s, 1H), 8.01 (d, *J* = 7.9 Hz, 1H), 7.50–7.44 (m, 1H), 7.43 (d, *J* = 9.1 Hz, 1H), 6.79 (s, 2H), 6.09 (d, *J* = 7.3 Hz, 1H), 4.90 (dd, *J* = 7.2, 4.4 Hz, 1H), 4.34 (d, *J* = 4.1 Hz, 1H), 4.05 (t, *J* = 6.7 Hz, 1H), 4.01 (s, 3H), 3.78 (dd, *J* = 15.0, 6.1 Hz, 1H), 3.37 (dd, *J* = 15.1, 7.7 Hz, 1H), 3.22 (q, *J* = 7.0 Hz, 2H), 0.98 (t, *J* = 7.1 Hz, 3H), 0.94 (s, 9H), 0.70 (s, 9H), 0.16 (s, 3H), 0.13 (s, 3H), -0.09 (s, 3H), -0.39 (s, 3H). **^{13}C NMR** (101 MHz, DMSO-d_6) δ 163.8, 157.8, 153.1, 151.7, 150.5, 148.8, 138.5, 134.1, 132.8, 132.2, 128.7, 123.9*, 115.2, 113.4, 102.5*, 101.5, 94.8, 88.3, 87.3, 86.2, 84.4, 73.6, 73.4, 57.4, 49.6, 44.1, 25.9, 25.7, 17.9, 17.6, 13.7, -4.5, -4.6, -4.6, -5.3. Signal of one carbon was not observed. **LRMS** (ESI) $[M + H]^+$ m/z: 818.3.

N-(((2R,3R,4R,5R)-5-(4-Amino-5-(pyrimidin-5-ylethynyl)-7H-pyrrolo[2,3-d]pyrimidin-7-yl)-3,4-bis((tert-butyldimethylsilyl)oxy)tetrahydrofuran-2-yl)methyl)-3-cyano-N-ethyl-4-methoxybenzenesulfonamide (11m)

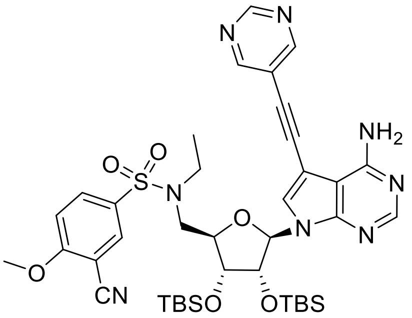

Compound **11m** was prepared following General procedure A starting from intermediate **9** (104 mg, 0.124 mmol) and 4-ethynylpyrimidine[10]. Product was purified on silica (2–10% of MeOH in DCM) followed by reverse phase chromatography (70–100% of ACN in water) giving compound **11m** (85 mg, 84%). **^{1}H NMR** (400 MHz, DMSO-d_6) δ 9.17 (s, 1H), 9.03 (s, 2H), 8.19 (d, *J* = 2.4 Hz, 1H), 8.17 (s, 1H), 8.13–8.06 (m + s, 2H), 7.43 (d, *J* = 9.1 Hz, 1H), 6.86 (s, 2H), 6.09 (d, *J* = 7.3 Hz, 1H), 4.92 (dd, *J* = 7.3, 4.4 Hz, 1H), 4.35 (d, *J* = 5.0 Hz, 1H), 4.06 (t, *J* = 6.9 Hz, 1H), 4.01 (s, 3H), 3.78 (dd, *J* = 15.1, 6.1 Hz, 1H), 3.37 (dd, *J* = 15.1, 7.6 Hz, 1H), 3.22 (q, *J* = 7.2 Hz, 2H), 0.98 (t, *J* = 7.1 Hz, 3H), 0.94 (s, 9H), 0.70 (s, 9H), 0.16 (s, 3H), 0.13 (s, 3H), -0.09 (s, 3H), -0.39 (s, 3H). **^{13}C NMR** (101 MHz, DMSO-d_6) δ 163.8, 158.7, 157.7, 156.6, 153.1, 150.5, 134.1, 132.8, 132.2, 129.2, 119.5, 115.2, 113.4, 102.3, 101.5, 94.4, 89.6, 87.4, 85.0, 84.5, 73.6, 73.4, 57.4, 49.6, 44.2, 25.9, 25.7, 17.9, 17.6, 13.7, -4.5, -4.6, -4.6, -5.3. **LRMS** (ESI) $[M + H]^+$ m/z: 819.4.

N-(((2R,3R,4R,5R)-5-(4-Amino-5-(pyridin-4-ylethynyl)-7H-pyrrolo[2,3-d]pyrimidin-7-yl)-3,4-bis((tert-butyldimethylsilyl)oxy)tetrahydrofuran-2-yl)methyl)-3-cyano-N-ethyl-4-methoxybenzenesulfonamide (11n)

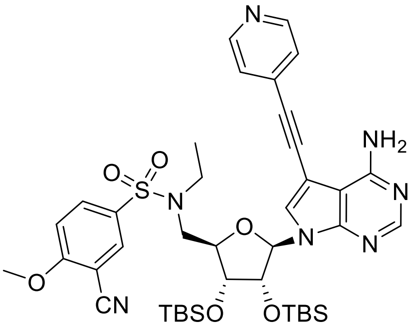

Compound **11n** was prepared following General procedure starting from intermediate **9** (104 mg, 0.124 mmol) and 4-ethynylpyridine[9]. Product was purified on silica (2–10% of MeOH in DCM) followed by reverse phase chromatography (40–100% of ACN in water, 0.1% of FA as modifier) yielding pure **11m** (83 mg, 80%). **^{1}H NMR** (400 MHz, DMSO-d_6) δ 8.64–8.58 (m, 2H), 8.19 (d, *J* = 2.4 Hz, 1H), 8.18 (s, 1H), 8.12–8.07 (m + s, 2H), 7.58–7.52 (m, 2H), 7.43 (d, *J* = 9.1 Hz, 1H), 6.80 (s, 2H), 6.09 (d, *J* = 7.3 Hz, 1H), 4.90 (dd, *J* = 7.3, 4.4 Hz, 1H), 4.34 (d, *J* = 5.3 Hz, 1H), 4.06 (t, *J* = 7.1 Hz, 1H), 4.01 (s, 3H), 3.78 (dd, *J* = 15.1, 6.1 Hz, 1H), 3.38 (dd, *J* = 15.1, 7.6 Hz, 1H), 3.22 (q, *J* = 7.1 Hz, 2H), 0.98 (t, *J* = 7.1 Hz, 3H), 0.94 (s, 9H), 0.69 (s, 9H), 0.16 (s, 3H), 0.13 (s, 3H), -0.09 (s, 3H), -0.39 (s, 3H). **^{13}C NMR** (101 MHz, DMSO-d_6) δ 163.8, 157.7, 153.2, 150.6, 150.0, 134.1, 132.8, 132.2, 130.7, 129.5, 125.2, 115.2, 113.4, 102.3, 101.5, 94.4, 89.1, 87.8, 87.4, 84.5, 73.6, 73.4, 57.4, 49.5, 44.1, 25.9, 25.6, 17.9, 17.6, 13.7, -4.5, -4.59, -4.62, -5.3. **LRMS** (ESI) $[M + H]^+$ m/z: 818.3.

4-((4-Amino-7-((2R,3R,4R,5R)-3,4-bis((tert-butyldimethylsilyl)oxy)-5-(((3-cyano-N-ethyl-4-methoxyphenyl)sulfonamido)methyl)tetrahydrofuran-2-yl)-7H-pyrrolo[2,3-d]pyrimidin-5-yl)ethynyl)benzoic acid (11o)

Nucleoside derivative **9** (104 mg, 0.12 mmol), 4-ethynylbenzoic acid[3, 6] (36 mg, 0.25 mmol, 2eq.), $Pd(PPh_3)_4$ (14 mg, 0.012 mmol, 10 mol%) and CuI (7 mg, 0.037 mmol, 30 mol%) were placed in a round bottom flask, which was then filled with argon. Anhydrous DMF (2 mL) and TEA (0.5 mL) were added, and the resulting suspension was degassed by repeated

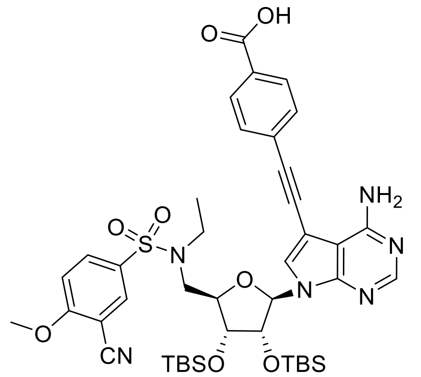

evacuation/backfilling with argon. The reaction was stirred at 50–60 °C for 3 hours. DMF was evaporated and the residue was diluted with EtOAc and washed with 0.2 M hydrochloric acid and brine and dried oved anhydrous Na_2SO_4, filtered and the volatiles were removed on rotary evaporator. The crude product was subjected to reverse phase flash column chromatography (40–100% of ACN in water, 0.1% of FA as modifier) giving **11o** (66 mg, 62%). **^{1}H NMR** (400 MHz, DMSO-*d6*) δ 13.09 (s, 1H), 8.19 (d, *J* = 2.3 Hz, 1H), 8.18 (s, 1H), 8.10 (dd, *J* = 9.0, 2.4 Hz, 1H), 8.07 (s, 1H), 7.96 (d, *J* = 8.4 Hz, 2H), 7.69 (d, *J* = 8.4 Hz, 2H), 7.43 (d, *J* = 9.1 Hz, 1H), 6.80 (s, 1H), 6.09 (d, *J* = 7.2 Hz, 1H), 4.89 (dd, *J* = 7.1, 4.4 Hz, 1H), 4.34 (d, *J* = 4.4 Hz, 1H), 4.06 (s, 1H), 4.01 (s, 3H), 3.78 (dd, *J* = 15.0, 6.0 Hz, 1H), 3.38 (dd, *J* = 15.2, 7.8 Hz, 1H), 3.23 (q, *J* = 7.1 Hz, 2H), 0.98 (t, *J* = 7.1 Hz, 3H), 0.94 (s, 9H), 0.70 (s, 9H), 0.16 (s, 3H), 0.13 (s, 3H), -0.09 (s, 3H), -0.38 (s, 3H). **^{13}C NMR** (101 MHz, DMSO-*d6*) δ 166.9, 163.8, 157.7, 153.1, 150.5, 134.1, 132.8, 132.3, 131.4, 130.3, 129.7, 128.8, 127.1, 115.2, 113.4, 102.4, 101.5, 94.9, 90.9, 87.3, 86.0, 84.4, 73.7, 73.4, 57.4, 49.6, 44.1, 25.9, 25.7, 17.9, 17.6, 13.7, -4.5, -4.58, -4.61, -5.3. **LRMS** (ESI) $[M + H]^+$ m/z: 861.3.

4-((4-Amino-7-((2*R*,3*R*,4*R*,5*R*)-3,4-bis((*tert*-butyldimethylsilyl)oxy)-5-(((3-cyano-*N*-ethyl-4-methoxyphenyl)sulfonamido)methyl)tetrahydrofuran-2-yl)-7*H*-pyrrolo[2,3-*d*]pyrimidin-5-yl)ethynyl)benzamide (11p)

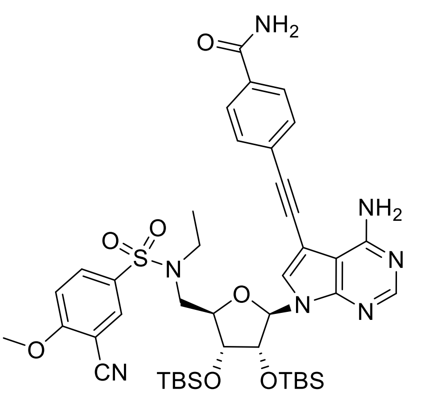

Compound **11p** was prepared following General procedure starting from intermediate **9** (104 mg, 0.124 mmol) and 4-ethynylbenzamide[7, 11]. Crude product was subjected to flash column chromatography (5–20% of EtOAc/EtOH (4:1) mixture in DCM) followed by reverse phase column chromatography (50–100% of ACN in water) furnishing compound **11p** (129 mg, 96%). **^{1}H NMR** (400 MHz, DMSO-*d6*) δ 8.19 (d, *J* = 2.4 Hz, 1H), 8.17 (s, 1H), 8.10 (dd, *J* = 9.0, 2.4 Hz, 1H), 8.05 (s, 1H), 8.04 (s, 1H), 7.91 (m, 2H), 7.65 (m, 2H), 7.46 (s, 1H), 7.43 (d, *J* = 9.1 Hz, 1H), 6.76 (s, 2H), 6.09 (d, *J* = 7.2 Hz, 1H), 4.90 (dd, *J* = 7.2, 4.4 Hz, 1H), 4.35 (d, *J* = 4.6 Hz, 1H), 4.05 (t, *J* = 6.9 Hz, 1H), 4.01 (s, 3H), 3.78 (dd, *J* = 15.1, 6.0 Hz, 1H), 3.37 (dd, *J* = 15.0, 7.6 Hz, 1H), 3.23 (q, *J* = 7.1 Hz, 2H), 0.98 (t, *J* = 7.1 Hz, 3H), 0.94 (s, 9H), 0.70 (s, 9H), 0.16 (s, 3H), 0.13 (s, 3H), -0.09 (s, 3H), -0.39 (s, 3H). **^{13}C NMR** (101 MHz, DMSO-*d6*) δ 167.3, 163.8, 157.8, 153.1, 150.5, 134.1, 133.9, 132.8, 132.3, 131.1, 128.6, 127.9, 125.5, 115.2, 113.4, 102.5, 101.5, 95.0, 91.0, 87.3, 85.1, 84.4, 73.6, 73.4, 57.4, 49.6, 44.1, 25.9, 25.7, 17.9, 17.6, 13.7, -4.5, -4.58, -4.61, -5.3. **LRMS** (ESI) $[M + H]^+$ m/z: 860.3.

***N*-(((2*R*,3*R*,4*R*,5*R*)-5-(4-Amino-5-(quinolin-3-ylethynyl)-7*H*-pyrrolo[2,3-*d*]pyrimidin-7-yl)-3,4-bis((*tert*-butyldimethylsilyl)oxy)tetrahydrofuran-2-yl)methyl)-3-cyano-4-methoxy-*N*-methylbenzenesulfonamide (11q)**

Compound **11q** was prepared using General procedure starting from intermediate **10** (130 mg, 0.157 mmol) and 3-ethynylquinoline[8]. Product was purified on flash column chromatography

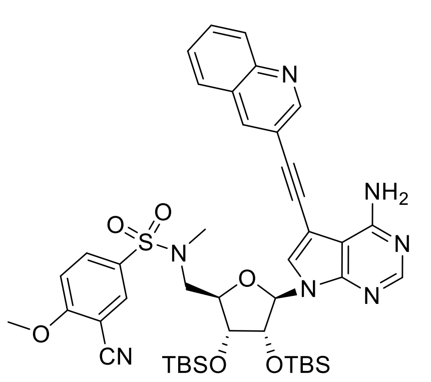

(2–15% of EtOH in DCM) followed by reverse phase flash column chromatography (50–100% ACN in water, 0.1% of FA as modifier) affording **11q** (97 mg, 72%). **^{1}H NMR** (400 MHz, DMSO-d_6) δ 9.03 (d, J = 2.1 Hz, 1H), 8.64 (d, J = 1.6 Hz, 1H), 8.21 (d, J = 2.3 Hz, 1H), 8.18 (s, 1H), 8.12–8.07 (m + s, 2H), 8.05 (d, J = 8.4 Hz, 1H), 8.01 (dd, J = 8.7, 1.4 Hz, 1H), 7.81 (ddd, J = 8.4, 6.9, 1.5 Hz, 1H), 7.72–7.63 (m, 1H), 7.49 (d, J = 9.1 Hz, 1H), 6.86 (bs, 2H), 6.13 (d, J = 7.4 Hz, 1H), 4.86 (dd, J = 7.4, 4.5 Hz, 1H), 4.34 (d, J = 4.4 Hz, 1H), 4.07 (t, J = 7.0 Hz, 1H), 4.03 (s, 3H), 3.76 (dd, J = 14.3, 6.7 Hz, 1H), 3.14 (dd, J = 14.4, 7.2 Hz, 1H), 2.76 (s, 3H), 0.95 (s, 9H), 0.70 (s, 9H), 0.17 (s, 3H), 0.14 (s, 3H), -0.07 (s, 3H), -0.37 (s, 3H). **^{13}C NMR** (101 MHz, DMSO-d_6) δ 163.9, 157.8, 153.1, 151.8, 150.6, 146.3, 138.2, 134.4, 133.1, 130.5, 129.9, 129.0, 128.6, 128.2, 127.7, 127.1, 117.0, 115.3, 113.4, 102.3, 101.6, 95.0, 88.9, 87.1, 86.2, 83.8, 73.7, 73.5, 57.4, 51.9, 36.5, 25.9, 25.6, 18.0, 17.6, -4.5, -4.6 (2C), -5.3. **LRMS** (ESI) [M + H]$^+$ m/z: 854.3.

4-((4-Amino-7-((2*R*,3*R*,4*R*,5*R*)-3,4-bis((*tert*-butyldimethylsilyl)oxy)-5-(((3-cyano-4-methoxy-*N*-methylphenyl)sulfonamido)methyl)tetrahydrofuran-2-yl)-7*H*-pyrrolo[2,3-*d*]pyrimidin-5-yl)ethynyl)benzamide (11r)

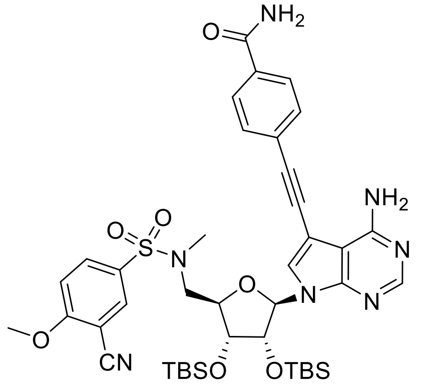

Compound **11r** was prepared using General procedure starting from intermediate **10** (130 mg, 0.157 mmol) and 4-ethynylbenzamide[7,11]. Product was purified on flash column chromatography (2–15% of MeOH in DCM) giving compound **11r** (124 mg, 93%). **^{1}H NMR** (400 MHz, DMSO-d_6) δ 8.20 (d, J = 2.3 Hz, 1H), 8.18 (s, 1H), 8.10 (dd, J = 9.0, 2.3 Hz, 1H), 8.04 (s, 1H), 7.91 (d, J = 8.4 Hz, 2H), 7.66 (d, J = 8.4 Hz, 2H), 7.49 (d, J = 9.1 Hz, 1H), 7.46 (s, 1H), 6.76 (s, 2H), 6.12 (d, J = 7.3 Hz, 1H), 4.83 (dd, J = 7.3, 4.5 Hz, 2H), 4.32 (d, J = 4.5 Hz, 1H), 4.13–3.99 (m + s, 4H), 3.74 (dd, J = 14.3, 6.6 Hz, 1H), 3.17–3.07 (m, 1H), 2.75 (s, 3H), 0.94 (s, 9H), 0.69 (s, 9H), 0.16 (s, 3H), 0.13 (s, 3H), -0.08 (s, 3H), -0.39 (s, 3H).**^{13}C NMR** (101 MHz, DMSO-d_6) δ 167.3, 163.9, 157.8, 153.1, 150.5, 134.4, 133.9, 133.0, 131.1, 129.9, 128.3, 127.9, 125.4, 115.3, 113.4, 102.4, 101.6, 95.1, 91.0, 87.1, 85.1, 83.7, 73.7, 73.4, 57.4, 51.9, 36.5, 25.9, 25.6, 17.9, 17.6, -4.5, -4.6 (2C), -5.3. **LRMS** (ESI) [M + H]$^+$ m/z: 846.2.

***N*-(((2*R*,3*R*,4*R*,5*R*)-5-(4-Amino-5-(pyridin-3-ylethynyl)-7*H*-pyrrolo[2,3-*d*]pyrimidin-7-yl)-3,4-bis((*tert*-butyldimethylsilyl)oxy)tetrahydrofuran-2-yl)methyl)-3-cyano-4-methoxy-*N*-methylbenzenesulfonamide (11s)**

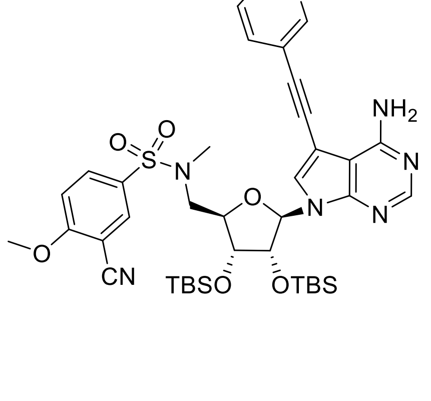

Compound **11s** was prepared using General procedure starting from intermediate **10** (130 mg, 0.157 mmol) and 3-ethynylpyridine[12]. Product was purified on flash column chromatography (2–15% of EtOH in DCM) followed by reverse phase flash column chromatography (50–100% ACN in water, 0.1% of FA as modifier) providing **11s** (90 mg, 71%). **^{1}H NMR** (400 MHz, DMSO-d_6) δ 8.78 (dd, J = 2.2, 0.9 Hz, 1H), 8.57 (dd, J = 4.8, 1.7 Hz, 1H), 8.20 (d, J =

2.4 Hz, 1H), 8.17 (s, 1H), 8.10 (dd, J = 9.0, 2.4 Hz, 1H), 8.05 (s, 1H), 8.01 (ddd, J = 7.9, 2.2, 1.7 Hz, 1H), 7.49 (d, J = 9.1 Hz, 1H), 7.45 (ddd, J = 8.0, 4.9, 0.9 Hz, 1H), 6.80 (s, 2H), 6.12 (d, J = 7.4 Hz, 1H), 4.84 (dd, J = 7.4, 4.5 Hz, 1H), 4.32 (d, J = 5.3 Hz, 1H), 4.03 (m + s, 4H), 3.74 (dd, J = 14.4, 6.8 Hz, 1H), 3.13 (dt, J = 14.3, 6.7 Hz, 1H), 2.75 (s, 3H), 0.94 (s, 9H), 0.70 (s, 9H), 0.16 (s, 3H), 0.13 (s, 3H), -0.08 (s, 3H), -0.39 (s, 3H). **^{13}C NMR** (101 MHz, DMSO-d_6) δ 163.9, 163.3, 157.7, 153.1, 151.7, 150.5, 148.8, 138.5, 134.4, 133.0, 129.9, 128.4, 123.7, 119.9, 115.3, 113.4, 102.3, 101.6, 94.9, 88.3, 87.1, 86.2, 83.7, 73.7, 73.4, 57.4, 51.9, 36.5, 25.9, 25.6, 17.9, 17.6, -4.5, -4.6 (2C), -5.3. **LRMS** (ESI) $[M + H]^+$ m/z: 804.2.

2.4 Final deprotection

General procedure B: Final deprotection of adenosine nucleoside derivatives

Protected alkylated sulfonamide was dissolved in 50% formic acid (3 mL) and stirred at RT for 1 to 3 days. After the deprotection was complete, solvent was evaporated and the residue was co-evaporated with 99% ethanol three times. Crude product was subjected to flash column chromatography.

***N*-(((2*R*,3*S*,4*R*,5*R*)-5-(6-Amino-9*H*-purin-9-yl)-3,4-dihydroxytetrahydrofuran-2-yl)methyl)-3-cyano-*N*-ethyl-4-methoxybenzenesulfonamide (3a) (See ref. [14])**

***N*-(((2*R*,3*S*,4*R*,5*R*)-5-(6-Amino-9*H*-purin-9-yl)-3,4-dihydroxytetrahydrofuran-2-yl)methyl)-3-cyano-4-methoxy-*N*-methylbenzenesulfonamide (3b)**

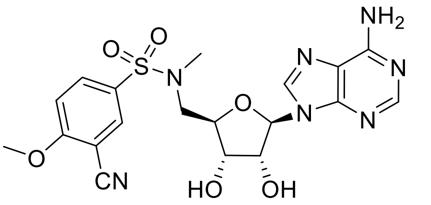

Compound **3b** was prepared following General procedure B starting from **2b** (19 mg, 0.037 mmol). Crude product was subjected to flash column chromatography (2–20% of MeOH in DCM) giving **3b** (17 mg, 97%). **^{1}H NMR** (400 MHz, DMSO-d_6) δ 8.35 (s, 1H), 8.17 (d, J = 2.2 Hz, 1H), 8.14 (s, 1H), 8.03 (dd, J = 9.0, 2.2 Hz, 1H), 7.41 (d, J = 9.1 Hz, 1H), 7.29 (s, 2H), 5.88 (d, J = 5.9 Hz, 1H), 5.53 (d, J = 5.8 Hz, 1H), 5.36 (d, J = 4.5 Hz, 1H), 4.75 (m, 1H), 4.19 (m, 2H), 4.06 (m, 1H), 4.01 (s, 3H), 3.52 (dd, J = 14.2, 4.8 Hz, 1H), 3.17 (dd, J = 14.1, 7.5 Hz, 1H), 2.69 (s, 3H). **^{13}C NMR** (101MHz, DMSO-d_6) δ 163.9, 156.3, 152.8, 149.5, 140.2, 134.4, 133.2, 129.7, 119.5, 115.2, 113.3, 101.5, 87.8, 82.7, 72.4, 71.5, 57.4, 52.1, 36.3. **HRMS** (ESI) Calculated for $C_{19}H_{22}N_7O_6S$, $[M + H]^+$ m/z: 476.1347, $[M + H]^+$, found 476.1343.

***N*-(((2*R*,3*S*,4*R*,5*R*)-5-(6-Amino-9*H*-purin-9-yl)-3,4-dihydroxytetrahydrofuran-2-yl)methyl)-3-cyano-*N*-isopropyl-4-methoxybenzenesulfonamide (3c)**

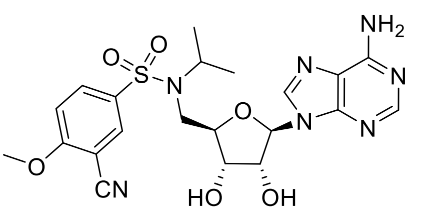

Final compound **3c** was prepared following General procedure B starting from **2c** (32 mg, 0.059 mmol). Crude product was subjected to flash column chromatography (2–20% of MeOH in DCM) giving **3c** (25 mg, 84%). **^{1}H NMR** (500 MHz, DMSO-d_6) δ 8.38 (s, 1H), 8.20 (d, J = 2.4 Hz, 1H), 8.16 (s, 1H), 8.08 (dd, J = 9.0, 2.4 Hz, 1H), 7.39 (d, J = 9.1 Hz, 1H), 7.28 (s, 2H), 5.89 (d, J = 6.2 Hz, 1H), 5.49 (d, J = 6.2 Hz, 1H), 5.35 (d, J = 4.8 Hz, 1H), 4.89–4.82 (m, 1H), 4.28–4.22 (m, 1H), 4.17 (ddd, J = 8.1, 5.3, 3.2 Hz, 2H), 4.01 (s, 3H), 3.92 (hept, J = 6.7 Hz, 1H), 3.68 (dd, J = 15.4, 5.3 Hz, 1H), 3.32 (m, 1H), 0.93 (d, J = 6.7 Hz, 3H), 0.88 (d, J = 6.7 Hz, 3H). **^{13}C NMR** (126 MHz, DMSO-d_6) δ 163.7, 156.3, 152.7,

149.5, 140.6, 134.0, 132.9, 132.8, 119.6, 115.2, 113.3, 101.3, 87.9, 84.3, 72.3, 71.7, 57.3, 49.8, 45.7, 21.5, 19.8. **HRMS** (ESI) Calculated for $C_{21}H_{26}N_7O_6S$, $[M + H]^+$ m/z: 503.1660, $[M + H]^+$, found 504.1657.

N-(((2*R*,3*S*,4*R*,5*R*)-5-(6-Amino-9*H*-purin-9-yl)-3,4-dihydroxytetrahydrofuran-2-yl)methyl)-3-cyano-*N*-cyclopentyl-4-methoxybenzenesulfonamide (3d)

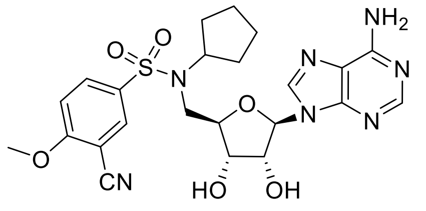

Adenosine analogue **3d** was prepared following General procedure B starting from **2d** (19 mg, 0.037 mmol). Crude product was subjected to reverse phase column chromatography (10–50% of ACN in water) giving **3d** (24 mg, 83%). **^{1}H NMR** (400 MHz, DMSO-*d6*) δ 8.36 (s, 1H), 8.23 (d, *J* = 2.2 Hz, 1H), 8.14 (s, 1H), 8.10 (dd, *J* = 9.0, 2.2 Hz, 1H), 7.40 (d, *J* = 9.1 Hz, 1H), 7.29 (s, 2H), 5.89 (d, *J* = 6.1 Hz, 1H), 5.50 (bs, 1H), 5.38 (bs, 1H), 4.91 (t, *J* = 5.4 Hz, 1H), 4.33–4.25 (m, 1H), 4.24–4.14 (m, 1H), 4.09–3.94 (m, 4H), 3.63 (dd, *J* = 15.4, 4.6 Hz, 1H), 3.23 (dd, *J* = 15.6, 7.8 Hz, 1H), 1.57–1.08 (m, 8H). **^{13}C NMR** (101MHz, DMSO-*d6*) δ 163.8, 156.3, 152.6, 149.5, 140.8, 134.3, 133.1, 132.2, 119.7, 115.3, 113.3, 101.4, 88.0, 84.5, 72.1, 71.7, 59.1, 57.3, 46.8, 29.4, 27.8, 23.3, 22.8. **HRMS** (ESI) Calculated for $C_{26}H_{28}N_7O_6S$, $[M + H]^+$ m/z: 530.1816, $[M + H]^+$, found 530.1812.

General procedure C: Final deprotection of 7-deazaadenosine-based compounds using TFA

Starting bis(silyl) protected alkylated sulfonamide was dissolved in TFA/water (9:1) mixture and was stirred at RT for 10–14 h. After the reaction was completed the solvent was evaporated and the residue was co-distilled with methanol 3–4 times. The residue was subjected to reverse phase flash column chromatography.

General procedure D: Final deprotection of 7-deazaadenosine-based compounds using TEA·3HF

Starting bis(silyl) protected alkylated sulfonamide was dissolved in anhydrous ACN/DMSO (2:1) mixture or pure DMSO, respectively. TEA·3HF (6 eq.) was added to the solution and the resulting mixture was stirred at RT–50 °C for 1–3 days. After the reaction was completed ACN was evaporated and the residue was subjected to reverse phase flash column chromatography.

N-(((2*R*,3*S*,4*R*,5*R*)-5-(5-((1*H*-Benzo[*d*]imidazol-2-yl)ethynyl)-4-amino-7*H*-pyrrolo[2,3-*d*]pyrimidin-7-yl)-3,4-dihydroxytetrahydrofuran-2-yl)methyl)-3-cyano-*N*-ethyl-4-methoxybenzenesulfonamide (12a)

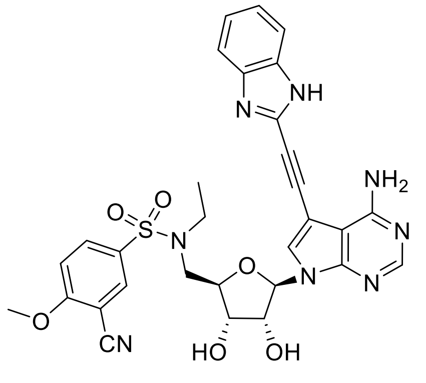

Nucleoside analogue **12a** was prepared following General procedure C starting from **11a$_{Boc}$** (95 mg, 0.10 mmol). Crude product was subjected to reverse phase flash column chromatography (10–70% of ACN in water, 0.1% of FA as modifier) giving **12a** (39 mg, 63%). **^{1}H NMR** (400 MHz, DMSO-*d6*) δ 13.19 (s, 1H), 8.22–8.18 (m, 2H), 8.13 (s, 1H), 8.08 (dd, *J* = 9.0, 2.4 Hz, 1H), 7.66–7.53 (m, 2H), 7.36 (d, *J* = 9.1 Hz, 1H), 7.30–7.21 (m, 2H), 6.92 (s, 2H), 6.09 (d, *J* = 6.0 Hz, 1H), 5.51 (d, *J* = 6.1 Hz, 1H), 5.37 (d, *J* = 4.9 Hz, 1H), 4.55 (q, *J* = 5.7 Hz, 1H), 4.12 (q, *J* = 4.5 Hz, 1H), 4.03

(dt, J = 8.3, 4.4 Hz, 1H), 3.99 (s, 3H), 3.64 (dd, J = 14.7, 4.7 Hz, 1H), 3.41–3.34 (m, 1H), 3.25 (dt, J = 14.2, 7.1 Hz, 1H), 3.15 (dt, J = 14.4, 7.0 Hz, 1H), 0.99 (t, J = 7.1 Hz, 3H). **^{13}C NMR** (101MHz, DMSO-d_6) δ 163.6, 157.6, 153.3, 150.4, 143.3, 135.4, 134.1, 133.7, 132.9, 132.2, 129.3, 123.7, 122.4, 119.1, 115.2, 113.1, 111.5, 102.1, 101.3, 93.5, 87.5, 85.1, 83.1, 82.6, 73.1, 71.4, 57.3, 50.0, 43.6, 13.9. **HRMS** (ESI) Calculated for $C_{30}H_{29}N_8O_6S$, [M + H]$^+$ m/z: 629.1925, [M + H]$^+$, found 629.1920.

_N_-(((2_R_,3_S_,4_R_,5_R_)-5-(5-((1_H_-Imidazol-2-yl)ethynyl)-4-amino-7_H_-pyrrolo[2,3-_d_]pyrimidin-7-yl)-3,4-dihydroxytetrahydrofuran-2-yl)methyl)-3-cyano-_N_-ethyl-4-methoxybenzenesulfonamide (12b)

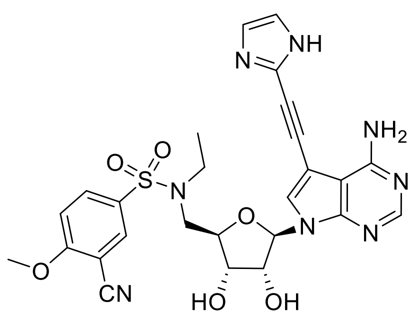

Compound **12b** was prepared following General procedure C starting from **11b$_{Boc}$** (126 mg, 0.10 mmol). Crude product was subjected to reverse phase flash column chromatography (10–70% of ACN in water, 0.1% of FA as modifier) providing **12b** (62 mg, 77%). **^{1}H NMR** (400 MHz, DMSO-d_6) δ 12.85 (s, 1H), 8.20 (d, J = 2.3 Hz, 1H), 8.17 (s, 1H), 8.07 (dd, J = 9.0, 2.4 Hz, 1H), 7.99 (s, 1H), 7.35 (d, J = 9.1 Hz, 1H), 7.29–7.26 (m, 1H), 7.05–7.00 (m, 1H), 6.79 (bs, 2H), 6.06 (d, J = 6.0 Hz, 1H), 5.47 (d, J = 6.2 Hz, 1H), 5.33 (d, J = 5.0 Hz, 1H), 4.52 (q, J = 5.9 Hz, 1H), 4.11 (q, J = 4.9 Hz, 2H), 4.06–3.98 (m + s, 4H), 3.62 (dd, J = 14.7, 4.7 Hz, 1H), 3.40–3.29 (m, 1H), 3.24 (dt, J = 14.2, 7.1 Hz, 1H), 3.12 (dq, J = 14.1, 7.0 Hz, 1H), 0.98 (t, J = 7.1 Hz, 3H). **^{13}C NMR** (101MHz, DMSO-d_6) δ 163.7, 157.7, 153.2, 150.3, 134.1, 132.9, 132.2, 129.6, 129.4, 128.0, 118.2, 115.2, 113.1, 102.2, 101.3, 94.2, 87.4, 83.2, 82.6, 82.2, 73.0, 71.5, 57.3, 50.0, 43.6, 13.9. **HRMS** (ESI) Calculated for $C_{26}H_{27}N_8O_6S$, [M + H]$^+$ m/z: 579.1769, [M + H]$^+$, found 579.1765.

_N_-(((2_R_,3_S_,4_R_,5_R_)-5-(4-Amino-5-(phenylethynyl)-7_H_-pyrrolo[2,3-_d_]pyrimidin-7-yl)-3,4-dihydroxytetrahydrofuran-2-yl)methyl)-3-cyano-_N_-ethyl-4-methoxybenzenesulfonamide (12c)

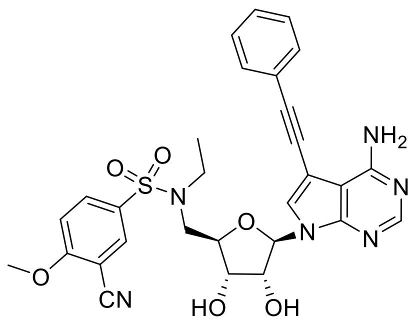

Preparation of **12c** was prepared followed modified General procedure D starting from **11c** (103 mg, 0.126 mmol) and performing the reaction in ACN/DMSO mixture (2:1). Crude product was subjected to flash column chromatography (10–50% of acetone in DCM) affording **11d** (58 mg, 78%). **^{1}H NMR** (400 MHz, DMSO-d_6) δ 8.20 (d, J = 2.4 Hz, 1H), 8.17 (s, 1H), 8.06 (dd, J = 9.0, 2.4 Hz, 1H), 7.94 (s, 1H), 7.64–7.53 (m, 2H), 7.48–7.39 (m, 3H), 7.34 (d, J = 9.1 Hz, 1H), 6.73 (bs, 2H), 6.06 (d, J = 6.1 Hz, 1H), 5.46 (d, J = 6.2 Hz, 1H), 5.33 (d, J = 5.0 Hz, 1H), 4.51 (q, J = 6.0 Hz, 1H), 4.11 (q, J = 4.9 Hz, 2H), 4.04–3.85 (m + s, 4H), 3.63 (dd, J = 14.7, 4.9 Hz, 1H), 3.36–3.29 (m, 1H), 3.24 (dt, J = 14.2, 7.1 Hz, 1H), 3.13 (dq, J = 14.1, 7.0 Hz, 1H), 0.99 (t, J = 7.1 Hz, 3H). **^{13}C NMR** (101MHz, DMSO-d_6) δ 163.6, 157.8, 153.1, 150.3, 134.1, 132.9, 132.2, 131.3, 128.9, 128.7, 127.5, 122.7, 115.2, 113.1, 102.3, 101.3, 95.2, 91.4, 87.3, 83.1, 82.5, 73.0, 71.5, 57.3, 50.0, 43.6, 14.0. **HRMS** (ESI) Calculated for $C_{29}H_{29}N_6O_6S$, [M + H]$^+$ m/z: 589.1864, [M + H]$^+$, found 589.1866.

N-(((2R,3S,4R,5R)-5-(4-Amino-5-(naphthalen-1-ylethynyl)-7H-pyrrolo[2,3-d]pyrimidin-7-yl)-3,4-dihydroxytetrahydrofuran-2-yl)methyl)-3-cyano-N-ethyl-4-methoxybenzenesulfonamide (12d)

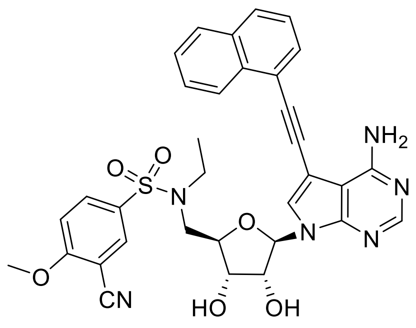

Analogue **12d** was prepared following General procedure D starting from **11d** (97 mg, 0.11 mmol) and performing the reaction in ACN/DMSO mixture (2:1). Crude product was subjected to reverse phase flash column chromatography (10–90% of ACN in water) providing **12d** (63 mg, 88%). **^{1}H NMR** (400 MHz, DMSO-d_6) δ 8.38–8.31 (m, 1H), 8.22 (d, J = 2.4 Hz, 1H), 8.20 (s, 1H), 8.10 (s, 1H), 8.08 (dd, J = 9.0, 2.4 Hz, 1H), 8.04–7.97 (m, 2H), 7.89 (dd, J = 7.1, 0.9 Hz, 1H), 7.70–7.57 (m, 2H), 7.59–7.54 (m, 1H), 7.34 (d, J = 9.1 Hz, 1H), 6.80 (bs, 2H), 6.10 (d, J = 6.1 Hz, 1H), 5.50 (d, J = 6.2 Hz, 1H), 5.35 (d, J = 5.0 Hz, 1H), 4.57 (q, J = 6.0 Hz, 1H), 4.14 (q, J = 5.0 Hz, 1H), 4.03 (dt, J = 8.1, 4.5 Hz, 1H), 3.98 (s, 3H), 3.64 (dd, J = 14.7, 4.8 Hz, 1H), 3.37 (dd, J = 14.7, 8.0 Hz, 1H), 3.26 (dt, J = 14.2, 7.1 Hz, 1H), 3.15 (dq, J = 14.1, 7.0 Hz, 1H), 1.01 (t, J = 7.1 Hz, 3H). **^{13}C NMR** (101MHz, DMSO-d_6) δ 163.6, 157.8, 150.4, 134.1, 133.0, 132.9, 132.5, 132.3, 130.4, 129.0, 128.7, 127.9, 127.4, 126.9, 125.8, 125.5, 120.1, 115.2, 113.1, 102.3, 101.3, 95.3, 89.3, 88.1, 87.3, 82.5, 73.1, 71.5, 57.3, 50.0, 43.6, 13.9. **HRMS** (ESI) Calculated for $C_{33}H_{31}N_6O_6S$, $[M + H]^+$ m/z: 639.2020, $[M + H]^+$, found 639.2017.

N-(((2R,3S,4R,5R)-5-(4-Amino-5-(quinolin-3-ylethynyl)-7H-pyrrolo[2,3-d]pyrimidin-7-yl)-3,4-dihydroxytetrahydrofuran-2-yl)methyl)-3-cyano-N-ethyl-4-methoxybenzenesulfonamide (12e)

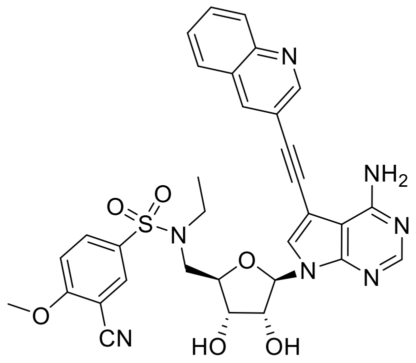

Compound **12e** was prepared following General procedure starting D from **11e** (99 mg, 0.11 mmol) and performing the reaction in ACN/DMSO mixture (2:1). Crude product was subjected to reverse phase flash column chromatography (25–100% of ACN in water, 0.1% of FA as modifier) affording product **12e** (39 mg, 54%). **^{1}H NMR** (400 MHz, DMSO-d_6) δ 9.04 (d, J = 2.0 Hz, 1H), 8.64 (d, J = 1.7 Hz, 1H), 8.21 (d, J = 2.3 Hz, 1H), 8.19 (s, 1H), 8.10–7.97 (m, 4H), 7.85–7.76 (m, 1H), 7.72–7.63 (m, 1H), 7.34 (d, J = 9.1 Hz, 1H), 6.87 (bs, 2H), 6.09 (d, J = 6.0 Hz, 1H), 5.48 (d, J = 6.2 Hz, 1H), 5.35 (d, J = 5.0 Hz, 1H), 4.54 (q, J = 5.9 Hz, 1H), 4.12 (q, J = 4.8 Hz, 1H), 4.07–3.93 (m + s, 4H), 3.64 (dd, J = 14.7, 4.8 Hz, 1H), 3.40–3.31 (m, 1H), 3.26 (dt, J = 14.1, 7.1 Hz, 1H), 3.14 (dq, J = 14.0, 6.9 Hz, 1H), 1.00 (t, J = 7.1 Hz, 3H). **^{13}C NMR** (101MHz, DMSO-d_6) δ 163.7, 157.8, 153.2, 151.8, 150.4, 146.3, 138.1, 134.1, 132.9, 132.2, 130.5, 129.0, 128.3, 128.1, 127.7, 127.0, 117.1, 115.2, 113.1, 102.2, 101.3, 94.9, 88.9, 87.4, 86.3, 82.6, 73.1, 71.5, 57.3, 50.0, 43.6, 14.0. **HRMS** (ESI) Calculated for $C_{32}H_{30}N_7O_6S$, $[M + H]^+$ m/z: 640.1973, $[M + H]^+$, found 640.1971.

***N*-(((2*R*,3*S*,4*R*,5*R*)-5-(4-Amino-5-((6-hydroxypyridin-3-yl)ethynyl)-7*H*-pyrrolo[2,3-*d*]pyrimidin-7-yl)-3,4-dihydroxytetrahydrofuran-2-yl)methyl)-3-cyano-*N*-ethyl-4-methoxybenzenesulfonamide (12f)**

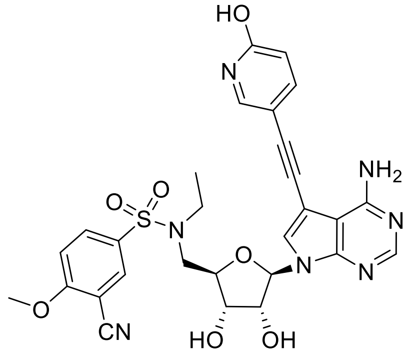

Preparation **of 12f** followed General procedure D starting from **11f** (48 mg, 0.058 mmol) performing the reaction in ACN/DMSO mixture. Crude product was subjected to reverse phase flash column chromatography (10–50% of ACN in water, 0.1% of FA as modifier) giving **12f** (21 mg, 60%). **^{1}H NMR** (400 MHz, DMSO-*d$_6$*) δ 11.96 (s, 1H), 8.20 (d, *J* = 2.4 Hz, 1H), 8.14 (s, 1H), 8.05 (dd, *J* = 9.0, 2.4 Hz, 1H), 7.84–7.79 (m, 2H), 7.55 (dd, *J* = 9.5, 2.5 Hz, 1H), 7.33 (d, *J* = 9.1 Hz, 1H), 6.72 (bs, 2H), 6.37 (d, *J* = 9.5 Hz, 1H), 6.04 (d, *J* = 6.0 Hz, 1H), 5.44 (d, *J* = 6.2 Hz, 1H), 5.32 (d, *J* = 5.1 Hz, 1H), 4.49 (q, *J* = 6.0 Hz, 1H), 4.09 (q, *J* = 4.9 Hz, 1H), 3.98 (m + s, 4H), 3.61 (dd, *J* = 14.7, 4.7 Hz, 1H), 3.37–3.28 (m, 1H), 3.24 (dt, *J* = 14.1, 7.2 Hz, 1H), 3.12 (dq, *J* = 14.1, 6.9 Hz, 1H), 0.98 (t, *J* = 7.1 Hz, 3H). **^{13}C NMR** (101MHz, DMSO-*d$_6$*) δ 163.7, 161.3, 157.7, 153.0, 150.2, 142.9, 139.9, 134.1, 132.9, 132.2, 127.1, 120.1, 115.2, 113.1, 102.2, 101.3, 95.5, 87.6, 87.2, 82.5, 82.4, 73.0, 71.5, 57.3, 50.0, 43.6, 14.0. Signal of one carbon atom was not observed. **HRMS** (ESI) Calculated for $C_{28}H_{28}N_7O_7S$, $[M + H]^+$ m/z: 606.1765, $[M + H]^+$, found 606.1766.

***N*-(((2*R*,3*S*,4*R*,5*R*)-5-(4-Amino-5-((2-hydroxypyrimidin-5-yl)ethynyl)-7*H*-pyrrolo[2,3-*d*]pyrimidin-7-yl)-3,4-dihydroxytetrahydrofuran-2-yl)methyl)-3-cyano-*N*-ethyl-4-methoxybenzenesulfonamide (12g)**

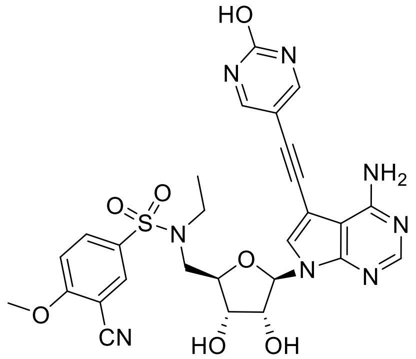

To the solution of compound **11g$_{PMB}$** (125 mg, 0.131 mmol) in anhydrous DCM (2 mL) was added TFA (80 µl, 1.05 mmol, 8 eq.) and the mixture was stirred at RT overnight. Volatiles were evaporated and the residue was dissolved in TFA/water (9:1) mixture and stirred at RT for 20 hours. Solvent was evaporated and the residue co-distilled with methanol thrice. Crude product was subjected to reverse phase flash column chromatography (25–60% of ACN in water, 0.1% of FA as modifier) providing **12g** (20 mg, 25%). **^{1}H NMR** (400 MHz, DMSO-*d$_6$*) δ 12.35 (s, 1H), 8.54 (s, 2H), 8.19 (d, *J* = 2.3 Hz, 1H), 8.14 (s, 1H), 8.05 (dd, *J* = 9.0, 2.4 Hz, 1H), 7.85 (s, 1H), 7.33 (d, *J* = 9.1 Hz, 1H), 6.76 (bs, 2H), 6.04 (d, *J* = 6.0 Hz, 1H), 5.44 (d, *J* = 5.6 Hz, 1H), 5.32 (d, *J* = 4.3 Hz, 1H), 4.53–4.45 (m, 1H), 4.15–4.05 (m, 1H), 3.98 (m + s, 4H), 3.61 (dd, *J* = 14.7, 4.7 Hz, 1H), 3.36–3.28 (m, 1H), 3.28–3.19 (m, 1H), 3.12 (dq, *J* = 14.1, 7.0 Hz, 1H), 0.98 (t, *J* = 7.1 Hz, 3H). **^{13}C NMR** (101MHz, DMSO-*d$_6$*) δ 163.7, 157.7, 153.1, 150.3, 134.1, 132.9, 132.2, 127.4, 115.2, 113.1, 102.1, 101.3, 95.1, 87.3, 85.0, 84.6, 82.5, 73.0, 71.5, 57.3, 50.0, 43.6, 14.0. Pyrimidone carbon atoms signals were not observed. **HRMS** (ESI) Calculated for $C_{27}H_{27}N_9O_7S$, $[M + H]^+$ m/z: 607.1718, $[M + H]^+$, found 607.1715.

***N*-(((2*R*,3*S*,4*R*,5*R*)-5-(4-Amino-5-((6-aminopyridin-3-yl)ethynyl)-7*H*-pyrrolo[2,3-*d*]pyrimidin-7-yl)-3,4-dihydroxytetrahydrofuran-2-yl)methyl)-3-cyano-*N*-ethyl-4-methoxybenzenesulfonamide (12h)**

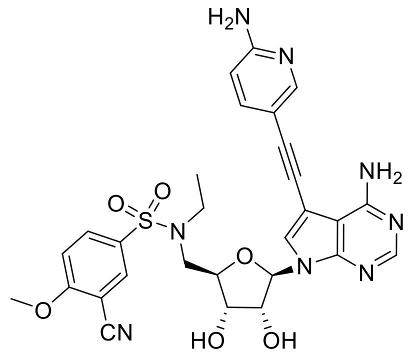

Compound **12h** was prepared following General procedure D starting from **11h** (69 mg, 0.083 mmol) performing the reaction in ACN/DMSO mixture. Crude product was subjected to reverse phase flash column chromatography (25–50% of ACN in water) affording **12h** (36 mg, 72%). **^{1}H NMR** (400 MHz, DMSO-*d6*) δ 8.20 (d, *J* = 2.3 Hz, 1H), 8.14 (s, 2H), 8.09–8.01 (m, 1H), 7.82 (s, 1H), 7.56–7.49 (m, 1H), 7.33 (d, *J* = 9.0 Hz, 1H), 6.66 (bs, 2H), 6.45 (d, *J* = 8.6 Hz, 1H), 6.39 (s, 2H), 6.04 (d, *J* = 5.9 Hz, 1H), 5.47 (s, 1H), 5.35 (s, 1H), 4.52–4.44 (m, 1H), 4.11–4.07 (m, 1H), 4.03–3.90 (m, 4H), 3.61 (dd, *J* = 14.7, 4.5 Hz, 1H), 3.33–3.7 (m, 1H)*, 3.27–3.20 (m, 1H), 3.12 (m, 1H), 0.98 (t, *J* = 6.9 Hz, 3H). **^{13}C NMR** (101MHz, DMSO-*d6*) δ 163.7, 159.3, 157.8, 153.0, 151.2, 150.2, 139.5, 134.1, 132.9, 132.2, 126.5, 115.2, 113.1, 107.7, 106.5, 102.3, 101.3, 95.9, 90.0, 87.2, 82.5, 82.3, 73.0, 71.5, 57.3, 50.0, 43.6, 14.0. **HRMS** (ESI) Calculated for $C_{28}H_{29}N_8O_6S$, $[M + H]^+$ m/z: 605.1925, $[M + H]^+$, found 605.1928.

***N*-(((2*R*,3*S*,4*R*,5*R*)-5-(4-Amino-5-((2-aminopyrimidin-5-yl)ethynyl)-7*H*-pyrrolo[2,3-*d*]pyrimidin-7-yl)-3,4-dihydroxytetrahydrofuran-2-yl)methyl)-3-cyano-*N*-ethyl-4-methoxybenzenesulfonamide (12i)**

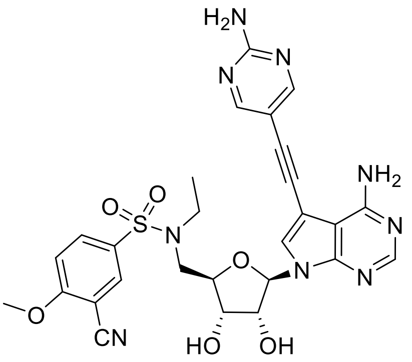

Compound **12i** was prepared following General procedure D starting from **11i** (63 mg, 0.076 mmol) performing the reaction in ACN/DMSO mixture. Crude product was subjected to reverse phase flash column chromatography (10–60% of ACN in water, 0.1% of FA as modifier) yielding **12i** (31 mg, 68%). **^{1}H NMR** (400 MHz, DMSO-*d6*) δ 8.46 (s, 2H), 8.20 (d, *J* = 2.4 Hz, 1H), 8.15 (s, 1H), 8.05 (dd, *J* = 9.0, 2.4 Hz, 1H), 7.85 (s, 1H), 7.33 (d, *J* = 9.1 Hz, 1H), 7.10 (s, 2H), 6.73 (s, 2H), 6.05 (d, *J* = 6.0 Hz, 1H), 5.44 (d, *J* = 6.2 Hz, 1H), 5.32 (d, *J* = 5.1 Hz, 1H), 4.49 (q, *J* = 5.9 Hz, 1H), 4.09 (q, *J* = 4.9 Hz, 1H), 3.98 (m + s, 4H), 3.62 (dd, *J* = 14.7, 4.8 Hz, 1H), 3.32 (m, 1H), 3.28–3.19 (m, 1H), 3.18–3.06 (m, 1H), 0.98 (t, *J* = 7.1 Hz, 3H). **^{13}C NMR** (101MHz, DMSO-*d6*) δ 163.7, 162.0, 160.4, 157.7, 153.0, 150.2, 134.1, 132.9, 132.2, 127.0, 115.2, 113.1, 106.4, 102.2, 101.3, 95.5, 87.2, 86.7, 85.1, 82.5, 73.0, 71.5, 57.3, 50.0, 43.6, 14.0. **HRMS** (ESI) Calculated for $C_{27}H_{28}N_9O_6S$, $[M + H]^+$ m/z: 606.1878, $[M + H]^+$, found 606.1880.

***N*-(((2*R*,3*S*,4*R*,5*R*)-5-(4-Amino-5-((2,4-dioxo-1,2,3,4-tetrahydropyrimidin-5-yl)ethynyl)-7*H*-pyrrolo[2,3-*d*]pyrimidin-7-yl)-3,4-dihydroxytetrahydrofuran-2-yl)methyl)-3-cyano-*N*-ethyl-4-methoxybenzenesulfonamide (12j)**

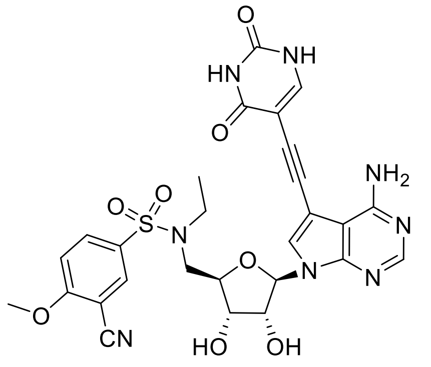

Nucleoside analogue **12j** was prepared following General procedure D starting from **11j** (52 mg, 0.061 mmol) performing the reaction in ACN/DMSO mixture. Crude product was subjected to reverse phase flash column chromatography (10–60% of ACN in water, 0.1% of FA as modifier) providing **12j** (34 mg, 89%). **^{1}H NMR** (400 MHz, DMSO-*d$_6$*) δ 11.57 (s, 1H), 11.39 (d, *J* = 4.8 Hz, 1H), 8.19 (d, *J* = 2.3 Hz, 1H), 8.11 (s, 1H), 8.05 (dd, *J* = 9.0, 2.4 Hz, 1H), 7.85 (d, *J* = 6.0 Hz, 1H), 7.71 (s, 1H), 7.33 (d, *J* = 9.1 Hz, 1H), 7.20 (bs, 2H), 6.02 (d, *J* = 6.1 Hz, 1H), 5.44 (d, *J* = 6.1 Hz, 1H), 5.30 (d, *J* = 4.8 Hz, 1H), 4.51 (q, *J* = 5.8 Hz, 1H), 4.13–4.05 (m, 1H), 3.99 (m + s, 4H), 3.61 (dd, *J* = 14.7, 4.7 Hz, 1H), 3.37–3.28 (m, 1H), 3.23 (dt, *J* = 14.2, 7.2 Hz, 1H), 3.11 (dq, *J* = 14.1, 7.0 Hz, 1H), 0.98 (t, *J* = 7.1 Hz, 3H). **^{13}C NMR** (101MHz, DMSO-*d$_6$*) δ 164.0, 163.7, 157.8, 153.1, 150.5, 150.2, 143.7, 134.1, 132.9, 132.3, 125.0, 115.2, 113.1, 102.5, 101.3, 97.3, 95.6, 87.4, 87.1, 84.9, 82.5, 72.9, 71.5, 57.3, 50.0, 43.6, 14.0. **HRMS** (ESI) Calculated for $C_{27}H_{27}N_8O_8S$, $[M + H]^+$ m/z: 623.1667, $[M + H]^+$, found 623.1666.

***N*-(((2*R*,3*S*,4*R*,5*R*)-5-(4-Amino-5-((4-amino-2-oxo-1,2-dihydropyrimidin-5-yl)ethynyl)-7*H*-pyrrolo[2,3-*d*]pyrimidin-7-yl)-3,4-dihydroxytetrahydrofuran-2-yl)methyl)-3-cyano-*N*-ethyl-4-methoxybenzenesulfonamide (12k)**

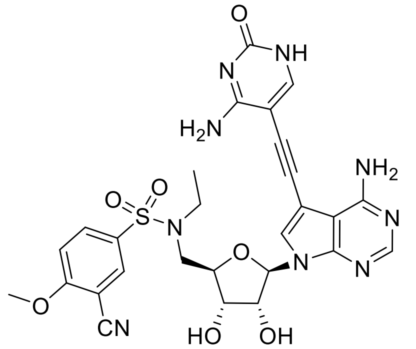

Deprotected nucleoside derivative **12k** was prepared following General procedure D starting from **11k** (26 mg, 0.031 mmol) performing the reaction in ACN/DMSO mixture. Crude product was subjected to reverse phase flash column chromatography (10–60% of ACN in water, 0.1% of FA as modifier) giving **12k** (14 mg, 74%). **^{1}H NMR** (400 MHz, DMSO-*d$_6$*) **^{1}H NMR** (400 MHz, DMSO-*d$_6$*) δ 10.97 (s, 1H), 8.22–8.15 (m, 1H), 8.13 (s, 1H), 8.11–8.01 (m, 1H), 7.92 (s, 1H), 7.84 (s, 1H), 7.62 (s, 1H), 7.33 (d, *J* = 8.9 Hz, 1H), 6.83 (s, 1H), 6.72 (s, 2H), 6.02 (d, *J* = 5.6 Hz, 1H), 5.46 (d, *J* = 6.0 Hz, 1H), 5.34 (d, *J* = 4.8 Hz, 1H), 4.49–4.40 (m, 1H), 4.14–4.05 (m, 1H), 4.03–3.92 (m, 4H), 3.66–3.57 (m, 1H), 3.36–3.24 (m, 1H)*, 3.30–3.18 (m, 1H)*, 3.18–3.05 (m, 1H), 0.98 (t, *J* = 6.8 Hz, 3H). **^{13}C NMR** (101MHz, DMSO-*d$_6$*) δ 165.2, 163.7, 157.8, 155.2, 152.9, 150.1, 147.1, 134.1, 132.9, 132.2, 127.6, 115.2, 113.2, 102.2, 101.3, 95.8, 88.7, 87.4, 87.2, 83.3, 82.4, 73.1, 71.5, 57.3, 49.9, 43.6, 13.9. **HRMS** (ESI) Calculated for $C_{27}H_{28}N_9O_7S$, $[M + H]^+$ m/z: 622.1827, $[M + H]^+$, found 622.1830.

N-(((2R,3S,4R,5R)-5-(4-Amino-5-(pyridin-3-ylethynyl)-7H-pyrrolo[2,3-d]pyrimidin-7-yl)-3,4-dihydroxytetrahydrofuran-2-yl)methyl)-3-cyano-N-ethyl-4-methoxybenzenesulfonamide (12l)

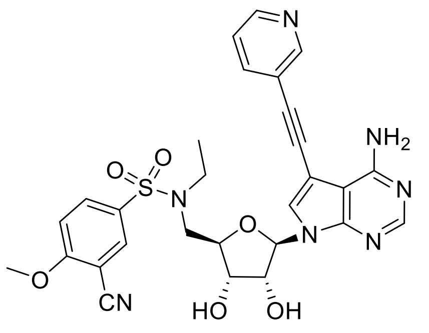

Compound **12l** was prepared following General procedure D starting from **11l** (67 mg, 0.082 mmol) and performing the reaction in DMSO. Crude product was subjected to reverse phase flash column chromatography (10–50% of ACN in water, 0.1% of FA as modifier) providing **12l** (46 mg, 95%). **^{1}H NMR** (400 MHz, DMSO-d_6) δ 8.79 (dd, J = 2.1, 0.8 Hz, 1H), 8.57 (dd, J = 4.8, 1.6 Hz, 1H), 8.20 (d, J = 2.4 Hz, 1H), 8.17 (s, 1H), 8.06 (dd, J = 9.0, 2.4 Hz, 1H), 8.01 (dt, J = 7.9, 1.8 Hz, 1H), 7.98 (s, 1H), 7.46 (ddd, J = 8.0, 4.9, 0.8 Hz, 1H), 7.34 (d, J = 9.1 Hz, 1H), 6.78 (bs, 2H), 6.06 (d, J = 6.0 Hz, 1H), 5.47 (d, J = 6.2 Hz, 1H), 5.34 (d, J = 5.0 Hz, 1H), 4.51 (q, J = 5.9 Hz, 1H), 4.11 (q, J = 4.9 Hz, 1H), 4.05–3.95 (m + s, 4H), 3.63 (dd, J = 14.8, 4.9 Hz, 1H), 3.32 (m, 1H)*, 3.24 (dt, J = 14.1, 7.1 Hz, 1H), 3.13 (dq, J = 14.0, 7.0 Hz, 1H), 0.99 (t, J = 7.1 Hz, 3H). **^{13}C NMR** (101MHz, DMSO-d_6) δ 163.7, 157.7, 153.2, 151.7, 150.4, 148.8, 138.5, 134.1, 132.9, 132.2, 128.2, 123.7, 120.0, 115.2, 113.1, 102.1, 101.3, 94.8, 88.2, 87.3, 86.3, 82.6, 73.0, 71.5, 57.3, 50.0, 43.6, 14.0. **HRMS** (ESI) Calculated for $C_{28}H_{28}N_7O_6S$, $[M + H]^+$ m/z: 590.1816, $[M + H]^+$, found 590.1815.

N-(((2R,3S,4R,5R)-5-(4-Amino-5-(pyrimidin-5-ylethynyl)-7H-pyrrolo[2,3-d]pyrimidin-7-yl)-3,4-dihydroxytetrahydrofuran-2-yl)methyl)-3-cyano-N-ethyl-4-methoxybenzenesulfonamide (12m)

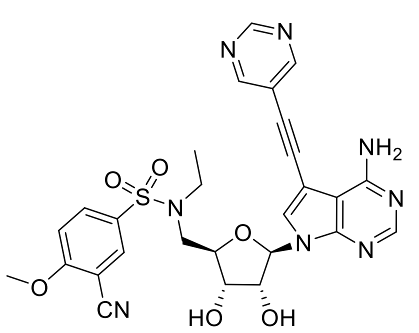

Compound **12m** was prepared following General procedure D starting from **11m** (73 mg, 0.089 mmol) performing the reaction in DMSO. Crude product was subjected to reverse phase flash column chromatography (10–60% of ACN in water, 0.1% of FA as modifier) furnishing product **12m** (45 mg, 86%). **^{1}H NMR** (400 MHz, DMSO-d_6) δ 9.17 (s, 1H), 9.03 (s, 2H), 8.20 (d, J = 2.4 Hz, 1H), 8.17 (s, 1H), 8.05 (dd, J = 9.0, 2.4 Hz, 1H), 8.01 (s, 1H), 7.33 (d, J = 9.1 Hz, 1H), 6.86 (bs, 2H), 6.07 (d, J = 6.0 Hz, 1H), 5.47 (d, J = 6.2 Hz, 1H), 5.34 (d, J = 5.1 Hz, 1H), 4.52 (q, J = 6.0 Hz, 1H), 4.10 (q, J = 4.9 Hz, 2H), 4.06–3.94 (m, 5H), 3.63 (dd, J = 14.7, 4.8 Hz, 1H), 3.38–3.29 (m, 1H)*, 3.25 (dt, J = 14.2, 7.1 Hz, 1H), 3.13 (dq, J = 14.3, 7.0 Hz, 1H), 0.99 (t, J = 7.1 Hz, 3H). **^{13}C NMR** (101MHz, DMSO-d_6) δ 163.7, 158.7, 157.7, 156.6, 153.2, 150.5, 134.1, 132.9, 132.2, 128.7, 119.6, 115.2, 113.1, 102.0, 101.3, 94.4, 89.7, 87.3, 85.0, 82.6, 73.0, 71.4, 57.3, 50.0, 43.6, 14.0. **HRMS** (ESI) Calculated for $C_{27}H_{27}N_8O_6S$, $[M + H]^+$ m/z: 591.1769, $[M + H]^+$, found 591.1770.

N-(((2R,3S,4R,5R)-5-(4-Amino-5-(pyridin-4-ylethynyl)-7H-pyrrolo[2,3-d]pyrimidin-7-yl)-3,4-dihydroxytetrahydrofuran-2-yl)methyl)-3-cyano-N-ethyl-4-methoxybenzenesulfonamide (12n)

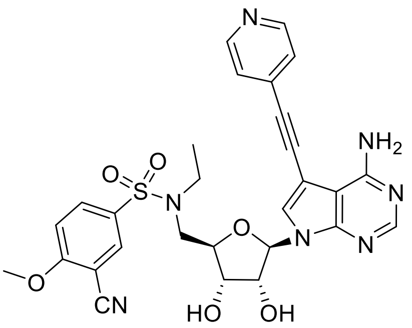

Preparation of **12n** followed General procedure C starting from **11n** (77 mg, 0.094 mmol). Crude product was subjected to reverse phase flash column chromatography (10–50% of ACN in water, 0.1% of FA as modifier) giving **12n** (44 mg, 80%). **^{1}H NMR** (400 MHz, DMSO-*d6*) δ 8.65–8.57 (m, 2H), 8.21 (d, *J* = 2.4 Hz, 1H), 8.18 (s, 1H), 8.10–8.00 (m, 2H), 7.59–7.52 (m, 2H), 7.34 (d, *J* = 9.1 Hz, 1H), 6.81 (bs, 2H), 6.07 (d, *J* = 6.1 Hz, 1H), 5.50 (d, *J* = 6.1 Hz, 1H), 5.37 (d, *J* = 4.9 Hz, 1H), 4.52 (q, *J* = 5.7 Hz, 1H), 4.11 (d, *J* = 3.9 Hz, 1H), 4.04–3.96 (m + s, 4H), 3.63 (dd, *J* = 14.8, 4.9 Hz, 1H), 3.37–3.29 (m, 1H), 3.24 (dt, *J* = 14.1, 7.1 Hz, 1H), 3.14 (dt, *J* = 14.4, 7.1 Hz, 1H), 0.99 (t, *J* = 7.1 Hz, 3H). **^{13}C NMR** (101MHz, DMSO-*d6*) δ 163.7, 157.7, 153.3, 150.5, 150.0, 134.1, 132.9, 132.2, 130.7, 129.0, 125.2, 115.2, 113.1, 102.1, 101.3, 94.4, 89.1, 87.9, 87.3, 82.6, 73.1, 71.5, 57.3, 50.0, 43.6, 14.0. **HRMS** (ESI) Calculated for $C_{28}H_{28}O_6N_7S$, $[M + H]^+$ m/z: 590.1816, $[M + H]^+$, found 590.1817.

4-((4-Amino-7-((2R,3R,4S,5R)-5-(((3-cyano-N-ethyl-4-methoxyphenyl)sulfonamido)methyl)-3,4-dihydroxytetrahydrofuran-2-yl)-7H-pyrrolo[2,3-d]pyrimidin-5-yl)ethynyl)benzoic acid (12o)

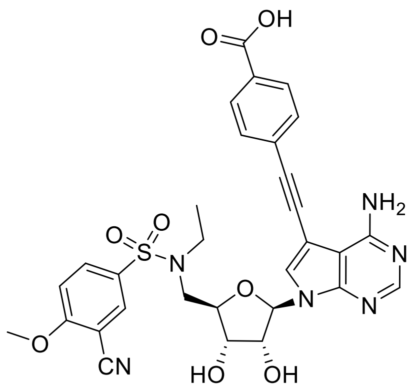

Analogue **12o** was prepared following General procedure D starting from **11o** (60 mg, 0.070 mmol) performing the reaction in DMSO. Crude product was subjected to reverse phase flash column chromatography (25–60% of ACN in water, 0.1% of FA as modifier) yielding **12o** (36 mg, 82%). **^{1}H NMR** (400 MHz, DMSO-*d6*) δ 13.12 (s, 1H), 8.21 (d, *J* = 2.4 Hz, 1H), 8.17 (s, 1H), 8.06 (dd, *J* = 9.0, 2.4 Hz, 1H), 8.00 (s, 1H), 7.98–7.94 (m, 2H), 7.74–7.66 (m, 2H), 7.34 (d, *J* = 9.1 Hz, 1H), 6.78 (bs, 2H), 6.07 (d, *J* = 6.0 Hz, 1H), 5.47 (bs, 1H), 5.34 (bs, 1H), 4.54–4.49 (m, 1H), 4.14–4.07 (m, 1H), 4.06–3.93 (m + s, 4H), 3.63 (dd, *J* = 14.8, 4.9 Hz, 1H), 3.38–3.29 (m, 1H), 3.24 (dt, *J* = 14.3, 7.2 Hz, 1H), 3.13 (dq, *J* = 14.2, 7.0 Hz, 1H), 0.99 (t, *J* = 7.1 Hz, 3H). **^{13}C NMR** (101MHz, DMSO-*d6*) δ 166.9, 163.7, 157.8, 153.2, 150.4, 134.1, 132.9, 132.2, 131.4, 130.3, 129.7, 128.3, 127.2, 115.2, 113.1, 102.2, 101.3, 94.9, 90.9, 87.3, 82.6, 73.1, 71.5, 57.3, 50.0, 43.6, 14.0. **HRMS** (ESI) Calculated for $C_{30}H_{29}N_6O_8S$, $[M + H]^+$ m/z: 633.1762, $[M + H]^+$, found 633.1761.

4-((4-Amino-7-((2*R*,3*R*,4*S*,5*R*)-5-(((3-cyano-*N*-ethyl-4-methoxyphenyl)sulfonamido)methyl)-3,4-dihydroxytetrahydrofuran-2-yl)-7*H*-pyrrolo[2,3-*d*]pyrimidin-5-yl)ethynyl)benzamide (12p)

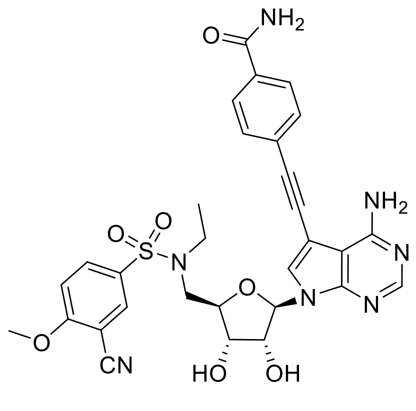

Compound **12p** was prepared following General procedure D starting from **11p** (76 mg, 0.088 mmol) performing the reaction in DMSO. Crude product was subjected to reverse phase flash column chromatography (25–60% of ACN in water, 0.1% of FA as modifier) affording product **12p** (41 mg, 74%). **^{1}H NMR** (400 MHz, DMSO-*d$_6$*) δ 8.21 (d, *J* = 2.4 Hz, 1H), 8.17 (s, 1H), 8.10–8.01 (m, 2H), 7.97 (s, 1H), 7.95–7.86 (m, 2H), 7.70–7.62 (m, 2H), 7.46 (s, 1H), 7.34 (d, *J* = 9.1 Hz, 1H), 6.77 (bs, 2H), 6.06 (d, *J* = 6.0 Hz, 1H), 5.46 (d, *J* = 6.2 Hz, 1H), 5.33 (d, *J* = 5.1 Hz, 1H), 4.52 (q, *J* = 6.0 Hz, 1H), 4.11 (q, *J* = 4.9 Hz, 1H), 4.05–3.94 (m +s , 4H), 3.63 (dd, *J* = 14.7, 4.8 Hz, 1H), 3.38–3.29 (m, 1H)*, 3.24 (dt, *J* = 14.2, 7.1 Hz, 1H), 3.13 (dq, *J* = 14.0, 7.0 Hz, 1H), 0.99 (t, *J* = 7.1 Hz, 3H). **^{13}C NMR** (101MHz, DMSO-*d$_6$*) δ 167.3, 163.7, 157.8, 153.2, 150.4, 134.1, 133.9, 132.9, 132.2, 131.1, 128.1, 127.9, 125.5, 115.2, 113.1, 102.2, 101.3, 95.0, 91.0, 87.3, 85.2, 82.6, 73.0, 71.5, 57.3, 50.0, 43.6, 14.0. **HRMS** (ESI) Calculated for $C_{30}H_{30}N_7O_7S$, $[M + H]^+$ m/z: 632.1922, $[M + H]^+$, found 632.1925.

***N*-(((2*R*,3*S*,4*R*,5*R*)-5-(4-Amino-5-(quinolin-3-ylethynyl)-7*H*-pyrrolo[2,3-*d*]pyrimidin-7-yl)-3,4-dihydroxytetrahydrofuran-2-yl)methyl)-3-cyano-4-methoxy-*N*-methylbenzenesulfonamide (12q)**

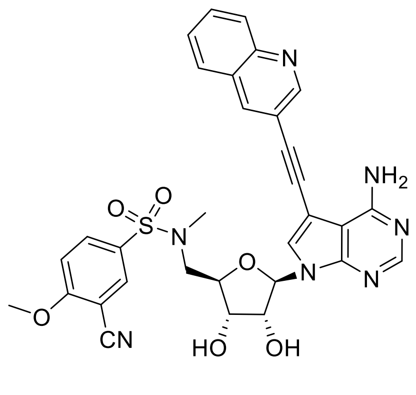

12q was prepared following General procedure C starting from **11q** (83 mg, 0.097 mmol). Crude product was subjected to reverse phase flash column chromatography (30–50% of ACN in water, 0.1% of FA as modifier) giving **12q** (43 mg, 71%). **^{1}H NMR** (400 MHz, DMSO-*d$_6$*) δ 9.04 (d, *J* = 2.1 Hz, 1H), 8.67–8.62 (m, 1H), 8.21 (d, *J* = 2.3 Hz, 1H), 8.18 (s, 1H), 8.10–7.97 (m, 4H), 7.81 (ddd, *J* = 8.4, 6.9, 1.5 Hz, 1H), 7.72–7.63 (m, 1H), 7.41 (d, *J* = 9.1 Hz, 1H), 6.86 (bs, 2H), 6.10 (d, *J* = 6.0 Hz, 1H), 5.53 (d, *J* = 6.1 Hz, 1H), 5.39 (d, *J* = 4.9 Hz, 1H), 4.51 (q, *J* = 5.9 Hz, 1H), 4.12 (q, *J* = 4.7 Hz, 1H), 4.06–3.96 (m + s, 4H), 3.50 (dd, *J* = 14.2, 4.9 Hz, 1H), 3.19 (dd, *J* = 14.2, 7.4 Hz, 1H), 2.72 (s, 3H). **^{13}C NMR** (101MHz, DMSO-*d$_6$*) δ 163.9, 157.8, 153.2, 151.8, 150.5, 146.3, 138.1, 134.4, 133.3, 130.5, 129.7, 129.0, 128.2, 127.7, 127.1, 117.1, 115.3, 113.3, 102.1, 101.5, 95.0, 88.9, 87.2, 86.3, 82.3, 73.1, 71.5, 57.3, 52.3, 36.3. Signal of one carbon atom was not observed. **HRMS** (ESI) Calculated for $C_{31}H_{28}N_7O_6S$, $[M + H]^+$ m/z: 626.1816, $[M + H]^+$, found 626.1818.

4-((4-Amino-7-((2*R*,3*R*,4*S*,5*R*)-5-(((3-cyano-4-methoxy-*N*-methylphenyl)sulfonamido)methyl)-3,4-dihydroxytetrahydrofuran-2-yl)-7*H*-pyrrolo[2,3-*d*]pyrimidin-5-yl)ethynyl)benzamide (12r)

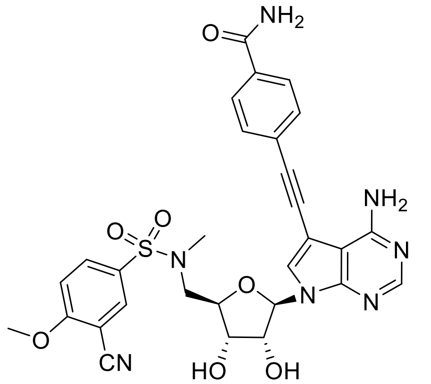

Compound **12r** was prepared following General procedure C starting from **11r** (116 mg, 0.137 mmol). Crude product was subjected to reverse phase flash column chromatography (25–50% of ACN in water, 0.1% of FA as modifier) providing **12r** (37 mg, 44%). **^{1}H NMR** (400 MHz, DMSO-*d$_6$*) δ 8.21 (d, *J* = 2.4 Hz, 1H), 8.17 (s, 1H), 8.10–8.00 (m, 2H), 7.96 (s, 1H), 7.94–7.87 (m, 2H), 7.70–7.61 (m, 2H), 7.46 (s, 1H), 7.41 (d, *J* = 9.1 Hz, 1H), 6.76 (s, 2H), 6.09 (d, *J* = 6.0 Hz, 1H), 5.48 (d, *J* = 6.2 Hz, 1H), 5.35 (d, *J* = 5.0 Hz, 1H), 4.49 (q, *J* = 5.9 Hz, 1H), 4.11 (q, *J* = 4.7 Hz, 2H), 4.06–3.95 (m, 4H), 3.49 (dd, *J* = 14.2, 5.0 Hz, 1H), 3.17 (dd, *J* = 14.1, 7.4 Hz, 1H), 2.71 (s, 3H). **^{13}C NMR** (101MHz, DMSO-*d$_6$*) δ 167.3, 163.9, 157.8, 153.2, 150.4, 134.4, 133.9, 133.3, 131.1, 129.7, 127.9, 125.5, 115.3, 113.3, 102.2, 101.5, 95.1, 91.0, 87.1, 85.2, 82.3, 73.1, 71.5, 57.4, 52.2, 36.3. Signal of one carbon atom was not observed. **HRMS** (ESI) Calculated for $C_{29}H_{28}N_7O_7S$, $[M + H]^+$ m/z: 618.1765, $[M + H]^+$, found 618.1765.

***N*-(((2*R*,3*S*,4*R*,5*R*)-5-(4-Amino-5-(pyridin-3-ylethynyl)-7*H*-pyrrolo[2,3-*d*]pyrimidin-7-yl)-3,4-dihydroxytetrahydrofuran-2-yl)methyl)-3-cyano-4-methoxy-*N*-methylbenzenesulfonamide (12s)**

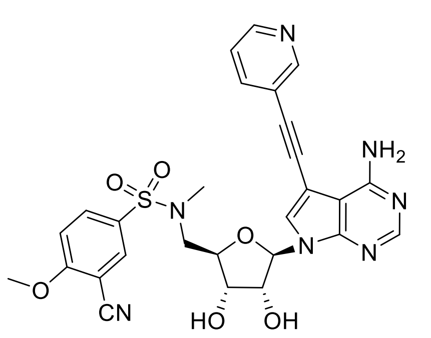

Compound **12s** was prepared following General procedure C starting from **11s** (82 mg, 0.097 mmol). Crude product was subjected to reverse phase flash column chromatography (25–50% of ACN in water, 0.1% of FA as modifier) giving **12s** (41 mg, 70%). **^{1}H NMR** (400 MHz, DMSO-*d$_6$*) δ 8.81–8.76 (m, 1H), 8.57 (dd, *J* = 4.8, 1.6 Hz, 1H), 8.20 (d, *J* = 2.3 Hz, 1H), 8.17 (s, 1H), 8.06 (dd, *J* = 9.0, 2.3 Hz, 1H), 8.01 (dt, *J* = 7.9, 1.8 Hz, 2H), 7.97 (s, 1H), 7.46 (dd, *J* = 7.3, 4.9 Hz, 1H), 7.41 (d, *J* = 9.1 Hz, 1H), 6.79 (bs, 2H), 6.09 (d, *J* = 6.0 Hz, 1H), 5.48 (d, *J* = 6.2 Hz, 1H), 5.35 (d, *J* = 5.0 Hz, 1H), 4.49 (q, *J* = 5.9 Hz, 1H), 4.11 (q, *J* = 4.8 Hz, 2H), 4.06–3.93 (m + s, 4H), 3.49 (dd, *J* = 14.2, 4.9 Hz, 1H), 3.17 (dd, *J* = 14.1, 7.4 Hz, 1H), 2.71 (s, 3H). **^{13}C NMR** (101MHz, DMSO-*d$_6$*) δ 164.2, 158.0, 153.5, 152.0, 150.7, 149.1, 138.8, 134.7, 133.5, 130.0, 128.3, 124.0, 120.3, 115.5, 113.6, 102.4, 101.8, 95.2, 88.5, 87.4, 86.5, 82.6, 73.4, 71.7, 57.6, 52.5, 36.6. **HRMS** (ESI) Calculated for $C_{27}H_{26}N_7O_6S$, $[M + H]^+$ m/z: 576.1660, $[M + H]^+$, found 576.1659.

3. Biochemical assays

3.1. Nsp14 Echo MS assay

For measuring inhibition of SARS-CoV-2 N^7-methyltransferase nsp14, we developed a robust system. The reactions were performed in 1536 well plates in a total volume of 4 µl per well. The reaction mixture contained substrates 4 µM *S*-Adenosyl-*L*-methionine (Sigma-Aldrich) and 4 µM GpppA in the reaction buffer (5 mM Tris pH 8.0, 1 mM TCEP, 0.1 mg/ml BSA, 0.005% Triton X-100, 1 mM $MgCl_2$). The reaction was initiated by adding 20 nM SARS-CoV-2 nsp14, mixed, spun, and incubated at 30 °C for 45 min. The reactions were stopped by adding 2 µl of 15% formic acid and analysed on an Echo mass spectrometry system coupled with a Sciex 6500 triple-quadrupole mass spectrometer operating with an electrospray ionization source. The rate of MTase activity was measured as the amount of the product of the reaction *S*-adenosylhomocysteine (SAH). The spectrometer was run in the multiple-reaction-monitoring (MRM) mode with the interface heated to 350 °C. The declustering potential was 20 V, the entrance potential 10 V, and the collision energy 28 eV. Ten nanolitres was injected in the mobile phase (flow rate of 0.40 ml/min; 100% methanol). The characteristic product ion of SAH, *m/z* 385.1 > 134.1, was used for quantification. For IC_{50} measuring, concentrations of tested compounds from 12.5 pM to 12.5 µM were used, and measured in duplicates. The compounds were shot to the cultivation plate using Echo 525 Acoustic Liquid Handler (Beckman Coulter) prior to the setting up the reaction. Dose-response curves were obtained by fitting the experimental peak area of S-adenosylhomocysteine (SAH) and different inhibitor concentration. The half maximal inhibitory concentration (IC_{50}) was calculated by variable slope model using Graph Pad 8.3.4 software using the equation: Y = Min + (Max-Min)/(1+10^((LogIC_{50}-X)*HillSlope)), where Max, Min are the upper and lower asymptotes.

3.2. Scintillation proximity selectivity assays

Selectivity assays were performed as previously described.[15] Briefly, the effect of **12q**, **12r** and **12s** on the methyltransferase activity of nsp14, SARS-CoV-2 nsp10-nsp16 complex, and 6 human methyltransferases (PRMT6, SUV39H1, SUV39H2, SETDB1, SETD7, and MLL1) were tested using scintillation proximity assay (SPA). The compounds were tested at 1 µM in duplicate. Assays were performed in 30 µL reaction volume for nsp14, 10 µL reaction volume for nsp10/16 complex and 20 µL reaction volume for the human methyltransferases. Nsp14 reactions contained 1.5 nM nsp14, 50 nM Biotin-RNA, and 250 nM ^{3}H-SAM (catalogue no. NET155 V250UC; PerkinElmer) in 20 mM Tris-HCl pH 7.5, 0.25 mM $MgCl_2$, 1 mM TCEP, and 0.01% Triton X-100. Nsp10-nsp16 reactions contained 100 nM nsp10-nsp16 complex, 0.8 µM Biotin-RNA, and 2 µM ^{3}H-SAM in 50 mM Tris-HCl pH 7.5, 1.5 mM $MgCl_2$, 1 mM TCEP, and 0.01% Triton X-100. Reactions for PRMT6, SUV39H1, SUV39H2, SETDB1, SETD7 and MLL1 contained ^{3}H-SAM and histone peptide substrate (Biotin-H3 (1-25 aa) or Biotin-H4 (1-24 aa)) at concentrations close to K_m values for each enzyme, and were assayed in 20 mM Tris-HCl pH 8.0, 5 mM DTT and 0.01% Triton X-100. Final DMSO concentration for all reactions was 5%. Reactions were incubated at 23 °C for 20 minutes for nsp14 and SETDB1, 30 minutes for nsp10-nsp16, and MLL1, and 1 hour for all other methyltransferases. Reactions were quenched with equal volume of 7.5 M GuHCl and topped up to 150 µL with 20 mM Tris-HCl pH 8.0 before transferring reaction mixtures to a 96-well OptiPlate (6005290, PerkinElmer) containing 50 µg Streptavidin PVT beads (RPNQ0006, PerkinElmer) per well. After overnight incubation, the plates were read using 2450 Microbeta2 (PerkinElmer) to measure

methyltransferase activity as counts per minute (CPM). The CPM measurements in the absence of compound (5% DMSO) for each enzyme was defined as 100% activity.

3.3. Intracellular Uptake

Steady-state cellular uptake was measured in CCRF-CEM cells (ATCC, CRL-119), VERO-E6 cells (ATCC, CRL-1586) and Calu-3 cells (HTB-55), respectively. Suspension cells (CCRF-CEM) were treated as follows: Prior to the experiment, cells were washed by centrifugation (250 x g, 5 min) in prewarmed (37 °C) serum-free RPMI medium and resuspended in the same vehicle to a concentration of 2 x 10^7 cells per mL. The cell suspension was distributed into the microtubes in 500 μL aliquots (10^7 cells), and the uptake process was initiated by the addition of 2.5 μL of the 10 mM DMSO solutions of the compounds (50 μM final conc.). Samples were incubated for 60 min at 37 °C in a CO_2 incubator using a rotary stirrer. The uptake was terminated by centrifugation at 900 x g for 5 min, at +4 °C. The medium was stored at −20 °C for later analysis of the extracellular concentration of the compounds. The cells were washed two times with 1 mL PBS (900 x g, 5 min, +4 °C), and also stored at −20 °C for subsequent analysis of the intracellular concentration of the compounds. At the day of the analysis, both media and the pelleted cells were quickly thawed. The cell pellets were extracted with 200 μL of ice-cold methanol by vigorous shaking, further diluted 1:1 with water and centrifuged at 20 000 x g for 10 min. As for the media samples, 10 μL aliquots were extracted with 990 μL of 50% methanol. The samples were then transferred to ECHO®-compatible 384-well plates and the amount of analytes were quantified by the ECHO-MS® System (Sciex) where acoustic droplet ejection module is coupled with Sciex 6500® triple quadrupole mass spectrometer operating with an electrospray ionization source. Spectrometer was run in the multiple reaction monitoring (MRM) mode. When necessary, the processed samples were further diluted in 50% methanol to fit in the calibration range. Calibration curves of the compounds in 50% methanol were measured alongside the samples within the same run. The ratio between the total compound concentration in the cells and compound concentration in the medium (K_p) at t=60 min was calculated according to: $K_p = (A_{cell} / V_{cell}) / C_{medium}$, where A_{cell} is the amount of compound in the cell lysate, V_{cell} is the CCRF-CEM cell volume (3.38 μL/10^7 cells)[16] and C_{medium} is the compound concentration in the medium sample after correcting for sample dilution. Experiments were performed in duplicates.

For adherent cells (Vero E6 and Calu-3), the workflow was modified as follows: Cells were seeded on 6-well plates at a concentration of 7.8 x 10^5 and 8.2 x 10^5 cells/well for Vero E6 and Calu-3, respectively, in 3 mL of serum-free culture media. The cells were left to attach overnight, the medium was then aspirated and replenished with 2 mL of fresh media containing the compounds (50 μM final conc). After 60 min at 37 °C the media was aspirated and the cells were washed twice with 1 mL of PBS. Then, 1 mL of fresh PBS was added and the cells were subjected to three freeze-thaw cycles and further diluted with 1:1 with MeOH. The cell lysates and the media were further processed as described above. Intracellular volume of Vero E6 and Calu-3 cells (8.6 and 14 μL/10^7 cells, respectively) was calculated from their diameter determined by cell counter (Luna FL) while in suspension (following the trypsinization) assuming the homogenous spheres.

3.4. Microsomal stability

Microsomal stability assay was performed using the 0.5 mg/ml human pooled liver microsomal preparation (Thermo Scientific) and 10 μM compounds in 90 mM TRIS-Cl buffer pH 7.4

containing 2 mM NADPH and 2 mM $MgCl_2$ for 10, 30 and 45 min at 37 °C. The reactions were terminated by the addition of four volumes of ice-cold methanol, mixed vigorously and left at -20 °C for 1 h. After that, the samples were centrifuged and the supernatants were analyzed by means of ECHO-MS® System (Sciex, Framingham, MA, USA). Zero time points were prepared by adding ice-cold methanol to the mixture of compound with cofactors prior to the addition of microsomes. The microsomal half-lives ($t_{1/2}$) were calculated using the equation $t_{1/2}=0.693/k$, where k is the slope found in the linear fit of the natural logarithm of the fraction remaining of the parent compound vs. incubation time. Intrinsic clearance (CL_{int}) was calculated using the following formula: $CL_{int} = V * \ln2 / t_{1/2}$, where V = incubation volume per milligram of microsomal protein (µL/mg) and $t_{1/2}$ = microsomal half-life.

3.5. Plasma stability

To determine plasma stability of the compounds, 5 µM of these were incubated with human pooled plasma from 50 donors (Biowest) for 120 min and 240 min at 37 °C. The reactions were terminated by adding four volumes of ice-cold methanol, the samples were then mixed vigorously and left at -20 °C for 1 h. After that, the samples were centrifuged, and the supernatant was analyzed by means of ECHO-MS® System (Sciex). Zero time points were prepared by adding ice-cold methanol to the compound prior the addition of the plasma.

3.6. Caco-2 permeability assay

Transepithelial bi-directional transport of tested compounds (5 µM, pH 7.4/7.4) across the Caco-2 monolayers was studied using the BD BioCoat HTS Caco-2 assay system (BD Biosciences, Bedford, MA) as described in detail elsewhere.[17] Aliquots were collected from the donor and acceptor compartment at the end of transport period (3 h), integrity of monolayers were verified using Lucifer Yellow dye and the compounds were quantified using LC-MS/MS (Sciex 6500® triple quadrupole). The apparent permeability coefficient (P_{app}) was calculated from the following equation: $P_{app} = (dQ / dt) / C_0 * A$, where dQ/dt is the rate of absorption of the drug across the cells, C_0 is the donor compartment concentration at time zero, and A is the area of the monolayer. Efflux ratio was expressed as (P_{app} B-A) / (P_{app} A-B). Recovery (%) was determined as (total compound mass in donor and receiver compartments at the end of the incubation / initial compound mass in the donor compartment) x 100.

4. Antiviral and cytotoxicity assays

The anti-SARS-CoV-2 activity was measured by determining the extent to which the test compounds inhibited virus-induced cytopathic effect (CPE) in Vero E6 (ECACC cat. no. 85020206) and Calu-3 (ATCC cat. no. HBT-55) cells. Two-fold serial dilutions of compounds were added in triplicate in a 384-well plate with 5 000 Vero E6 or 15 000 Calu-3 cells in DMEM medium with 2% FBS, 100 U of penicillin/ml and 100 μg of streptomycin/ml (all Merck). After 1 h incubation SARS-CoV-2 (strain hCoV-19/Czech Republic/NRL_6632_2/2020) was added at multiplicity of infection 0.03 IU/ml. Following three days incubation at 37 °C in 5% CO_2 the cell viability was determined by addition of XTT solution (Sigma-Aldrich) for 4 h and the absorbance of newly produced orange-colored formazan dye by viable cells was measured at 450 nm using EnVision plate reader (Perkin Elmer). Drug concentrations required to reduce viral cytopathic effect by 50% (EC_{50}) were calculated using nonlinear regression from plots of percentage cell viability versus $\log_{10}$ drug concentration using GraphPad Prism v.9.5.1 (GraphPad Software). Cytotoxicity was evaluated by incubating the same two-fold serial dilutions of each compound as above with Vero E6 and Calu-3 cells. Following three days incubation at 37 °C in 5% CO_2, the cell viability was determined by XTT assay as described above. The compound concentrations resulting in 50% reduction of absorbance (CC_{50}), which corresponds to 50% reduction of viable cells were calculated from plots of percentage of absorbance versus $\log_{10}$ drug concentration as above.

5. Docking studies

5.1. Docking in GOLD software

Docking in GOLD 2022.3.0 software[18] was done as in our previous work[8] using the nsp14 crystal structure (PDB ID: 7R2V chain A)[19] in complex with SAH instead of the homologous model. In brief, search space was defined by position of SAH (around 10 Å). SAH was also used as reference ligand. Docking experiments were performed using CHEMPLP scoring function (Diverse Solution Options set to–Cluster size = 10 and R.M.S.D. = 0.5 Å; Search efficiency 100%) with allowed flip ring corners option. The highest score for **12q** was 98.50 and this pose was used in the main text in Figure 4, A and B and in SI Figure S1, A-C.

5.2. Docking in Autodock Vina

Ligand preparation and docking experiments in Autodock Vina 1.1.2[20] and 1.2.3[21] were performed in similar fashion as in Mejdrová *et al.*[22, 23] In brief, docking was done using the standard docking procedure using above the mentioned crystal structure (PDB ID: 7R2V chain A)[19] without ligand. The grid box was centered at x=15.2; y=-11.9, z=-19.7, had dimensions: 18×20×24Å and exhaustiveness was set to 200. The results of the docking experiments for all compounds in the main series are shown in Table S5.

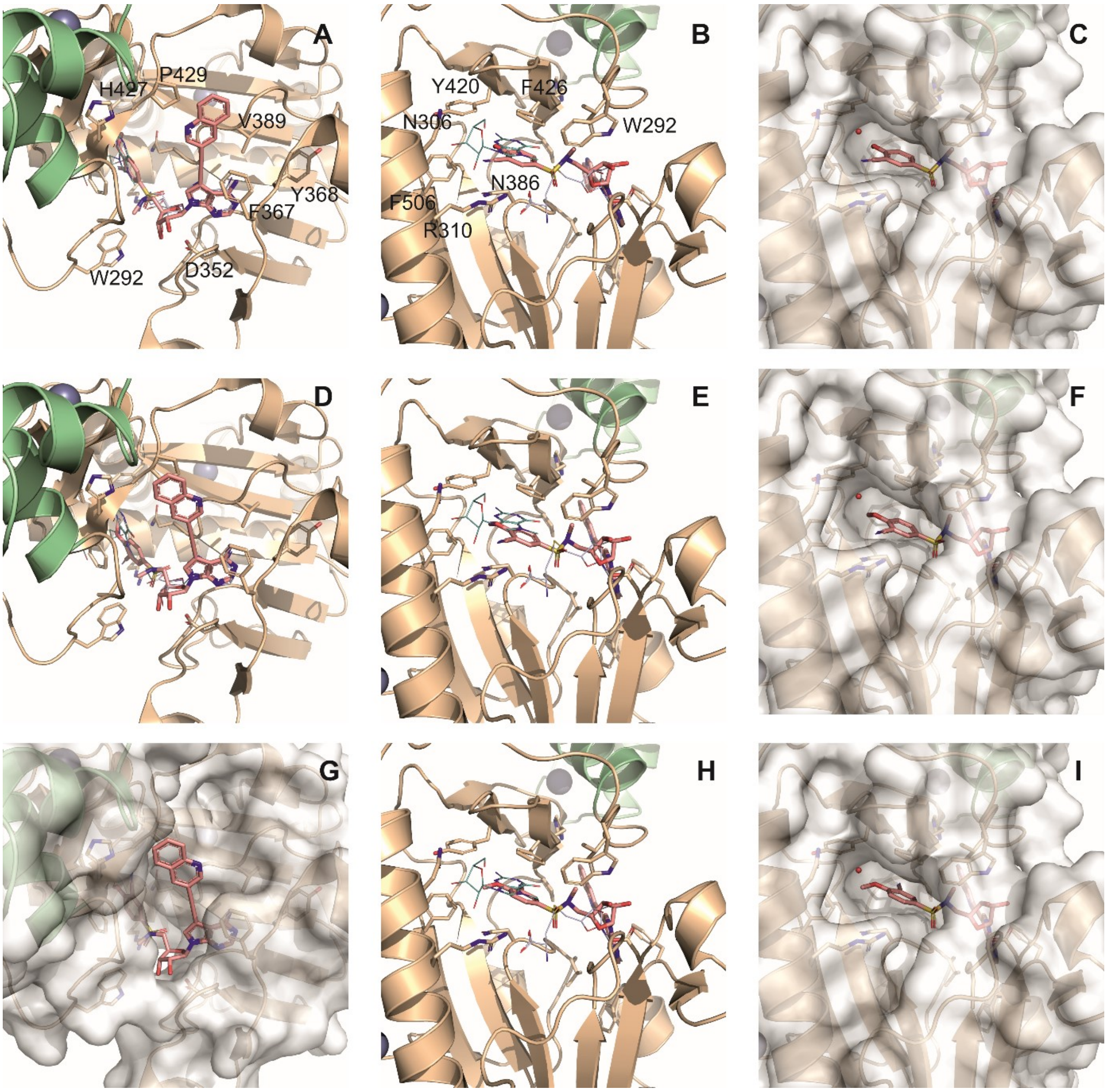

Figure S1. Comparison of docking experiments for compound **12q** using GOLD (A-C) and Autodock Vina (Pose A, D-F; Pose B, G-I). The ligand is represented as pink sticks in all cases. To aid in orientation within the SAM and RNA binding sites, we have included SAH (shown as blue-white wires) and the guanosine part of the RNA cap (shown as light teal wires) (A, B, D, E, H).

6. Supporting Tables

Table S1. Detailed reaction conditions and yields for Scheme 2.

Compd	Starting compd	Conditions step (g)	Yield of **11**	Conditions step (h)	Yield of **12**
**a**	**9**	$Pd(PPh_3)_4$/THF	62%	TFA/water (9:1)	63%
**b**	**9**	$Pd(PPh_3)_4$/THF	85%	TFA/water (9:1)	77%
**c**	**9**	$Pd(PPh_3)_4$/THF	95%	TEA·3HF	78%
**d**	**9**	$Pd(PPh_3)_4$/THF	83%	TEA·3HF	88%
**e**	**9**	$Pd(PPh_3)_4$/THF	86%	TEA·3HF	54%
**f**	**9**	$Pd(PPh_3)_4$/THF	60%	TEA·3HF	60%
**g**	**9**	$Pd(PPh_3)_4$/THF	96%	TFA/water (9:1)	25%
**h**	**9**	$Pd(PPh_3)_4$/THF	87%	TEA·3HF	72%
**i**	**9**	$Pd(PPh_3)_4$/THF	72%	TEA·3HF	68%
**j**	**9**	$Pd(PPh_3)_4$/DMF	56%	TEA·3HF	89%
**k**	**9**	$Pd(PPh_3)_2Cl_2$/DMF[a]	37%	TEA·3HF	74%
**l**	**9**	$Pd(PPh_3)_4$/THF	72%	TEA·3HF	92%
**m**	**9**	$Pd(PPh_3)_4$/THF	84%	TEA·3HF	86%
**n**	**9**	$Pd(PPh_3)_4$/THF	80%	TEA·3HF	79%
**o**	**9**	$Pd(PPh_3)_4$/DMF	62%	TEA·3HF	82%
**p**	**9**	$Pd(PPh_3)_4$/THF	78%	TEA·3HF	74%
**q**	**10**	$Pd(PPh_3)_4$/THF	77%	TFA/water (9:1)	71%
**r**	**10**	$Pd(PPh_3)_4$/THF	99%	TFA/water (9:1)	44%
**s**	**10**	$Pd(PPh_3)_4$/THF	76%	TFA/water (9:1)	70%

[a]Reaction was performed at 80 °C.

Table S2. Intracellular accumulation of selected compounds in CCRF-CEM, VERO-V6 and Calu-3 cells (60 min incubation, 50 μM concentration at t=0) and bi-directional permeability of the compounds through the differentiated Caco-2 cell monolayer.

	CCRF-CEM		
compound	EC (μM) ± sd	IC (μM) ± sd	K_p
**12a**	34 ± 0	6 407 ± 728	188
**12e**	0.2 ± 0.0	10 475 ± 1 451	63 149
**12h**	14 ± 1	6 666 ± 271	480
**12l**	29 ± 0	4 763 ± 397	163
**12o**	13 ± 3	9 164 ± 593	694
**12q**	0.6 ± 0.1	3 140 ± 348	5 325
**12r**	12 ± 6	10 708 ± 358	897
**12s**	15 ± 3	9 249 ± 1 390	604
	VERO-E6		
compound	EC (μM) ± sd	IC (μM) ± sd	K_p
**12a**	42 ± 3.2	34 509 ± 2 711	822
**12e**	16.5 ± 3.6	10 026 ± 3 369	608
**12h**	19.5 ± 2.0	17 255 ± 1 029	885
**12l**	17.1 ± 2.9	3 857 ± 331	225
**12o**	20.9 ± 0.8	5 176 ± 1 118	247
**12q**	0.1 ± 0.1	18 533 ± 7 709	211 805
**12r**	1.1 ± 0.5	19 777 ± 7 485	17 231
**12s**	2.6 ± 2.3	13 578 ± 3 626	5 208
	Calu-3		
compound	EC (μM) ± sd	IC (μM) ± sd	K_p
**12e**	5 ± 4	8 632 ± 993	1 766
**12q**	5.6 ± 5.7	89 640 ± 6 514	16 015
**12r**	4.0 ± 0.2	5 072 ± 974	1 254
**12s**	14.7 ± 0.8	1 557 ± 42	106

EC - extracellular concentration in the media at t=60 min
IC - intracellular concentration in the cells at t=60 min
Kp -intracellular accumulation ratio (IC/EC)

	Caco-2 permeability			
compound	P_{app} A-B x10^{-6} (cm/s)	P_{app} B-A x10^{-6} (cm/s)	Efflux ratio	Recovery (%)
**12l**	0.1 ± 0.0	0.1 ± 0.0	0.9	17
**12q**	0.2 ± 0.0	0.0 ± 0.0	0.4	4
**12r**	0.1 ± 0.1	0.1 ± 0.0	0.1	1
**12s**	0.4 ± 0.3	0.3 ± 0.1	1.3	3

Papp - apparent permeation coefficient

Table S3. Metabolic stability of selected compounds. The initial concentration of drug was 5uM (in triplicates).

compound	Plasma stability: Human $T_{1/2,}$ (min)	Plasma stability: Human Rem. at 120 min (%)	Plasma stability: Mouse $T_{1/2,}$ (min)	Plasma stability: Mouse Rem. at 120 min (%)
**12a**	**Stable**	84.3 ± 2.6	**Stable**	119.2 ± 6.0
**12e**	**Stable**	76.3 ± 13.7	**Stable**	120.8 ± 10.5
**12h**	**Stable**	102.3 ± 4.8	**Stable**	108.2 ± 4.2
**12l**	**Stable**	89.8 ± 2.0	**Stable**	109.9 ± 1.0
**12o**	**Stable**	95.6 ± 5.1	**Stable**	115.4 ± 3.7
**12q**	**Stable**	97.2 ± 4.1	**Stable**	113.2 ± 3.7
**12r**	**Stable**	89.7 ± 0.6	**Stable**	113.5 ± 6.0
**12s**	**Stable**	86.9 ± 0.9	**Stable**	102.7 ± 3.4

cmpd	Microsomal stability *: Human $T_{1/2,}$ (min)	Human Rem. at 45 min (%)	Human $Cl_{int,}$ (μl/min/mg)	Mouse $T_{1/2,}$ (min)	Mouse Rem. at 45 min (%)	Mouse $Cl_{int,}$ (μl/min/mg)
**12a**	17.3 ± 2.2	18.5 ± 4.0	**77.3 ± 10.3**	44.1 ± 2.7	48.7 ± 3.2	**32.6 ± 2.1**
**12e**	91.3 ± 12.2	60.2 ± 3.5	**19.6 ± 6.1**	**Stable**	79.3 ± 3.8	**-**
**12h**	38.0 ± 3.7	47.2 ± 3.5	**34.9 ± 4.0**	37.3 ± 1.8	43.0 ± 0.8	**37.8 ± 1.8**
**12l**	113.4 ± 3.5	78.6 ± 1.1	**11.2 ± 1.5**	33.7 ± 0.7	37.4 ± 0.5	**40.9 ± 0.8**
**12o**	26.2 ± 3.4	30.5 ± 5.3	**53.8 ± 5.8**	74.9 ± 7.5	65.9 ± 3.2	**19.2 ± 1.7**
**12q**	**Stable**	105.0 ± 3.9	**-**	**Stable**	101.0 ± 3.4	**-**
**12r**	**Stable**	83.3 ± 2.6	**-**	**Stable**	80.9 ± 12.0	**-**
**12s**	53.7 ± 2.7	55.9 ± 4.7	**27.1 ± 2.0**	50.8 ± 5.1	50.2 ± 3.2	**30.5 ± 4.7**

* 0.5 mg/ml of human or mouse microsomes

Table S4. Antiviral activity and cytotoxicity screening in Calu-3 and VeroE6 cells.[a]

	Calu-3				VeroE6			
Compound	EC_{50} [μM]	95% CI for EC_{50} [μM]	CC_{50} [μM]	95% CI for CC_{50} [μM]	EC_{50} [μM]	95% CI for EC_{50} [μM]	CC_{50} [μM]	95% CI for CC_{50} [μM]
**12a**	>100	n.a.	>100	n.a.	>100	n.a.	>100	n.a.
**12b**	>100	n.a.	>100	n.a.	>100	n.a.	>100	n.a.
**12c**	>100	n.a.	>100	n.a.	>100	n.a.	>100	n.a.
**12d**	>100	n.a.	>100	n.a.	>100	n.a.	>100	n.a.
**12e**	>100	n.a.	>100	n.a.	>100	n.a.	>100	n.a.
**12f**	>100	n.a.	>100	n.a.	>100	n.a.	>100	n.a.
**12g**	>100	n.a.	>100	n.a.	>100	n.a.	>100	n.a.
**12h**	>100	n.a.	>100	n.a.	>100	n.a.	>100	n.a.
**12i**	>100	n.a.	>100	n.a.	>100	n.a.	>100	n.a.
**12j**	>100	n.a.	>100	n.a.	>100	n.a.	>100	n.a.
**12k**	>100	n.a.	>100	n.a.	>100	n.a.	>100	n.a.
**12l**	>100	n.a.	>100	n.a.	>100	n.a.	>100	n.a.
**12m**	>100	n.a.	>100	n.a.	>100	n.a.	>100	n.a.
**12n**	>100	n.a.	>100	n.a.	>100	n.a.	>100	n.a.
**12o**	>100	n.a.	>100	n.a.	>100	n.a.	>100	n.a.
**12p**	>100	n.a.	>100	n.a.	>100	n.a.	>100	n.a.
**12q**	>100	n.a.	>100	n.a.	>100	n.a.	>100	n.a.
**12r**	>100	n.a.	>100	n.a.	>100	n.a.	>100	n.a.
**12s**	>100	n.a.	>100	n.a.	>100	n.a.	>100	n.a.
**TO507**	>100	n.a.	>100	n.a.	>100	n.a.	>100	n.a.
**3a**	>100	n.a.	>100	n.a.	>100	n.a.	>100	n.a.
Remdesivir	0.26	0.24 to 0.29	>50	n.a.	1.2	1.0 to 1.4	>50	n.a.

[a]Anti-SARS-CoV-2 activity in VeroE6 and Calu-3 cells, two-fold dilution in triplicate in 384 well from 100μM, using SARS-CoV-2 MOI~0.03, 3 days incubation. CPE evaluated by XTT assay. Cytotoxicity in VeroE6 and Calu-3 cells, same setup without virus. Remdesivir as a control drug, two-fold dilution from 50μM.

Table S5. Results of docking experiments in Autodock Vina 1.1.2 and 1.2.3 for all compounds in the main series.

Cmpd	Autodock 1.1 Score			Autodock 1.2 Score		
	Highest[a]	Pose A[b]	Pose B[b]	Highest[a]	Pose A[b]	Pose B[b]
**12a**	-10.9	-10.7/2	-10.9/1	-10.5	-10.4/2	-10.5/1
**12b**	-10.3	-10.3/1	-10.2/2	-10.1	-10.1/1	-9.9/3
**12c**	-10.6	-10.6/2	-10.6/1	-10.5	-10.4/2	-10.5/1
**12d**	-11.3	-11.3/1	-11.2/3	-10.8	-10.8/1	NI
**12e**	-11.2	-11.2/1	-11.0/2	-10.8	-10.8/1	-10.6/2
**12f**	-10.4	-10.3/2	-10.2/3	-10.6	-10.5/2	-10.4/3
**12g**	-10.4	-10.2/2	-10.1/3	-11.2	-11.0/2	-10.8/3
**12h**	-10.7	-10.7/1	-10.6/2	-10.3	-10.3/1	-10.1/2
**12i**	-10.6	-10.6/1	-10.5/2	-10.0	-9.9/3	-10.0/1
**12j**	-10.8	-10.5/2	-10.3/3	-10.7	-10.4/	-10.0/3
**12k**	-10.8	-10.4/2	-10.3/3	-10.6	-10.1/2	-10.0/3
**12l**	-10.4	10.2/2	-10.1/3	-10.1	-10.0/2	-9.9/3
**12m**	-10.3	-10.1/2	-10.0/3	-10.5	-10.4/2	-10.2/3
**12n**	-10.3	-10.2/2	-10.0/3	-10.0	-9.9/2	-9.8/3
**12q**	-10.8	-10.8/1	-10.6/2	-10.1	-10.1/1	-9.9/2
**12p**	-10.5	-10.5/1	-10.4/2	-10.4	-10.4/1	-10.2/2
**12q**	-11.4	-11.4/1	-11.3/2	-11.4	-11.4/1	-11.0/2
**12r**	-10.8	-10.8/1	-10.7/2	-10.9	-10.9/1	-10.9/2
**12s**	-10.3	-10.3/2	-10.2/3	-10.7	-10.7/2	-10.7/3

[a]The highest score achieved in the docking experiment.
[b]The score achieved for a given pose type in the docking experiment/ranking.

7. ^{1}H and ^{13}C NMR of final compounds

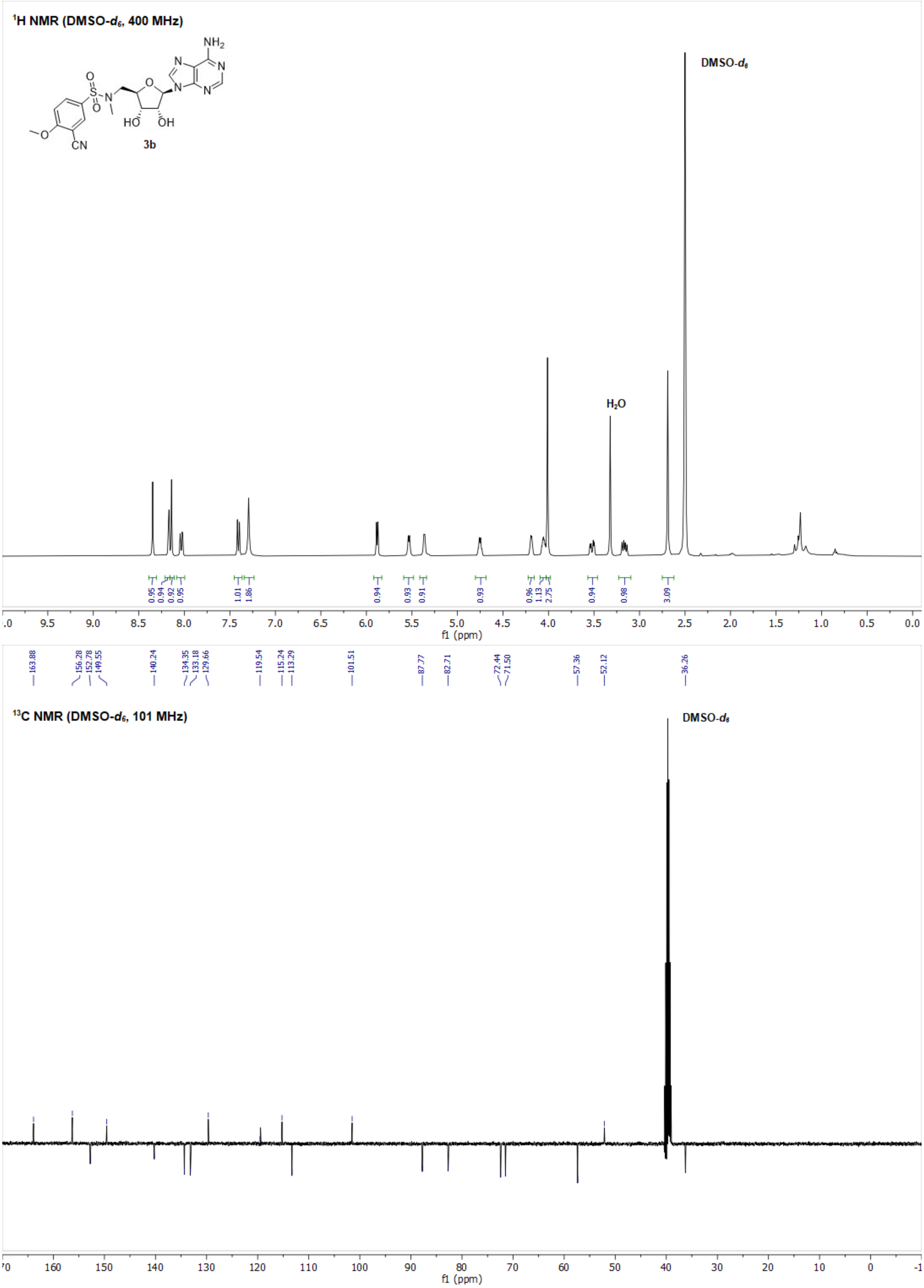

Figure S2. ^{1}H (top) and ^{13}C APT (bottom) NMR spectra of compound **3b** measured in DMSO-d_6.

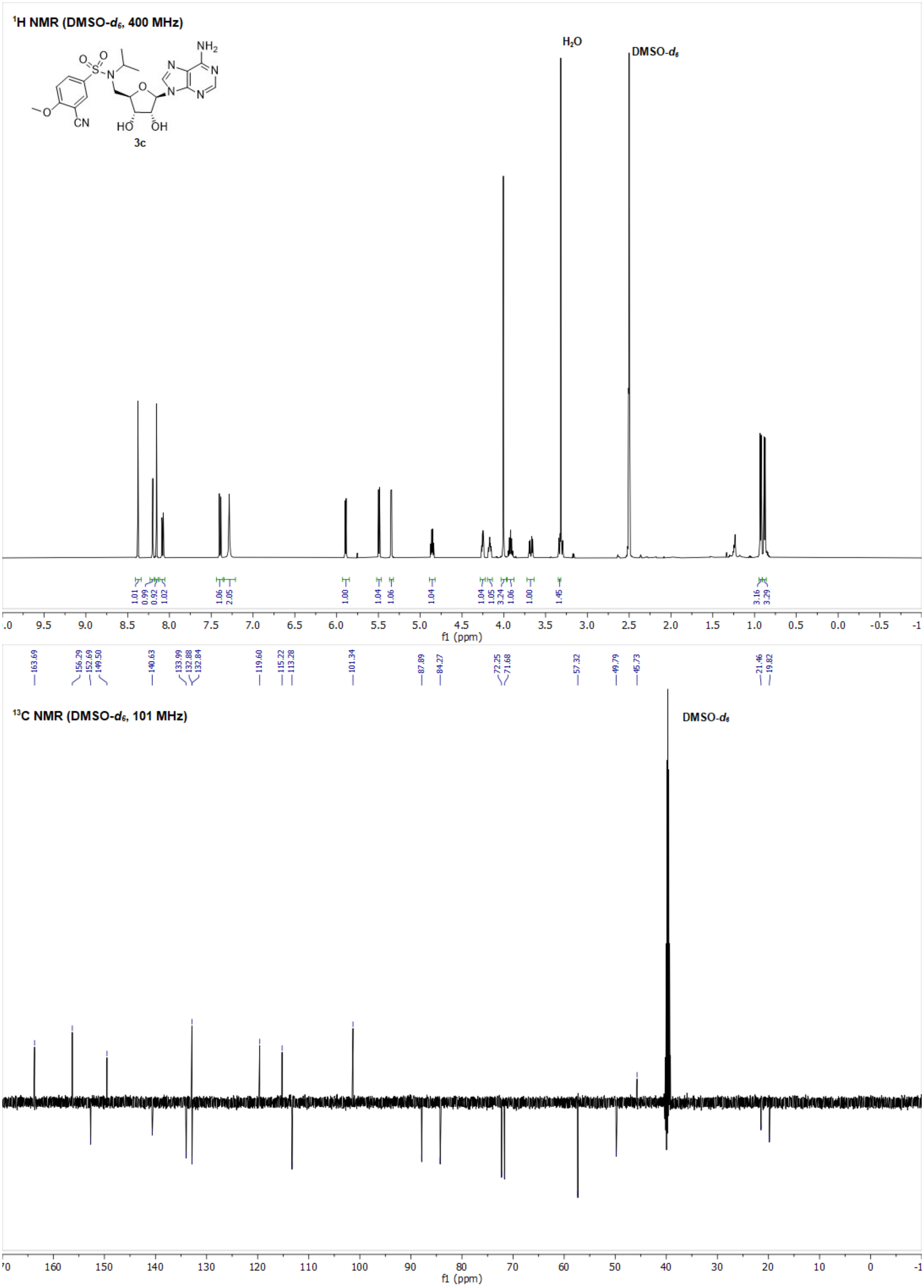

Figure S3. ^{1}H (top) and ^{13}C APT (bottom) NMR spectra of compound **3c** measured in DMSO-*d*$_6$.

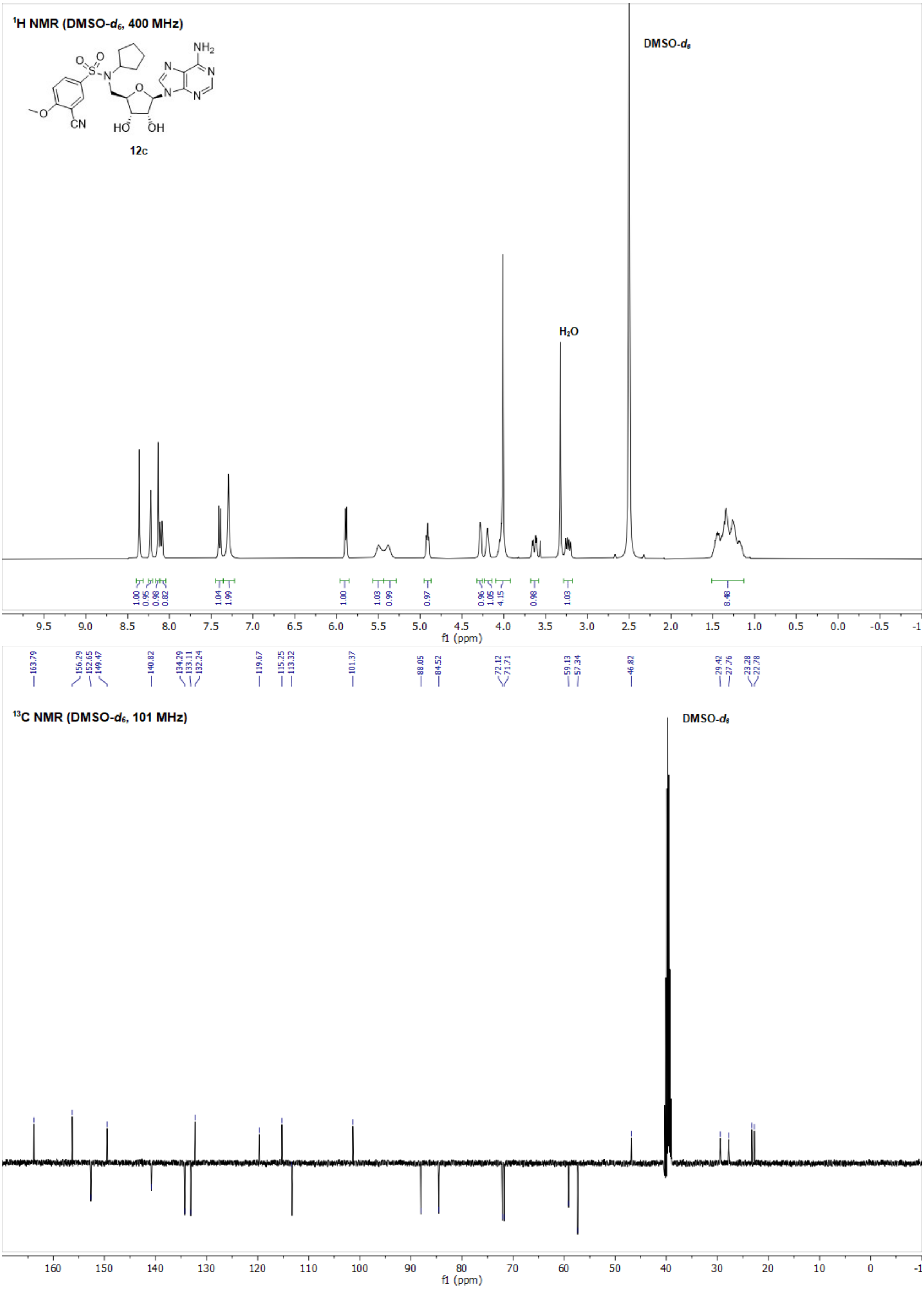

Figure S4. ^{1}H (top) and ^{13}C APT (bottom) NMR spectra of compound **3d** measured in DMSO-d_6.

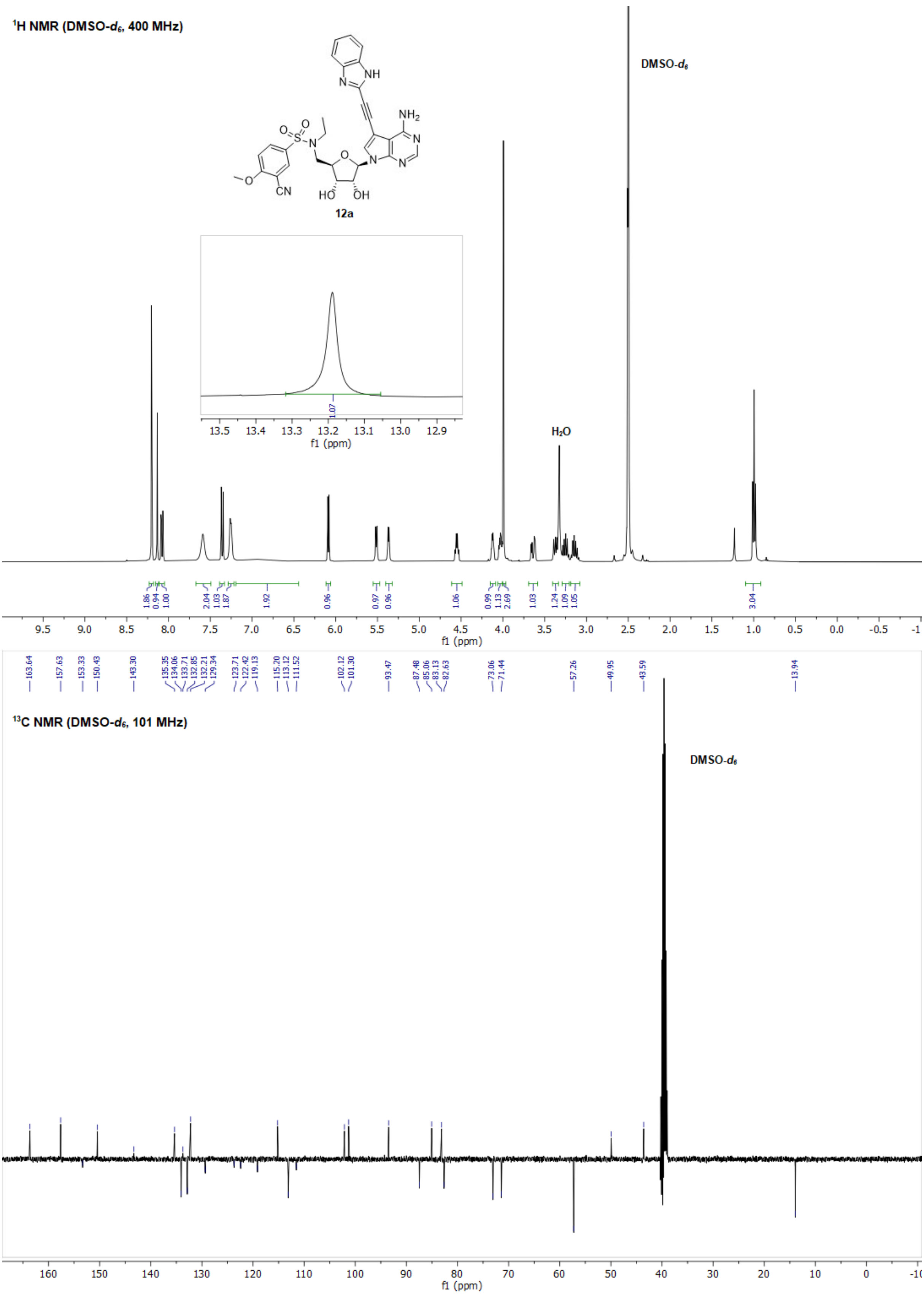

Figure S5. ¹H (top) and ¹³C APT (bottom) NMR spectra of compound **12a** measured in DMSO-d_6.

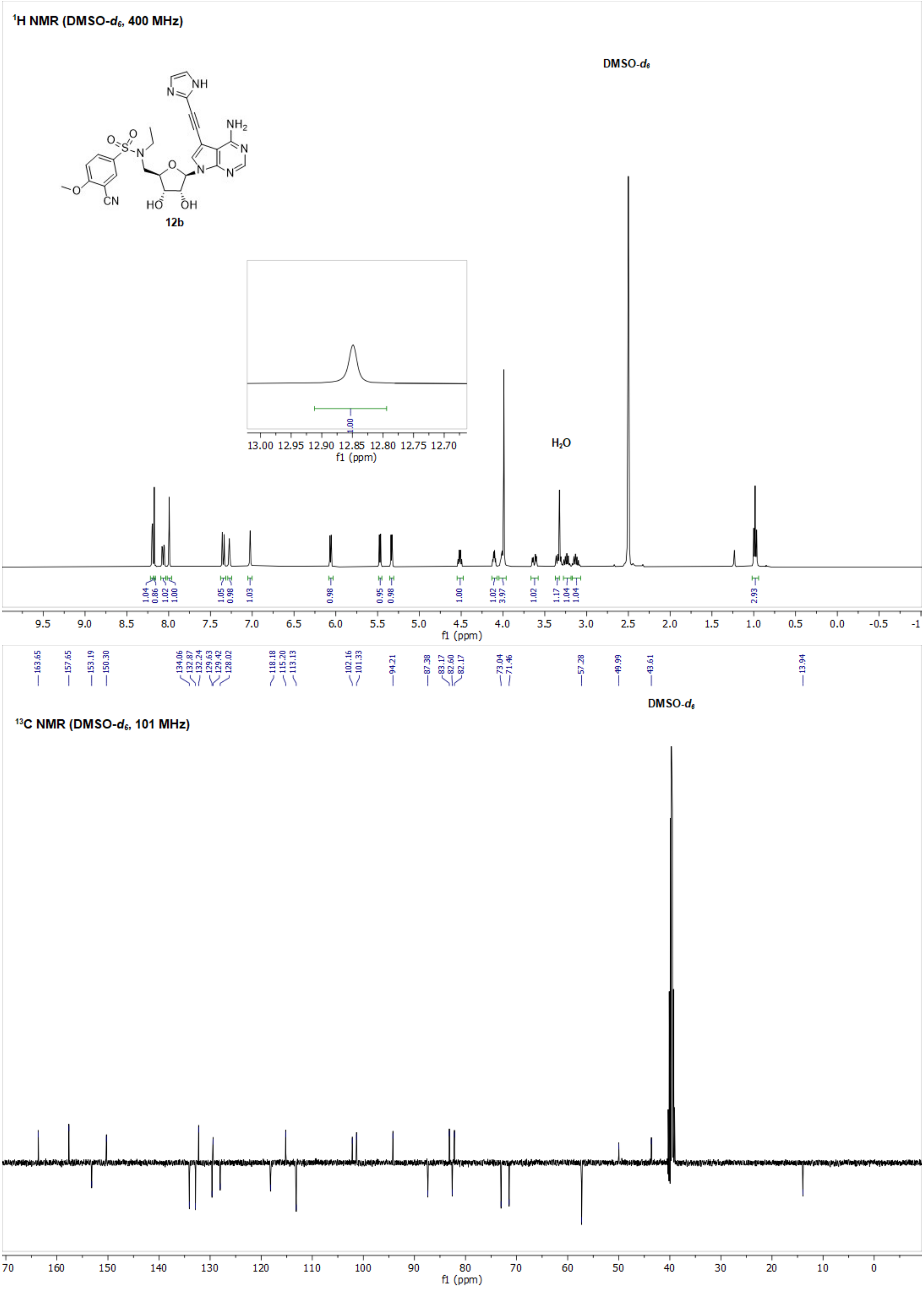

Figure S6. ^{1}H (top) and ^{13}C APT (bottom) NMR spectra of compound **12b** measured in DMSO-d_6.

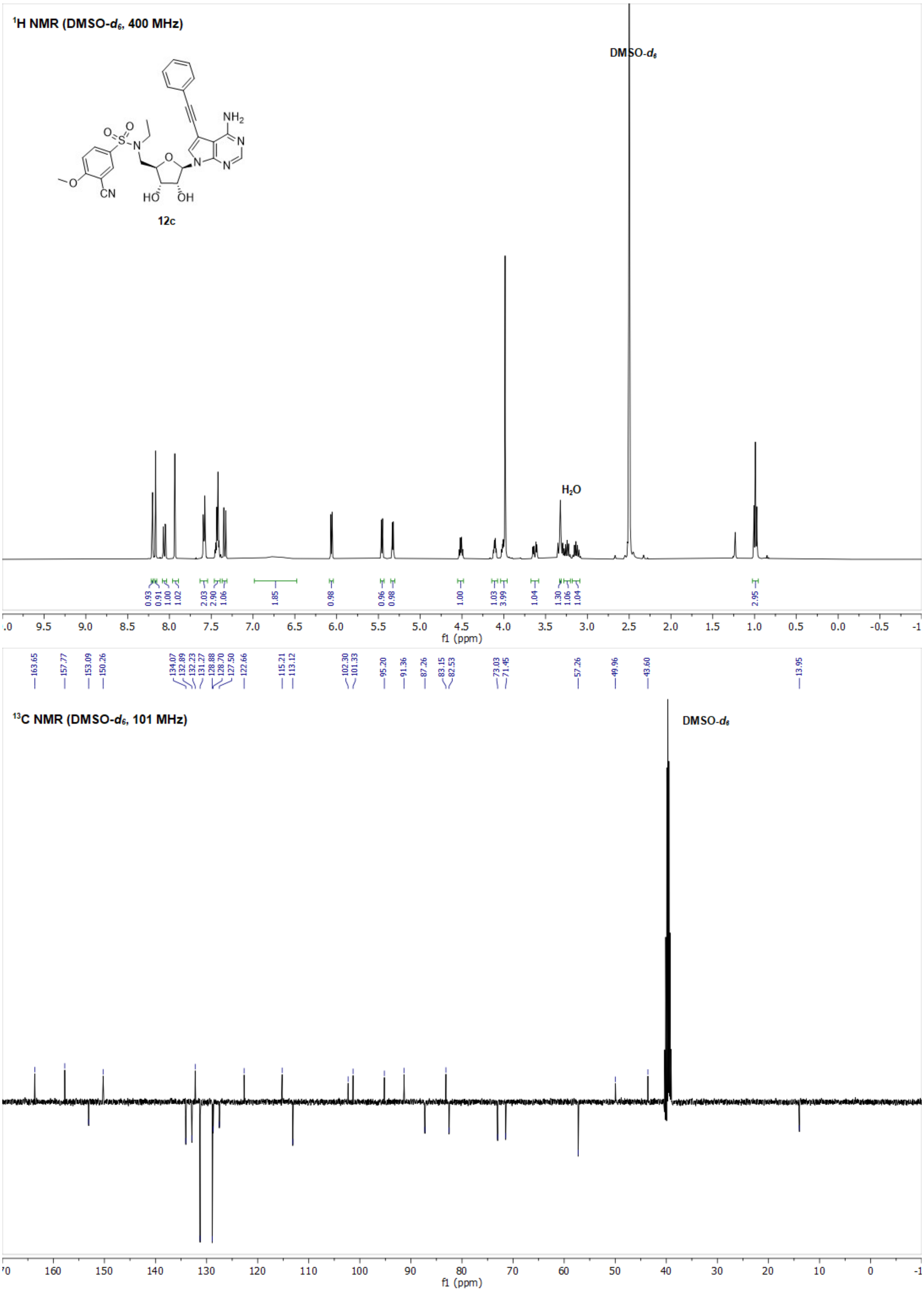

Figure S7. ^{1}H (top) and ^{13}C APT (bottom) NMR spectra of compound **12c** measured in DMSO-*d*6.

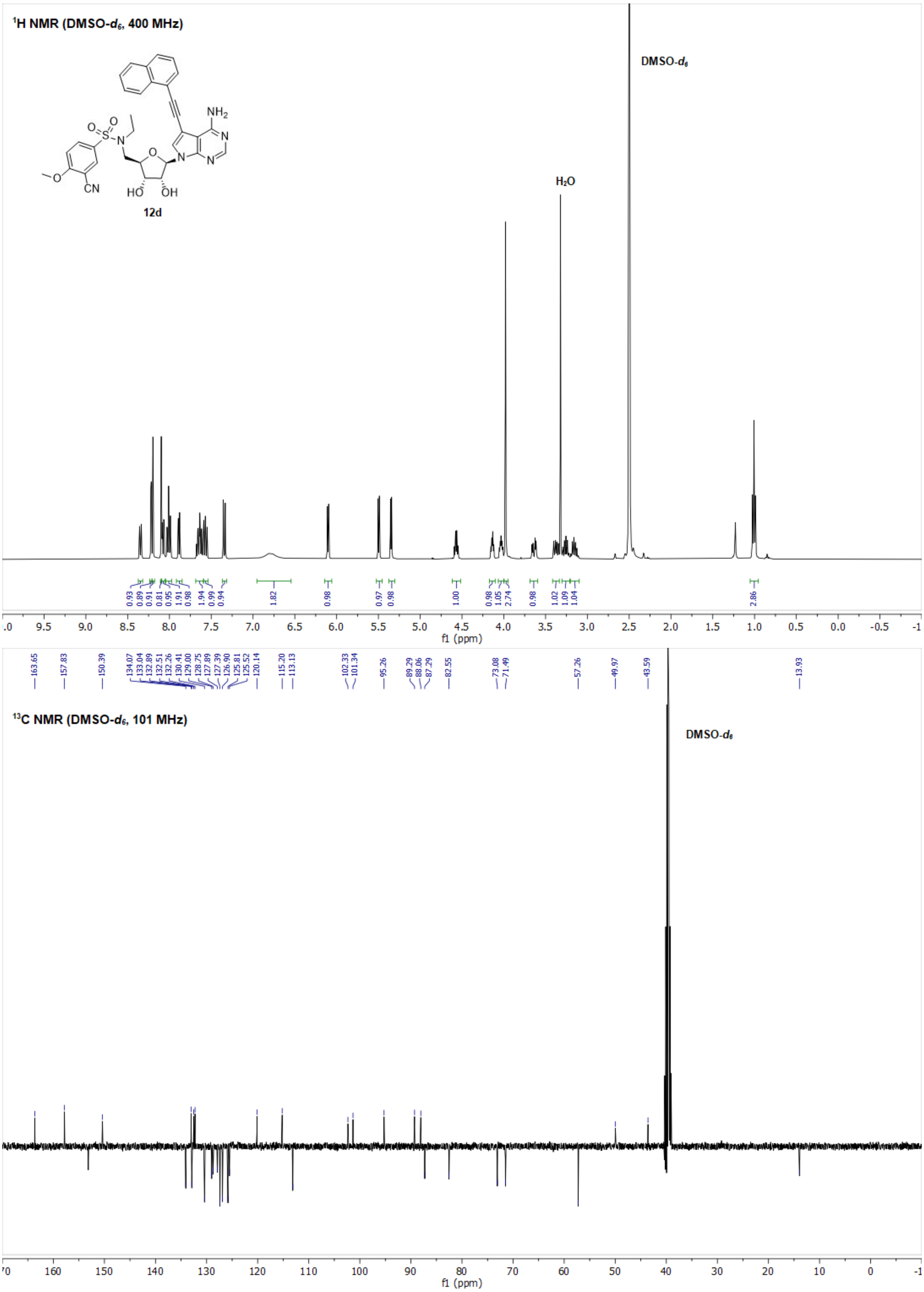

Figure S8. ^{1}H (top) and ^{13}C APT (bottom) NMR spectra of compound **12d** measured in DMSO-d_6.

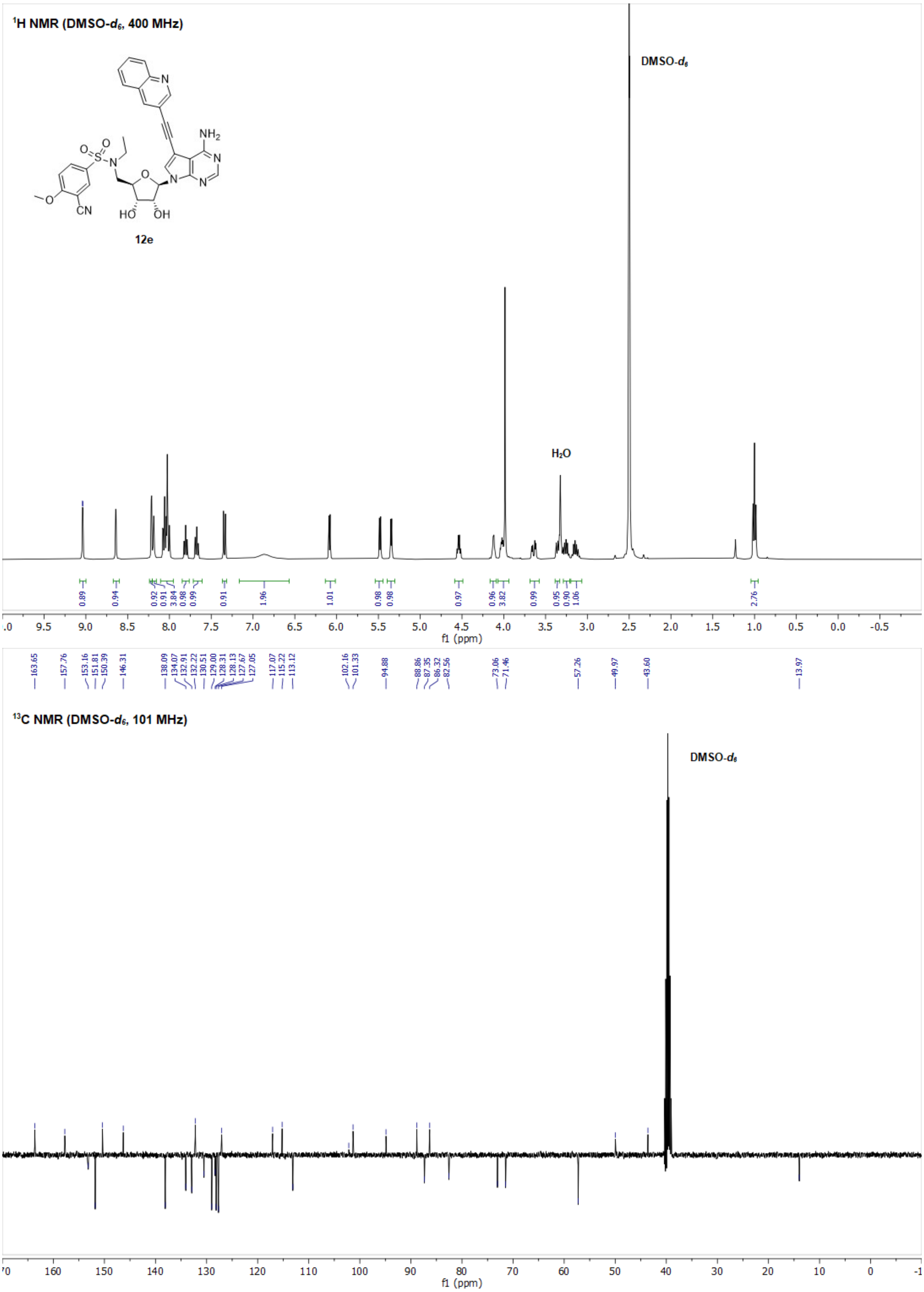

Figure S9. ^{1}H (top) and ^{13}C APT (bottom) NMR spectra of compound **12e** measured in DMSO-d_6.

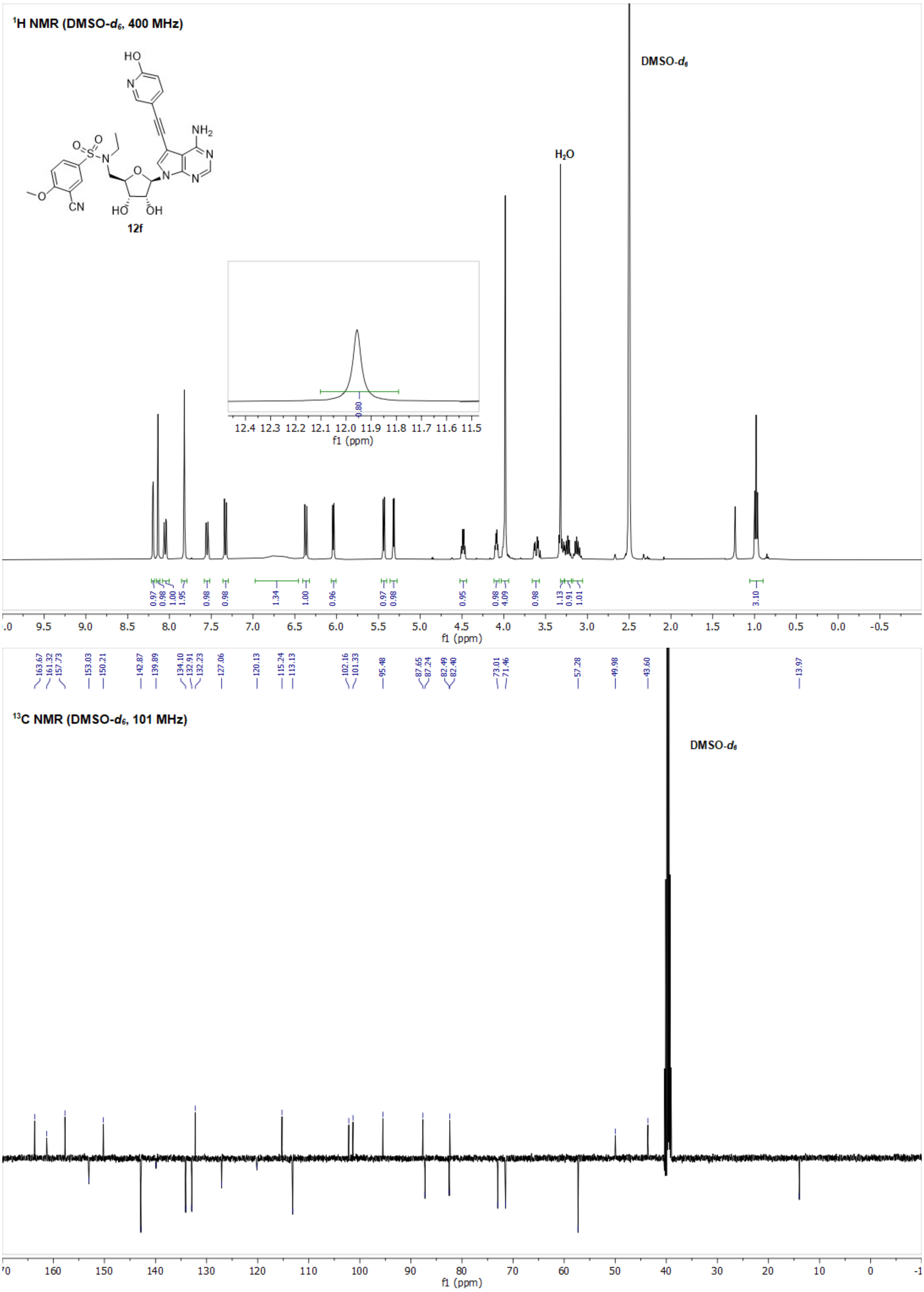

Figure S10. ^{1}H (top) and ^{13}C APT (bottom) NMR spectra of compound **12f** measured in DMSO-d_6.

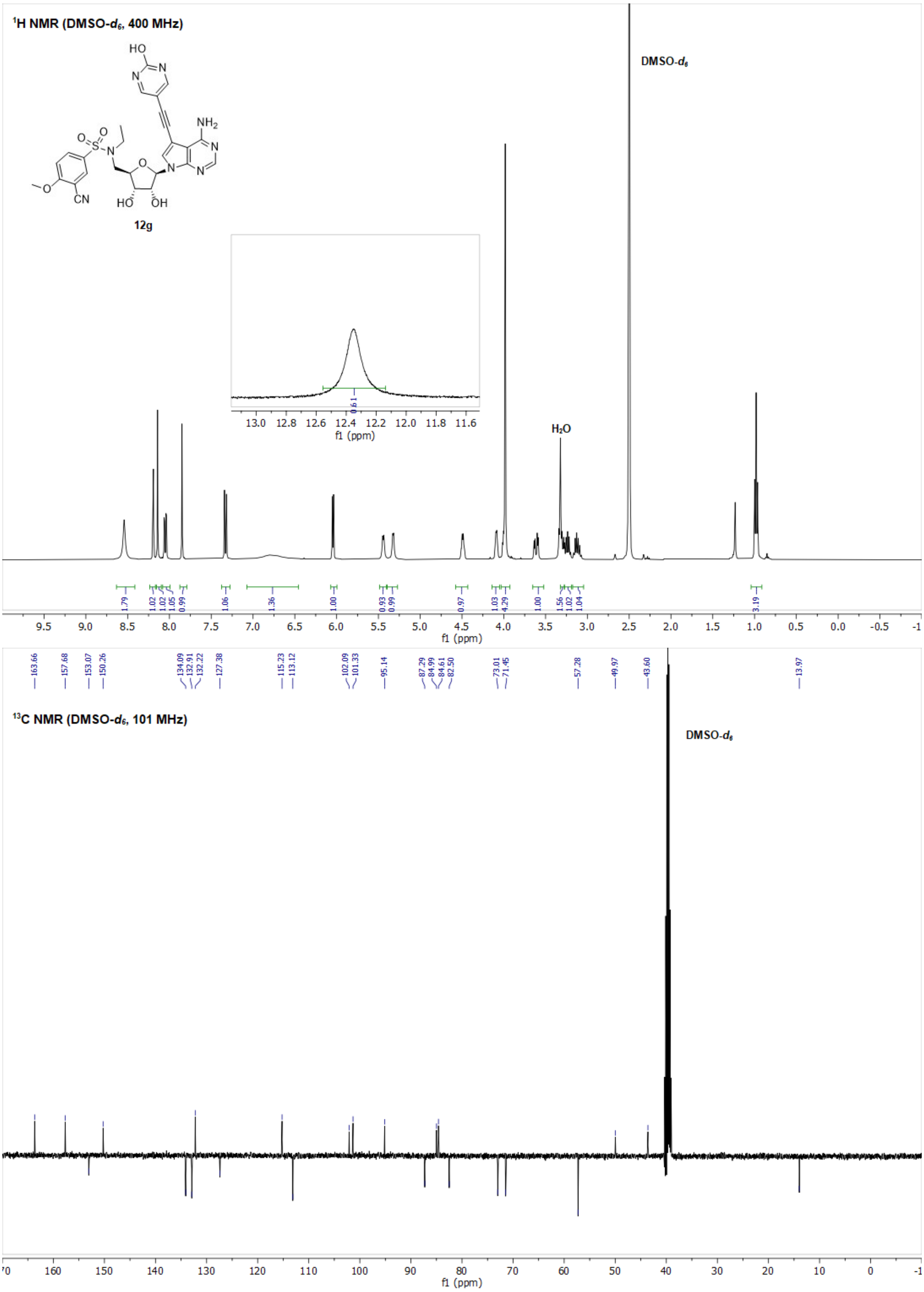

Figure S11. ^{1}H (top) and ^{13}C APT (bottom) NMR spectra of compound **12g** measured in DMSO-*d*6.

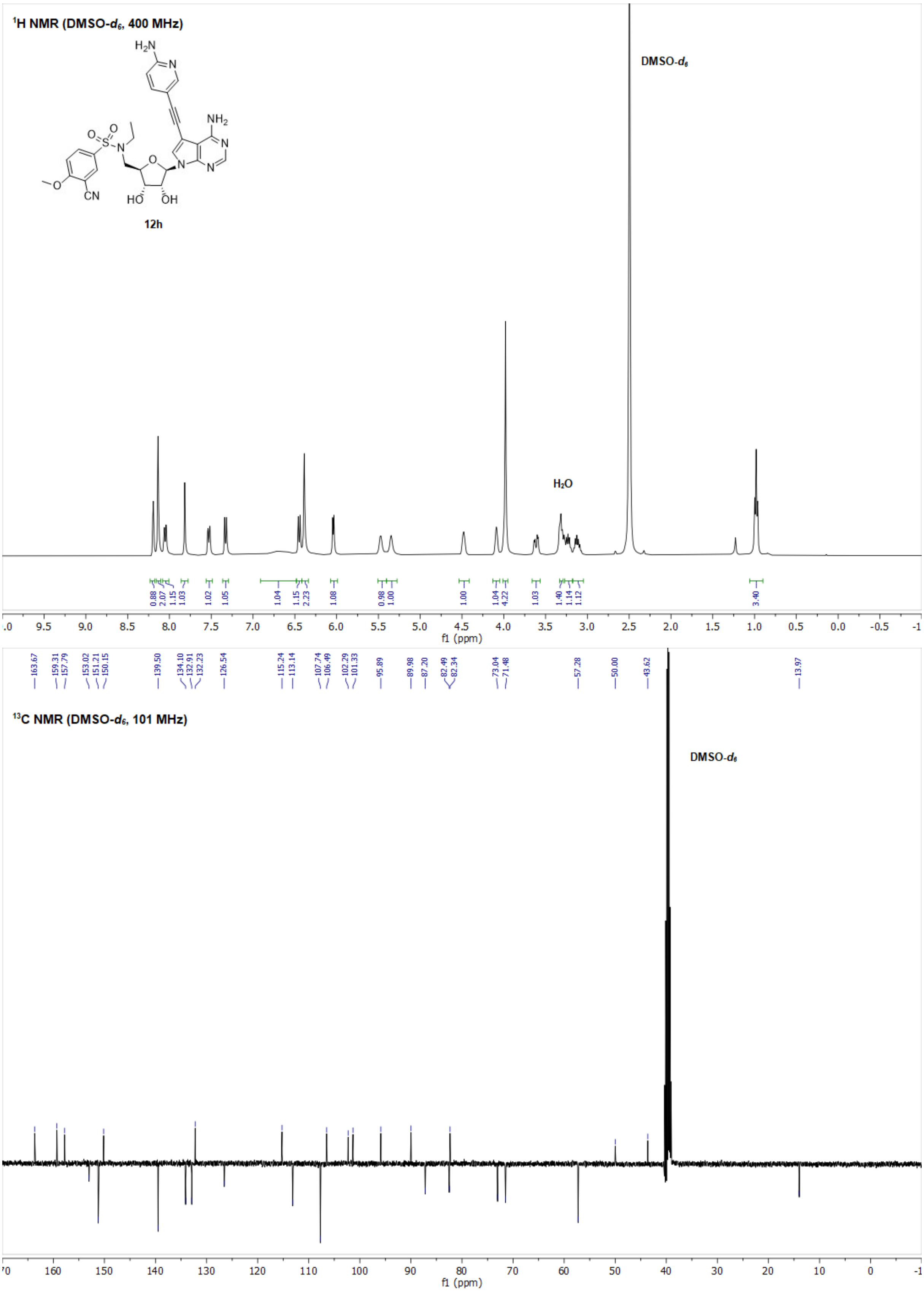

Figure S12. ^{1}H (top) and ^{13}C APT (bottom) NMR spectra of compound **12h** measured in DMSO-d_6.

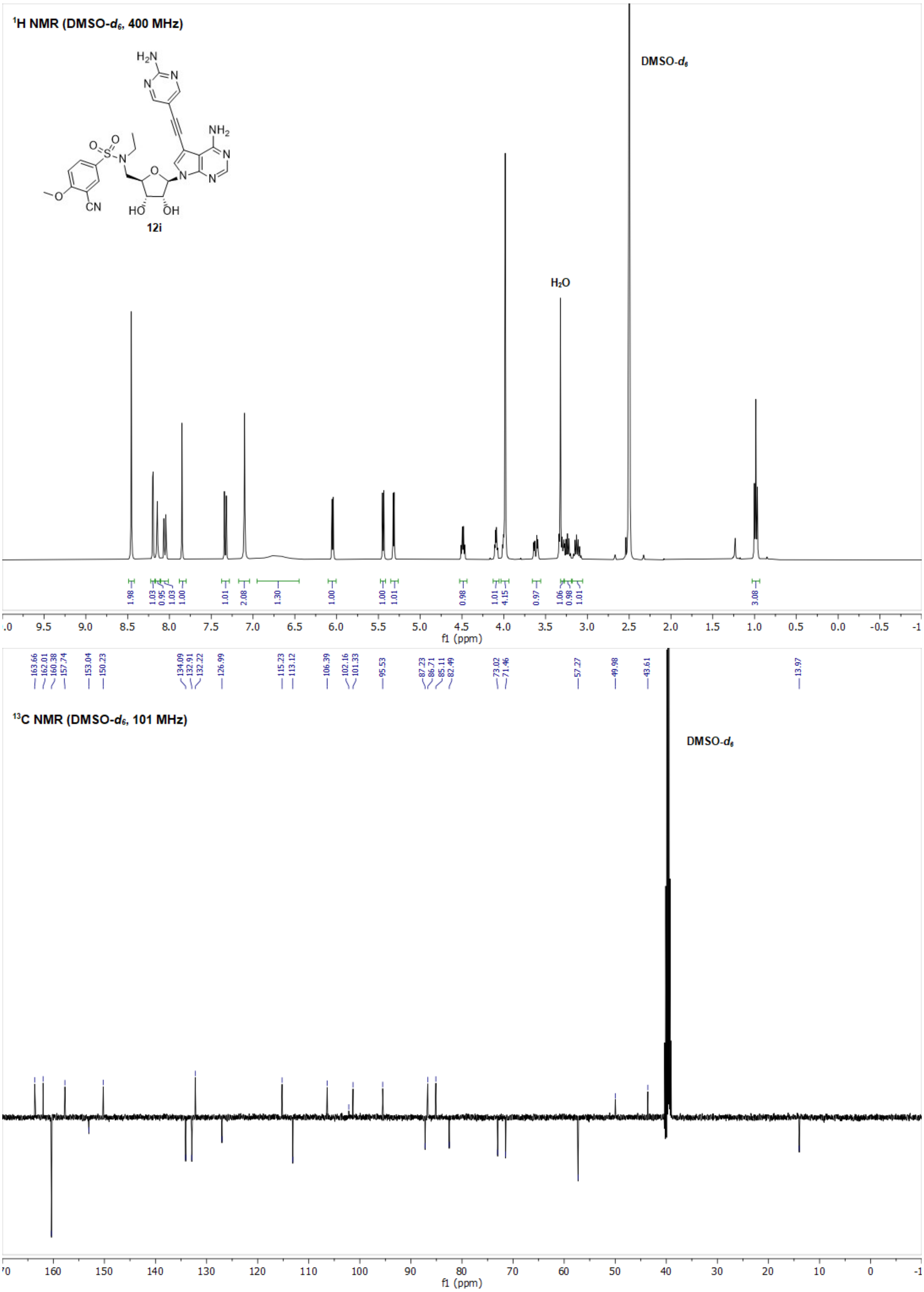

Figure S13. ^{1}H (top) and ^{13}C APT (bottom) NMR spectra of compound **12i** measured in DMSO-d_6.

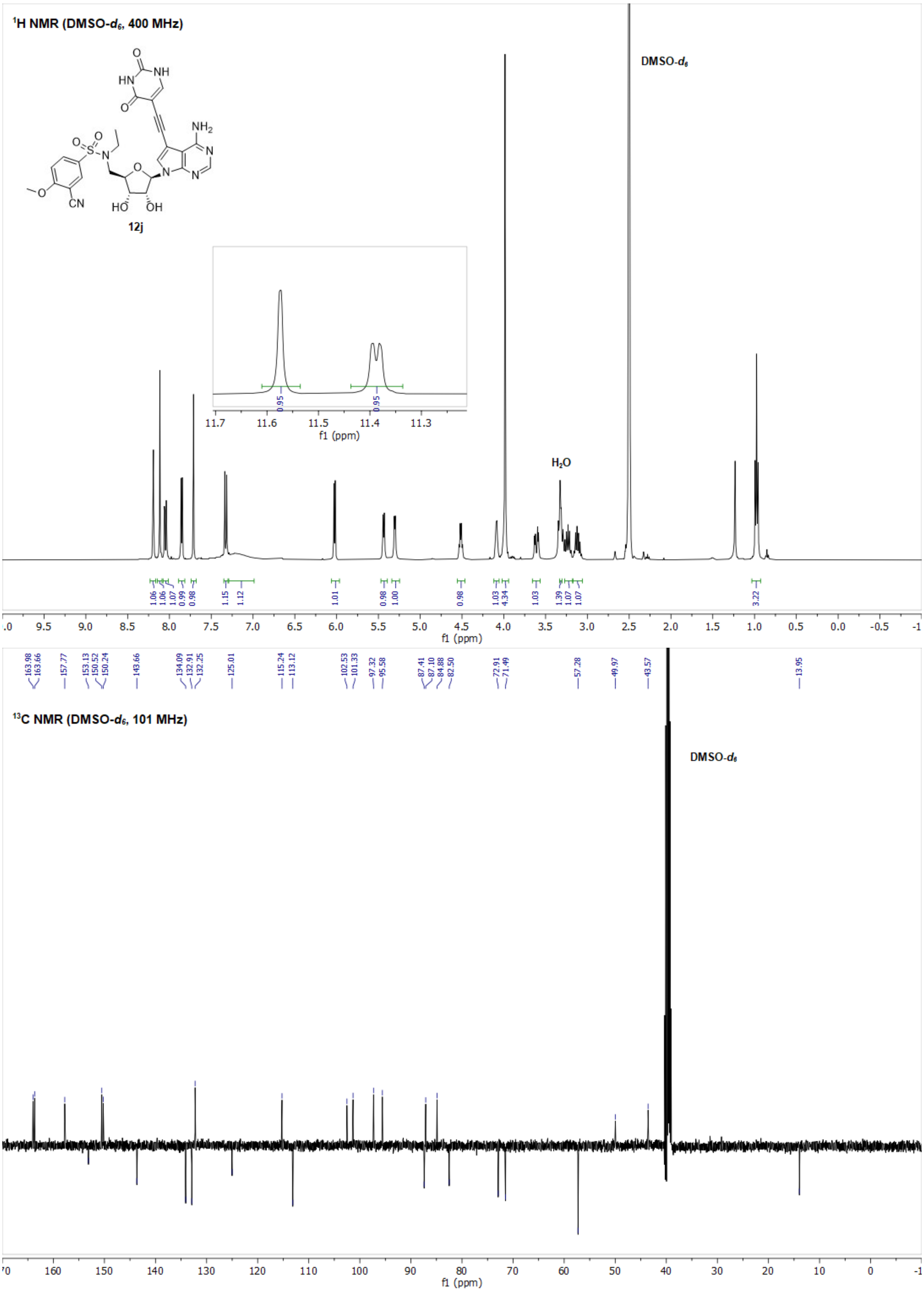

Figure S14. ^{1}H (top) and ^{13}C APT (bottom) NMR spectra of compound **12j** measured in DMSO-d_6.

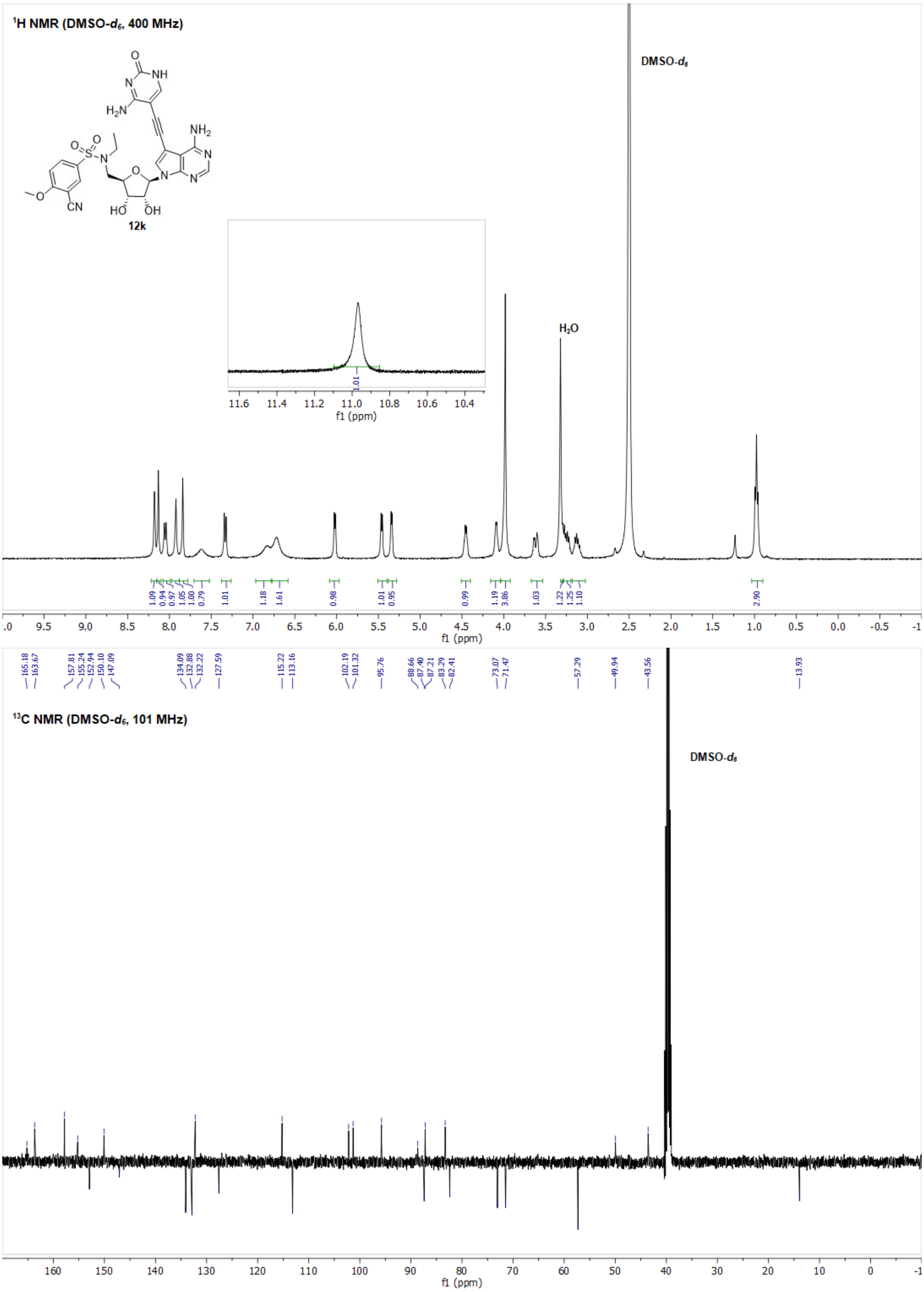

Figure S15. ^{1}H (top) and ^{13}C APT (bottom) NMR spectra of compound **12k** measured in DMSO-d_6.

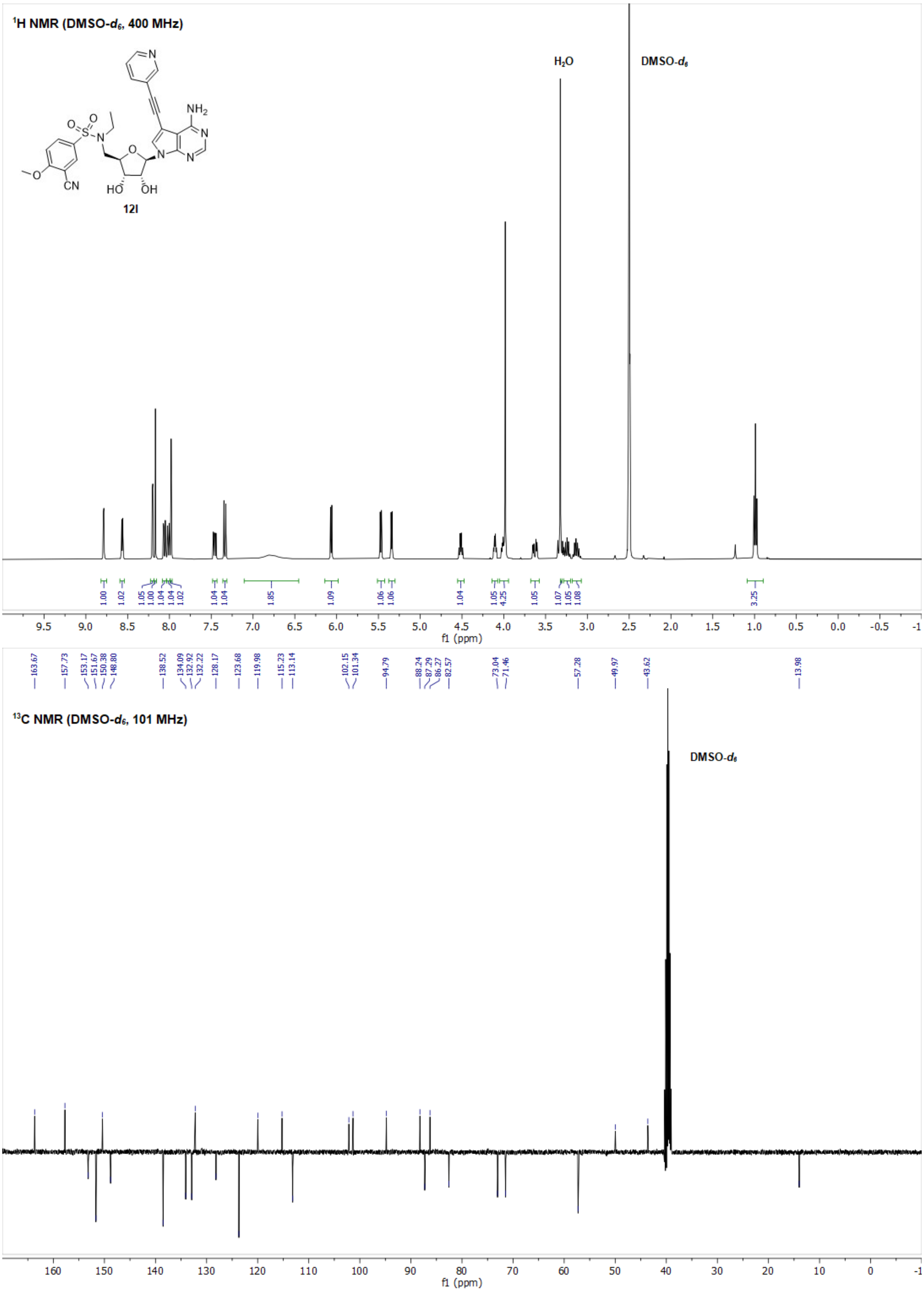

Figure S16. ^{1}H (top) and ^{13}C APT (bottom) NMR spectra of compound **12l** measured in DMSO-d_6.

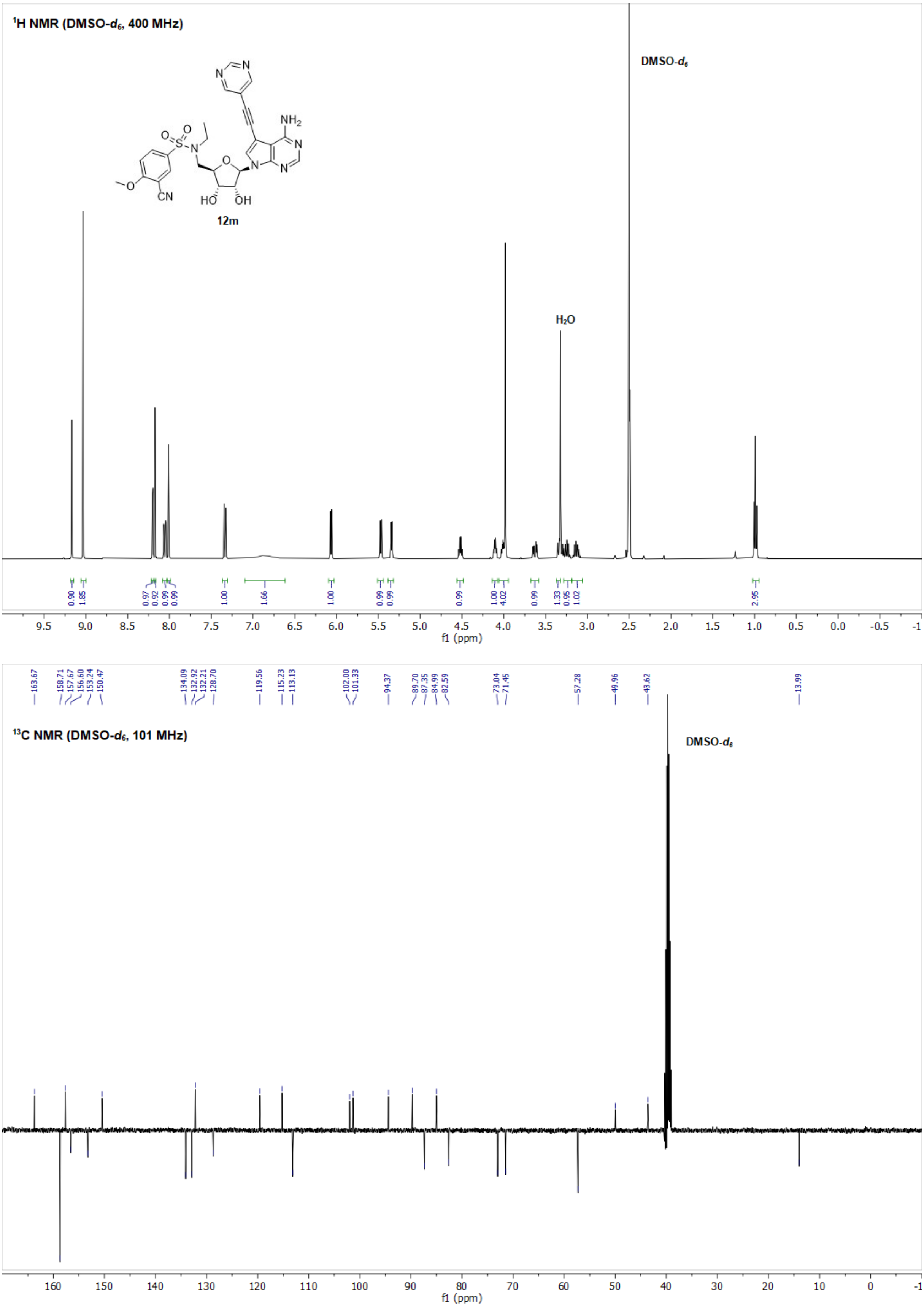

Figure S17. ^{1}H (top) and ^{13}C APT (bottom) NMR spectra of compound **12m** measured in DMSO-d_6.

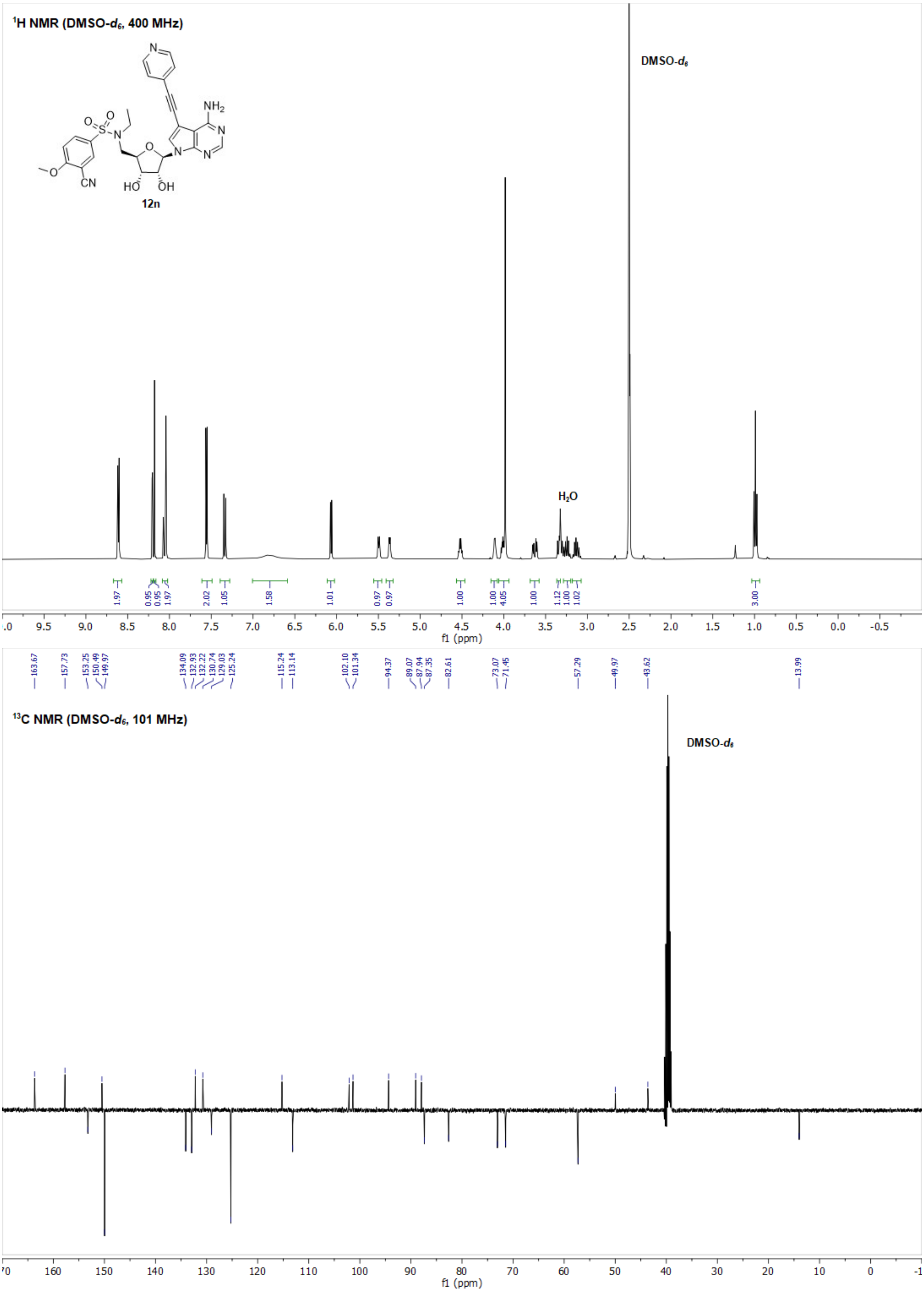

Figure S18. ^{1}H (top) and ^{13}C APT (bottom) NMR spectra of compound **12n** measured in DMSO-d_6.

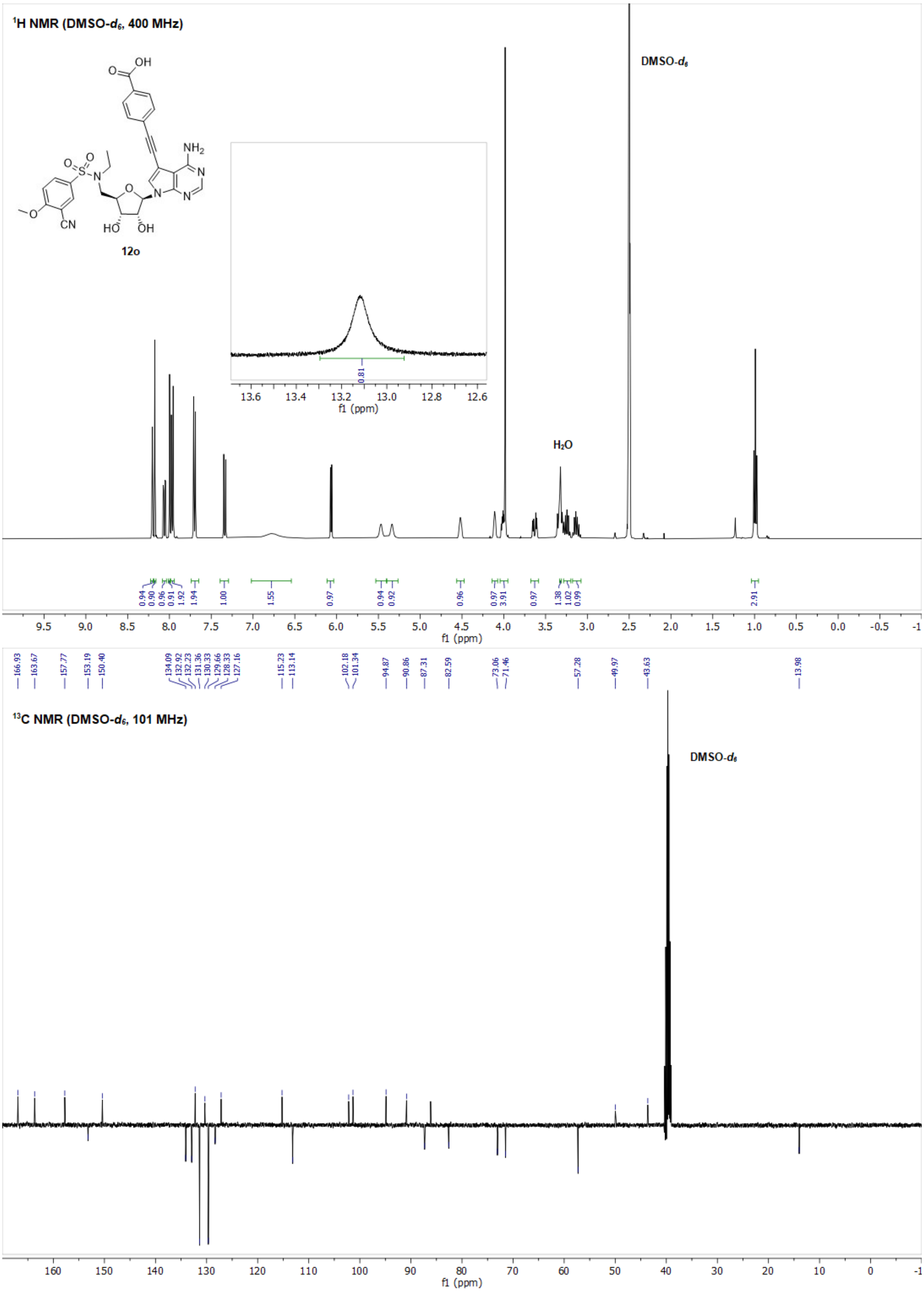

Figure S19. ^{1}H (top) and ^{13}C APT (bottom) NMR spectra of compound **12o** measured in DMSO-d_6.

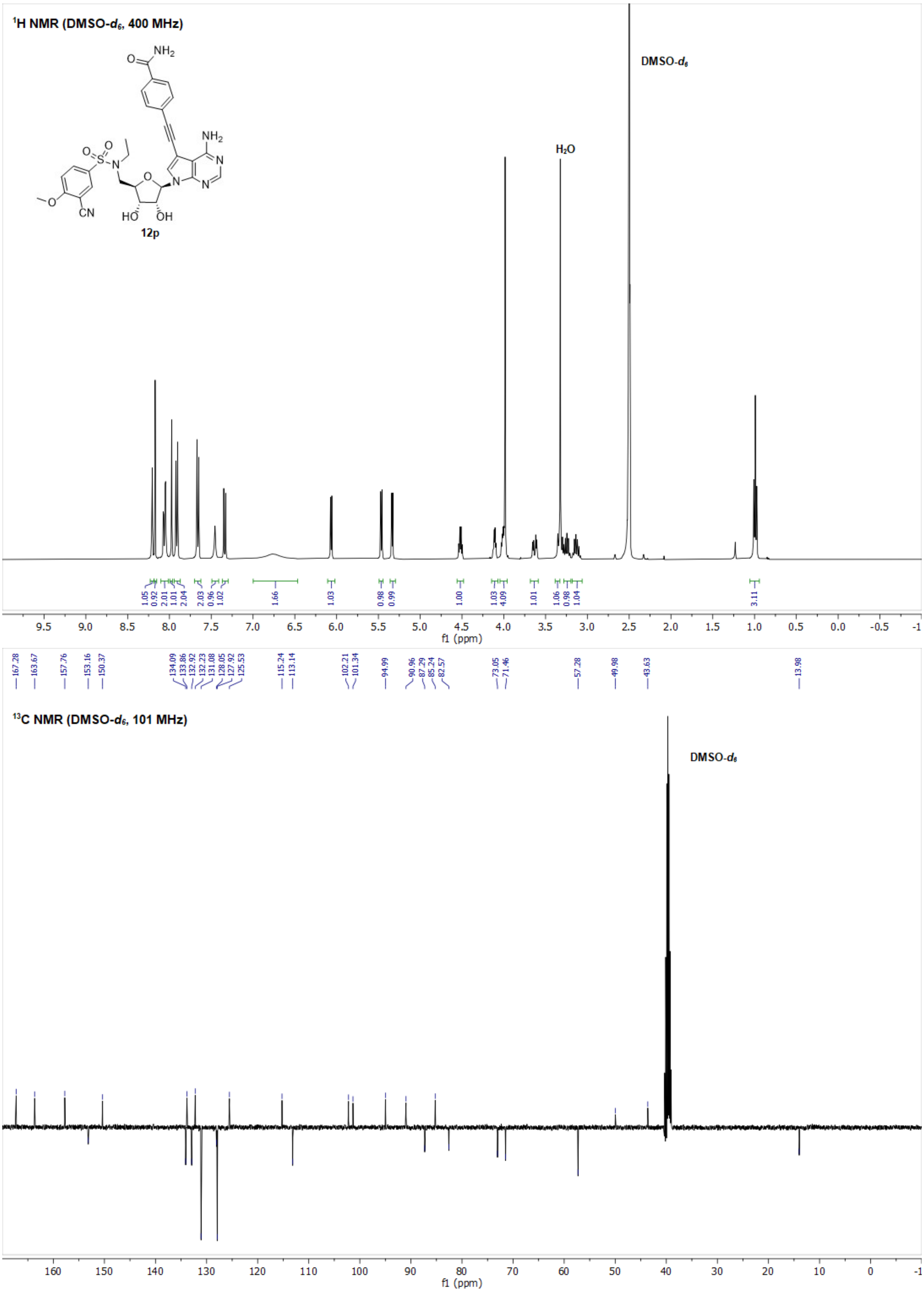

Figure S20. ^{1}H (top) and ^{13}C APT (bottom) NMR spectra of compound **12p** measured in DMSO-*d*$_6$.

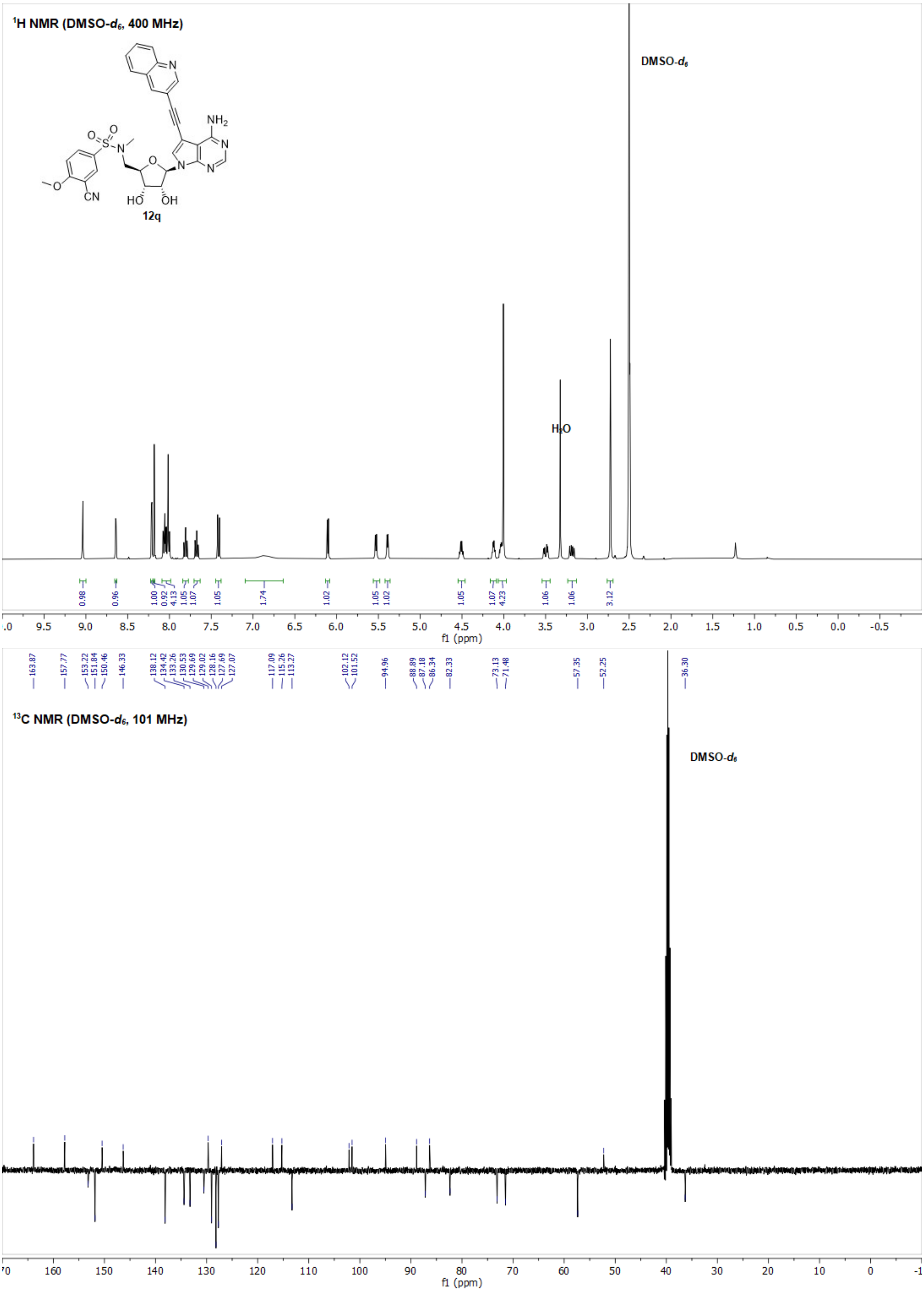

Figure S21. ^{1}H (top) and ^{13}C APT (bottom) NMR spectra of compound **12q** measured in DMSO-d_6.

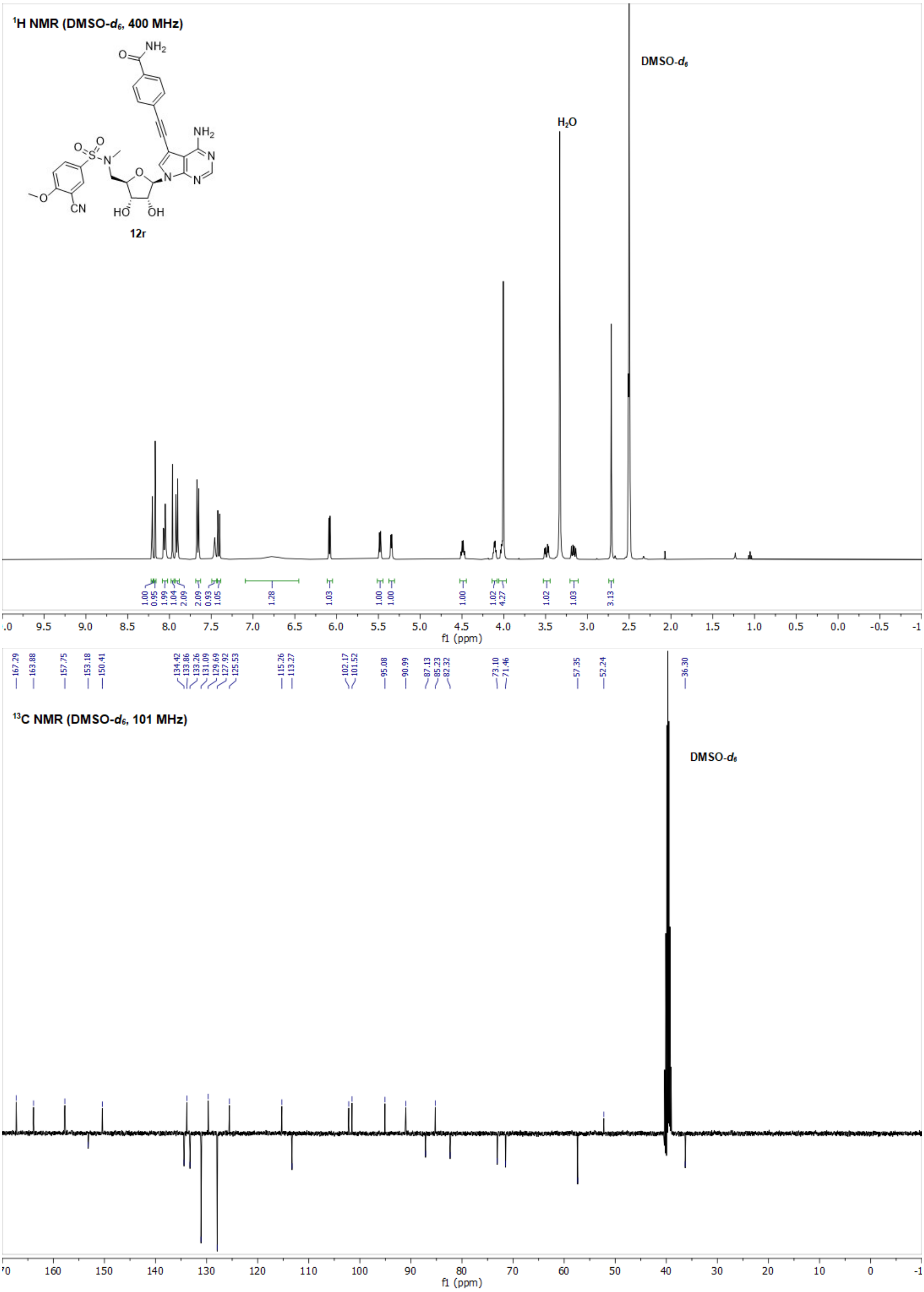

Figure S22. ^{1}H (top) and ^{13}C APT (bottom) NMR spectra of compound **12r** measured in DMSO-d_6.

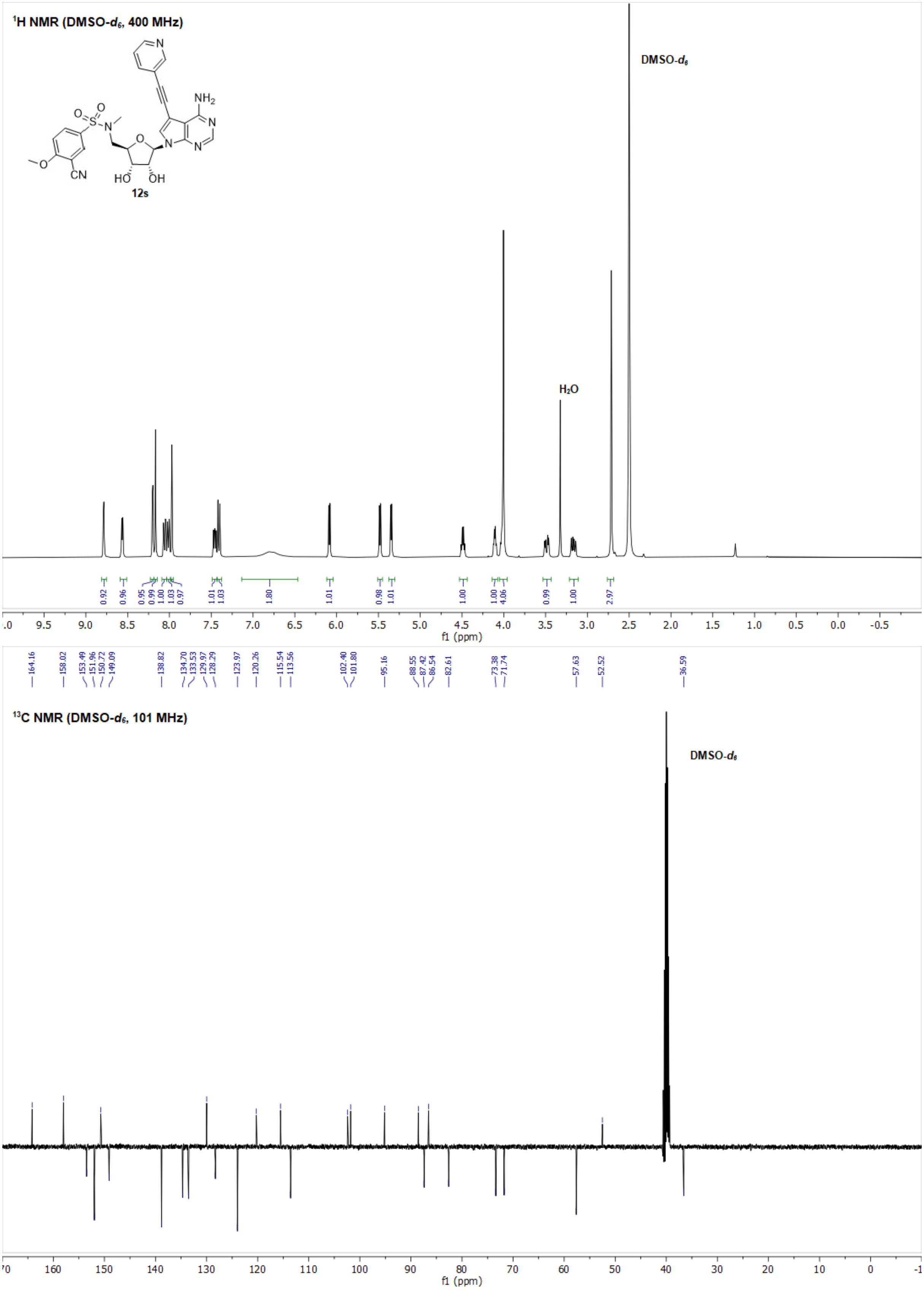

Figure S23. ^1H (top) and ^{13}C APT (bottom) NMR spectra of compound **12s** measured in DMSO-d_6.

8. HPLC purity of final compounds

The HPLC purity of final compounds **3a-d** and **12a-s** was measured at their absorbance maximum. The samples were prepared as solutions in methanol/DMSO mixture (8:2). Water with 0.05% FA was used as mobile phase A, and ACN with 0.05% FA was used as mobile phase B. Linear gradient of 5–95% of mobile phase B in mobile phase A over 7 minutes was used for the analysis.

Table S6. HPLC purities of final compounds, λ_{max} = absorption maximum, t_r = retention time

Compound	λ_{max} (nm)	t_r (min)	Purity (%)
**3a**	251	2.09	98.4
**3b**	249	1.97	99.5
**3c**	252	2.24	99.8
**3d**	252	2.50	99.9
**12a**	326	2.68	99.5
**12b**	243	1.85	99.1
**12c**	241	3.06	98.6
**12d**	305	3.42	99.7
**12e**	292	2.96	99.7
**12f**	245	2.16	98.7
**12g**	243	2.03	98.5
**12h**	300	1.85	97.8
**12i**	243	2.24	99.0
**12j**	230	2.06	96.1
**12k**	239	1.90	99.6
**12l**	300	2.45	99.9
**12m**	298	2.38	98.5
**12n**	359	2.18	99.8
**12o**	304	2.38	99.9
**12p**	305	2.66	99.5
**12q**	240	2.89	99.6
**12r**	243	2.29	99.4
**12s**	240	2.35	99.3

9. References

(1) Aakeroy, C. B.; Schultheiss, N.; Desper, J. Directed supramolecular assembly of Cu(II)-based "paddlewheels" into infinite 1-D chains using structurally bifunctional ligands. *Dalton Trans.* **2006**, (13), 1627-1635. DOI: 10.1039/b513765a
(2) Cee, V. J.; Albrecht, B. K.; Geuns-Meyer, S.; Hughes, P.; Bellon, S.; Bready, J.; Caenepeel, S.; Chaffee, S. C.; Coxon, A.; Emery, M.; et al. Alkynylpyrimidine amide derivatives as potent, selective, and orally active inhibitors of Tie-2 kinase. *J. Med. Chem.* **2007**, *50* (4), 627-640. DOI: 10.1021/jm061112p
(3) Ince, M.; Medina, A.; Yum, J. H.; Yella, A.; Claessens, C. G.; Martinez-Diaz, M. V.; Gratzel, M.; Nazeeruddin, M. K.; Torres, T. Peripherally and axially carboxylic acid substituted subphthalocyanines for dye-sensitized solar cells. *Chem. Eur. J.* **2014**, *20* (7), 2016-2021. DOI: 10.1002/chem.201303639
(4) Janeba, Z.; Balzarini, J.; Andrei, G.; Snoeck, R.; De Clercq, E.; Robins, M. J. Synthesis and biological evaluation of 5-(alkyn-1-yl)-1-(p-toluenesulfonyl)uracil derivatives. *Can. J. Chem.* **2006**, *84* (4), 580-586. DOI: 10.1139/V06-041
(5) Kielkowski, P.; Pohl, R.; Hocek, M. Synthesis of acetylene linked double-nucleobase nucleos(t)ide building blocks and polymerase construction of DNA containing cytosines in the major groove. *J. Org. Chem.* **2011**, *76* (9), 3457-3462. DOI: 10.1021/jo200436j
(6) Mohy El Dine, T.; Jimmidi, R.; Diaconu, A.; Fransolet, M.; Michiels, C.; De Winter, J.; Gillon, E.; Imberty, A.; Coenye, T.; Vincent, S. P. Pillar[5]arene-Based Polycationic Glyco[2]rotaxanes Designed as Pseudomonas aeruginosa Antibiofilm Agents. *J. Med. Chem.* **2021**, *64* (19), 14728-14744. DOI: 10.1021/acs.jmedchem.1c01241
(7) Nguyen, N. H.; Apriletti, J. W.; Baxter, J. D.; Scanlan, T. S. Hammett analysis of selective thyroid hormone receptor modulators reveals structural and electronic requirements for hormone antagonists. *J. Am. Chem. Soc.* **2005**, *127* (13), 4599-4608. DOI: 10.1021/ja0440093
(8) Otava, T.; Sala, M.; Li, F.; Fanfrlik, J.; Devkota, K.; Perveen, S.; Chau, I.; Pakarian, P.; Hobza, P.; Vedadi, M.; et al. The Structure-Based Design of SARS-CoV-2 nsp14 Methyltransferase Ligands Yields Nanomolar Inhibitors. *ACS Infect Dis* **2021**, *7* (8), 2214-2220. DOI: 10.1021/acsinfecdis.1c00131
(9) Ren, J.; Larkin, E.; Delaney, C.; Song, Y.; Jin, X.; Amirjalayer, S.; Bakker, A.; Du, S.; Gao, H.; Zhang, Y. Y.; et al. Chemistry of 4-[(4-bromophenyl)ethynyl]pyridine at metal surfaces studied by STM. *Chem. Commun.* **2018**, *54* (67), 9305-9308. DOI: 10.1039/c8cc03986k
(10) Richardson, C.; Reed, C. A. Synthesis of meso-extended tetraarylporphyrins. *J. Org. Chem.* **2007**, *72* (13), 4750-4755. DOI: 10.1021/jo070191p
(11) Ritson, D. J.; Moses, J. E. A fragment based click chemistry approach towards hybrid G-quadruplex ligands: design, synthesis and biophysical evaluation. *Tetrahedron* **2012**, *68* (1), 197-203. DOI: 10.1016/j.tet.2011.10.066
(12) Rodriguez, J. G.; MartinVillamil, R.; Cano, F. H.; Fonseca, I. Synthesis of 1,4di(n-pyridyl)buta-1,3-diyne and formation of charge-transfer complexes. X-Ray structure of 1,4-di(3-pyridyl)buta-1,3-diyne. *J. Chem. Soc. Perkin 1* **1997**, (5), 709-714. DOI: DOI 10.1039/a605468d
(13) Reddy, O. S.; Suryanarayana, C. V.; Narayana, K. J. P.; Anuradha, V.; Babu, B. H. Synthesis and cytotoxic evaluation for some new 2,5-disubstituted pyrimidine derivatives for anticancer activity. *Med. Chem. Res.* **2015**, *24* (5), 1777-1788. DOI: 10.1007/s00044-014-1276-6
(14) Ahmed-Belkacem, R.; Hausdorff, M.; Delpal, A.; Sutto-Ortiz, P.; Colmant, A. M. G.; Touret, F.; Ogando, N. S.; Snijder, E. J.; Canard, B.; Coutard, B.; et al. Potent Inhibition of SARS-CoV-2 nsp14 *N*7-Methyltransferase by Sulfonamide-Based Bisubstrate Analogues. *J. Med. Chem.* **2022**, *65* (8), 6231-6249. DOI: 10.1021/acs.jmedchem.2c00120
(15) Scheer, S.; Ackloo, S.; Medina, T. S.; Schapira, M.; Li, F.; Ward, J. A.; Lewis, A. M.; Northrop, J. P.; Richardson, P. L.; Kaniskan, H. U.; et al. A chemical biology toolbox to study protein methyltransferases and epigenetic signaling. *Nat Commun* **2019**, *10* (1), 19. DOI: 10.1038/s41467-018-07905-4
(16) Olšanská, L.; Cihlar, T.; Votruba, I.; Holý, A. Transport of Adefovir (PMEA) in Human T-Lymphoblastoid Cells. *Collect. Czech. Chem. Commun.* **1997**, *62* (5), 821-828. DOI: 10.1135/cccc19970821
(17) Šmídková, M.; Dvoráková, A.; Tloust'ová, E.; Česnek, M.; Janeba, Z.; Mertlíková-Kaiserová, H. Amidate prodrugs of 9-[2-(phosphonomethoxy)ethyl]adenine as inhibitors of adenylate cyclase toxin

from Bordetella pertussis. *Antimicrob. Agents Chemother.* **2014**, *58* (2), 664-671. DOI: 10.1128/aac.01685-13
(18) Jones, G.; Willett, P.; Glen, R. C.; Leach, A. R.; Taylor, R. Development and validation of a genetic algorithm for flexible docking. *J. Mol. Biol.* **1997**, *267* (3), 727-748. DOI: 10.1006/jmbi.1996.0897
(19) Czarna, A.; Plewka, J.; Kresik, L.; Matsuda, A.; Karim, A.; Robinson, C.; O'Byrne, S.; Cunningham, F.; Georgiou, I.; Wilk, P.; et al. Refolding of lid subdomain of SARS-CoV-2 nsp14 upon nsp10 interaction releases exonuclease activity. *Structure* **2022**, *30* (8), 1050-1054 e1052. DOI: 10.1016/j.str.2022.04.014
(20) Trott, O.; Olson, A. J. AutoDock Vina: improving the speed and accuracy of docking with a new scoring function, efficient optimization, and multithreading. *J. Comput. Chem.* **2010**, *31* (2), 455-461. DOI: 10.1002/jcc.21334
(21) Eberhardt, J.; Santos-Martins, D.; Tillack, A. F.; Forli, S. AutoDock Vina 1.2.0: New Docking Methods, Expanded Force Field, and Python Bindings. *J. Chem. Inf. Model.* **2021**, *61* (8), 3891-3898. DOI: 10.1021/acs.jcim.1c00203
(22) Mejdrova, I.; Chalupska, D.; Kogler, M.; Sala, M.; Plackova, P.; Baumlova, A.; Hrebabecky, H.; Prochazkova, E.; Dejmek, M.; Guillon, R.; et al. Highly Selective Phosphatidylinositol 4-Kinase IIIbeta Inhibitors and Structural Insight into Their Mode of Action. *J. Med. Chem.* **2015**, *58* (9), 3767-3793. DOI: 10.1021/acs.jmedchem.5b00499
(23) Mejdrova, I.; Chalupska, D.; Plackova, P.; Muller, C.; Sala, M.; Klima, M.; Baumlova, A.; Hrebabecky, H.; Prochazkova, E.; Dejmek, M.; et al. Rational Design of Novel Highly Potent and Selective Phosphatidylinositol 4-Kinase IIIbeta (PI4KB) Inhibitors as Broad-Spectrum Antiviral Agents and Tools for Chemical Biology. *J. Med. Chem.* **2017**, *60* (1), 100-118. DOI: 10.1021/acs.jmedchem.6b01465
